# Long noncoding RNA GSEC promotes neutrophil inflammatory activation by supporting PFKFB3-involved glycolytic metabolism in sepsis

**DOI:** 10.1038/s41419-021-04428-7

**Published:** 2021-12-14

**Authors:** Dadong Liu, Wen Sun, Danying Zhang, Zongying Yu, Weiting Qin, Yishu Liu, Kai Zhang, Jiangtao Yin

**Affiliations:** 1 Department of Critical Care Medicine, Affiliated Hospital of Jiangsu University, Zhenjiang, China; 2 Department of Critical Care Medicine, Jurong Hospital Affiliated to Jiangsu University, Zhenjiang, China; 3 Department of Laboratory Medicine, Affiliated People’s Hospital of Jiangsu University, Zhenjiang, China; 4 Department of Electrocardiograph, The No. 4 Affiliated Hospital of Jiangsu University, Zhenjiang, China; 5 State Key Laboratory of Oncogenes and Related Genes, Shanghai Cancer Institute, Shanghai Jiao Tong University, Shanghai, China; 6 Department of Gastrointestinal Surgery, Affiliated Hospital of Jiangsu University, Zhenjiang, China; 7 Department of Otorhinolaryngology and Head and Neck Surgery, Affiliated Hospital of Jiangsu University, Zhenjiang, China

**Keywords:** Bacterial infection, Sepsis

## Abstract

Metabolic reprogramming is a hallmark of neutrophil activation in sepsis. LncRNAs play important roles in manipulating cell metabolism; however, their specific involvement in neutrophil activation in sepsis remains unclear. Here we found that 11 lncRNAs and 105 mRNAs were differentially expressed in three transcriptome datasets (GSE13904, GSE28750, and GSE64457) of gene expression in blood leukocytes and neutrophils of septic patients and healthy volunteers. After Gene Ontology biological process analysis and lncRNA–mRNA pathway network construction, we noticed that GSEC lncRNA and PFKFB3 were co-expressed and associated with enhanced glycolytic metabolism. Our clinical observations confirmed the expression patterns of GSEC lncRNA and PFKFB3 genes in neutrophils in septic patients. Performing in vitro experiments, we found that the expression of GSEC lncRNA and PFKFB3 was increased when neutrophils were treated with inflammatory stimuli. Knockdown and overexpression experiments showed that GSEC lncRNA was essential for mediating PFKFB3 mRNA expression and stability in neutrophil-like dHL-60 cells. In addition, we found that GSEC lncRNA-induced PFKFB3 expression was essential for mediating dHL-60 cell inflammatory cytokine expression. Performing mechanistic experiments, we found that glycolytic metabolism with PFKFB3 involvement supported inflammatory cytokine expression. In summary, our study uncovers a mechanism by which GSEC lncRNA promotes neutrophil inflammatory activation in sepsis by supporting glycolytic metabolism with PFKFB3.

## Introduction

Sepsis is recognized as life-threatening organ dysfunction caused by the dysregulated host response to infection [1]. Despite the recent development of clinical therapeutic interventions, sepsis continues to be a major cause of death resulting from infection [1–3]. The pathogenesis of sepsis has not been well elucidated until now. Current efforts in understanding sepsis have indicated that metabolic reprogramming of innate immune cells plays a vital role in sepsis progression [4–6].

Neutrophils, the most abundant innate immune cells in human blood, reach the affected area of infection early to reduce the number of intruders and direct adaptive immune responses during sepsis [7]. However, excessive inflammatory neutrophil activation may lead to tissue damage and contribute to the development of organ dysfunction [8–10]. Recent studies have indicated that neutrophils undergo metabolic reprogramming to adapt to diverse disease conditions, such as sepsis, diabetes, and atherosclerosis [11–14]. Efforts to understand how neutrophils metabolic status is shifted may provide clues for developing better therapeutics for sepsis.

Several well-executed studies have suggested that glycolysis is the predominant metabolic pathway of neutrophil-mediated pathogen clearance [15–17]. Furthermore, glycolysis inhibition protected mice against enhanced neutrophilic responses, suggesting a direct connection of metabolic modulation during infection and inflammation [18]. 6-Phosphofructo-2-kinase/fructose-2,6-biphosphatase 3 (PFKFB3) is a well-known regulator of glycolysis [19]. By mediating both the synthesis and degradation of fructose-2,6-bisphosphate, PFKFB3 is required for cell proliferation and survival [20–22]. A recent study showed that blockade of the glycolytic activator PFKFB3 in tumor endothelial cells reduced cancer cell invasion, intravasation, and metastasis, and improved the effect of chemotherapy on primary and metastatic tumors [23]. However, little is known about the role of PFKFB3 in regulating neutrophil metabolism and function. Long noncoding RNAs (lncRNAs) are longer than 200 nucleotides and contribute to transcriptional control and posttranscriptional processing functions [24]. By interacting with mate RNA molecules, lncRNAs could potentially participate in modulation of mRNA stability control and translation activation [25–27]. Accumulating evidence has revealed the critical role that lncRNAs play to influence cellular metabolism in cell proliferation, activation, and apoptosis [28–30]. However, whether lncRNAs can manipulate neutrophil metabolic reprogramming in sepsis remains unclear.

In the present study, we found that the expression of G-quadruplex forming sequence-containing lncRNA (GSEC) in neutrophils was significantly increased in septic patients and positively correlated with PFKFB3 mRNA expression. In addition, in vitro studies confirmed that GSEC upregulated PFKFB3 transcription, and that glycolytic metabolism with PFKFB3 involvement further contributed to neutrophil inflammatory factor production. Our findings offer novel insight into the immune disorders in sepsis and may facilitate the exploration of potential therapeutic targets.

## Results

### Identification of differentially expressed lncRNAs and mRNAs in immunocytes of septic patients

Aberrant transcriptional status is a hallmark of immune disorder in sepsis [31]. To identify the differentially expressed genes of immunocytes in septic patients, three sets of gene expression data (GSE64457, GSE28750, and GSE13904) were downloaded from the Gene Expression Omnibus (GEO) database for analysis in this study [32–34]. Total RNA of septic patients and healthy volunteers was independently extracted from blood neutrophils (GSE64457) and blood leukocytes (GSE28750 and GSE13904).

**Fig. 1:**
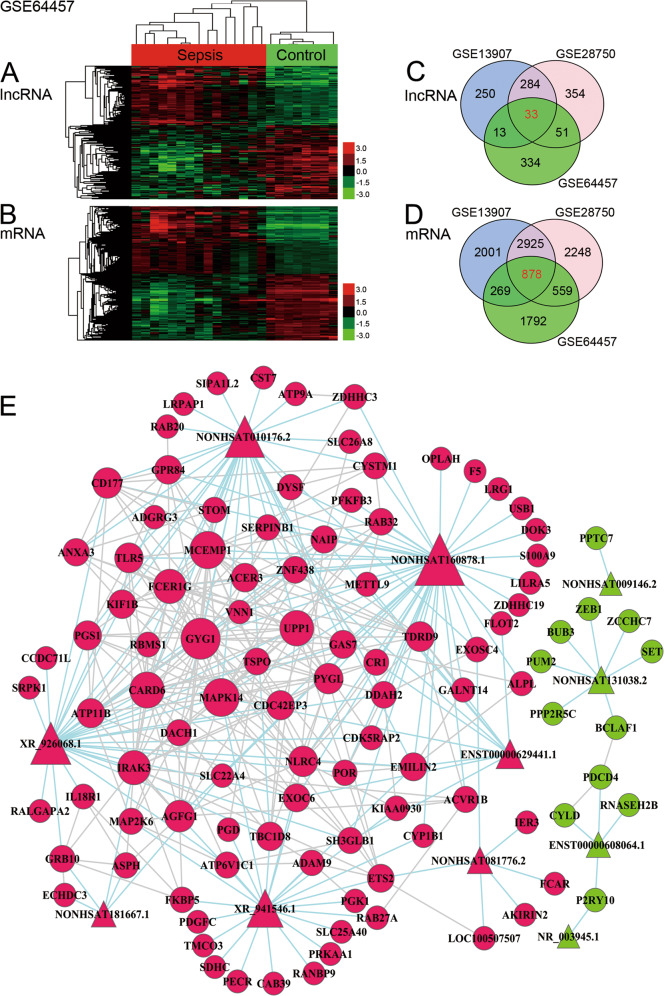
Identification of differentially expressed lncRNAs and mRNAs in the immunocytes of septic patients. **A** Heat map of 431 differentially expressed lncRNAs (including 192 upregulated and 239 downregulated lncRNAs) identified in the microarrays in the GSE64457 dataset. **B** Heat map of 3498 differentially expressed mRNAs (including 1765 upregulated and 1733 downregulated mRNAs) identified in the microarrays in the GSE64457 dataset. **C** Venn plot of the 1319 differentially expressed lncRNAs in the 3 datasets. **D** Venn plot of the 10,672 differentially expressed mRNAs in the 3 datasets. **E** Co-expression network of the significantly differentially expressed lncRNAs and mRNAs. Circles indicate mRNAs and triangles indicate lncRNAs. Red represents upregulated expression and green represents downregulated expression. Baby blue lines indicate the interaction between lncRNAs and mRNAs, and gray lines indicate mRNAs interacting with other mRNAs.

A hierarchical cluster analysis of each dataset was performed to obtain an overview of the expression profile of differentially expressed genes. The heat maps generated from the results showed a distinct regulatory direction and a clear separation between the differentially expressed lncRNAs in the sepsis samples and matched control samples (Fig. 1A and Supplementary Figs. 1A and 2A) and the differentially expressed mRNAs in the sepsis and control samples (Fig. 1B and Supplementary Figs. 1B and 2B). Next, the differentially expressed genes between sepsis samples and matched control samples were analyzed based on the following criteria: fold change *>*1.2 or < −1.2, a false discovery rate (FDR) < 0.05, and a *P*-value *<* 0.05 [35]. We found that 1733 lncRNAs (431 in GSE64457, 722 in GSE28750, and 580 in GSE13904) and 16,181 mRNAs (3498 in GSE64457, 6610 in GSE28750, and 6073 in GSE13904) were differentially expressed in septic patients.

Then, a Venn plot was generated to calculate the number and proportion of the differentially expressed genes in the three datasets. We obtained 33 overlapping lncRNAs (Fig. 1C and Supplementary Table 1), including 21 upregulated and 12 downregulated, and 878 overlapping mRNAs (Fig. 1D and Supplementary Table 2), including 464 upregulated and 414 downregulated. Interestingly, we noticed that, among the upregulated genes, TLR2, TLR5 and interleukin (IL) receptor-associated kinase 3 have been extensively studied in the regulation of inflammation and immune function [36–38]. In addition, metabolism-related genes, including *PFKFB3*, *PRKAA1*, *PYGL*, and *GYG1*, were also upregulated, which indicated their potential roles in mediating immune responses in sepsis [39–41].

### LncRNA–mRNA co-expression analysis and functional annotation

Increasing evidence confirms that lncRNAs play important roles in regulating the expression of protein-coding genes and, therefore, identifying co-expressed protein-coding genes may help in the assessment of lncRNA functions [42]. Therefore, lncRNA–mRNA co-expression analysis was performed to explore the relationship between lncRNAs and mRNAs in sepsis.

We calculated the Pearson’s correlation for each pair of genes and defined the significantly correlated pair as the absolute value of any correlation coefficient greater than 0.75. A total of 8834 differential lncRNA–mRNA pairs (1439 in GSE64457, 5620 in GSE28750, and 1778 in GSE13904) and 4150 differential mRNA–mRNA pairs (718 in GSE64457, 2483 in GSE28750, and 949 in GSE13904) were identified in the 3 datasets. Among these differentially expressed pairs, 372 differential overlapping pairs (including 166 lncRNA–mRNA pairs and 206 mRNA–mRNA pairs) were used to construct a co-expression network (Fig. 1E). From the co-expression network analysis, we obtained 11 lncRNAs (7 upregulated and 4 downregulated) (Supplementary Table 3) and 105 mRNAs (93 upregulated and 12 downregulated) (Supplementary Table 4).

Gene Ontology (GO) biological process analysis was performed based on the 105 co-expressed mRNAs [43]. With the criteria of an FDR < 0.05 and a *P*-value *<* 0.01, we obtained 54 biological processes, including 35 upregulated processes (Supplementary Table 5) and 19 downregulated processes (Supplementary Table 6). Among these processes, 22 upregulated biological processes (enriched with 39 mRNAs) were related to the immune response (Supplementary Fig. 3A and Supplementary Table 7) and metabolic processes (Supplementary Fig. 3B and Supplementary Table 8). Glucose is the basic nutrient in cells that is required to maintain normal survival and function. A growing body of evidence suggests that activated immunocytes generate energy in large part by upregulating aerobic glycolysis [44–46]. In this study, we found that the glucose metabolic process was upregulated, and that six co-expressed mRNAs (GYG1, MAPK14, PFKFB3, PGK1, PRKAA1, and PYGL) were enriched in this process (Supplementary Fig. 3B and Supplementary Table 8). In addition, other significantly enriched biological processes were involved in cell differentiation and gene transcription (Supplementary Fig. 3C and Supplementary Tables 5 and 6).

A Kyoto Encyclopedia of Genes and Genomes (KEGG) pathway analysis was performed to determine the significantly changed pathways in which the co-expressed mRNAs are involved [47]. Based on the criteria of an FDR < 0.05 and a *P*-value < 0.01, we identified 15 upregulated pathways and 1 downregulated pathway in immunocyte dysfunction in septic patients (Supplementary Table 9). Consistent with our findings in the GO biological process analysis, among the upregulated pathways, 11 pathways (enriched with 21 mRNAs) were found to be related to the immune response (Supplementary Fig. 4A and Supplementary Table 10) and metabolic processes (Supplementary Fig. 4B and Supplementary Table 11). The adenosine monophosphate-activated protein kinase pathway, which is extensively involved in cellular energy homeostasis, was also upregulated and associated with three co-expressed mRNAs (CAB39, PFKFB3, and PRKAA1) in sepsis [48]. These results suggested that immunocytes of septic patients undergo metabolic reprogramming.

### LncRNA–mRNA pathway network analysis

**Fig. 2:**
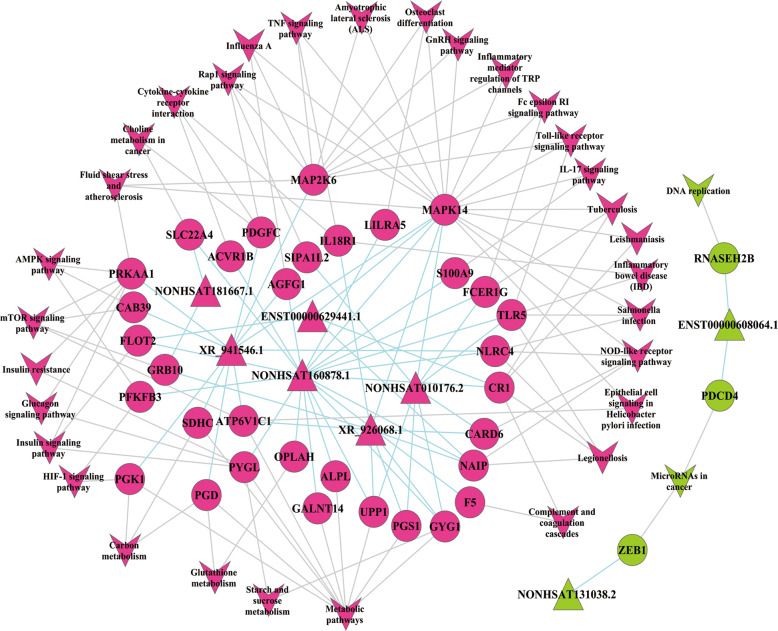
Pathway network analysis of the significantly differentially expressed lncRNAs and mRNAs. The role of each pathway in the network was measured by counting its connections to upstream and downstream pathways. A pathway with a high degree value likely plays an important role in the signaling network. Circles indicate mRNAs and triangles indicate lncRNAs. Red represents upregulated pathways and green represents downregulated pathways. Baby blue lines indicate the interaction between lncRNAs and mRNAs, and gray lines indicate interactions between pathways.

To explore the specific lncRNA–mRNA interaction in sepsis-related metabolism reprogramming, lncRNA–mRNA pathway networks were constructed. The results showed that 6 upregulated lncRNAs and 27 associated upregulated mRNAs were involved in 15 upregulated pathways (FDR < 0.05 and *P* < 0.01) (Fig. 2). Among these lncRNAs, the NONHSAT160878.1 lncRNA was associated with the most mRNAs (degree = 20). It was involved in glucose metabolism by associating with four glucose metabolism-related mRNAs (GYG1, MAPK14, PFKFB3, and PYGL).

The lncRNA NONHSAT160878.1 is located on chromosome 11 (126340958–126355587) and was identified as a GSEC. A previous study showed that GSEC can modulate colon cancer progression by promoting cell migration [49]. However, the biological role of GSEC in sepsis remains unclear. PFKFB3 is a well-known regulator of glycolytic metabolism and plays an important role in cell survival and activation [20–22]. Based on our analysis, GSEC and PFKFB3 were co-expressed in leukocytes of septic patients and associated with neutrophil glycolysis. However, whether a causal relationship exists between GSEC and the PFKFB3 genes remains to be determined.

### Expression and correlation of GSEC and PFKFB3 genes in septic neutrophils

**Fig. 3:**
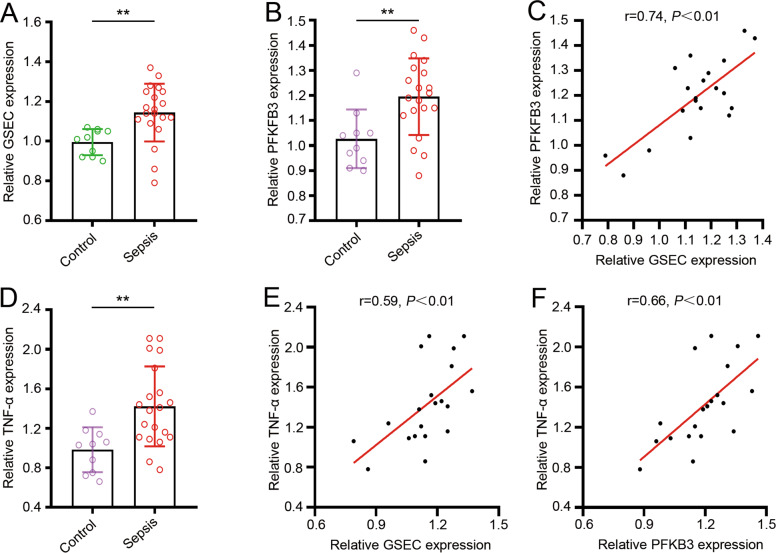
Expression and correlation of GSEC, PFKFB3, and inflammatory cytokine genes in septic neutrophils. **A** Expression of GSEC in septic neutrophils. **B** Expression of PFKFB3 in septic neutrophils. **C** Correlation of GSEC and PFKFB3 genes in septic neutrophils. **D** Expression of TNF-α in septic neutrophils. **E** Correlation of GSEC and TNF-α in septic neutrophils. **F** Correlation of PFKFB3 and TNF-α in septic neutrophils. For each group: septic patients, *n* = 20; healthy volunteers, *n* = 10, three independent experiments. Statistics were calculated using Student’s *t*-test (**A**, **B**, **D**) or Pearson’s analysis (**C**, **E**, **F**).

Neutrophils are the most abundant innate immunocytes and the first mediator to enter the affected area during infection [7]. However, in sepsis, overactivation of neutrophils contributes to systemic inflammation and is the main cause of organ dysfunction. To investigate whether neutrophil inflammatory activation is related to GSEC and/or PFKFB3 mRNA, primary neutrophils were obtained from the blood of septic patients and healthy volunteers, and the expression of GSEC and PFKFB3 was determined. The results showed that neutrophils in septic patients had significantly higher expression of GSEC and PFKFB3 than those in healthy volunteers (Fig. 3A, B). Pearson’s correlation analysis showed that GSEC was positively correlated with PFKFB3 mRNA (*r* = 0.74, *P* < 0.01) (Fig. 3C), which was consistent with the results obtained from the analysis of the three GEO datasets (Supplementary Fig. 5). In addition, we found that the mRNA expression of inflammatory cytokine tumor necrosis factor (TNF)-α was upregulated in septic neutrophils (Fig. 3D). Another correlation analysis indicated that TNF-α mRNA expression was positively correlated with GSEC (Fig. 3E) and PFKFB3 (Fig. 3F). Similar results were found for IL-1β (Supplementary Fig. 6) and IL-6 (Supplementary Fig. 7) expression in septic neutrophils. These results suggest the potential interaction between GSEC and PFKFB3 in neutrophil inflammation activation during sepsis.

### GSEC is essential for PFKFB3 expression in inflammatory neutrophils

**Fig. 4:**
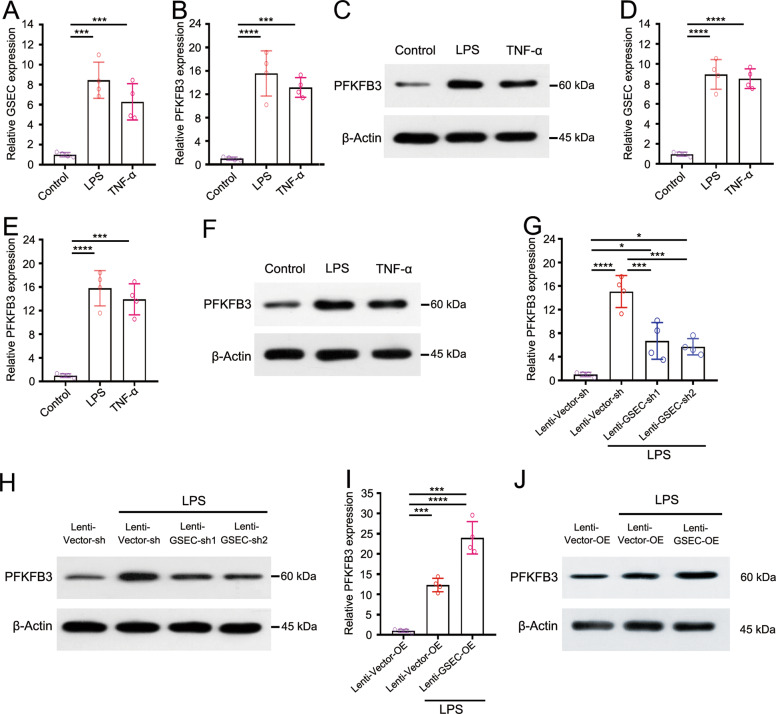
GSEC is essential for PFKFB3 expression in inflammatory neutrophils. **A** The expression level of GSEC in primary human neutrophils. **B** The expression level of PFKFB3 in primary human neutrophils. **C** Western blot analysis of PFKFB3 in primary human neutrophils. **D** The expression level of the GSEC in dHL-60 cells. **E** The expression level of PFKFB3 in dHL-60 cells. **F** Western blot analysis of PFKFB3 in dHL-60 cells. **G** The level of PFKFB3 expression in GSEC-knockdown dHL-60 cells. **H** Western blot analysis for PFKFB3 in GSEC-knockdown dHL-60 cells. **I** The level of PFKFB3 expression in GSEC-overexpressing dHL-60 cells. **J** Western blot analysis of PFKFB3 in GSEC-overexpressing dHL-60 cells. Neutrophils and dHL-60 cells were treated LPS or TNF-α for 12 h. Statistics were calculated using one-way ANOVA with Tukey’s post hoc tests. *P < 0.05, **P < 0.01, ***P < 0.001, ****P < 0.0001.

We examined whether the sepsis-associated inflammatory stimuli lipopolysaccharide (LPS) and TNF-α regulate the expression of GSEC and PFKFB3 mRNA in neutrophils in vitro. We found that LPS and TNF-α stimulation markedly increased the expression levels of the GSEC and PFKFB3 in primary human neutrophils compared with that in the control group (Fig. 4A–C and Supplementary Figs. 8A and 9A, B). Similar results were found in neutrophil-like differentiated HL-60 (dHL-60) cells (Fig. 4D–F and Supplementary Figs. 8B and 9C, D), in vitro neutrophil models extensively used by our group [50] and other researchers [51].

Recent studies have indicated the essential role of lncRNAs in regulating mRNA expression [52]. As primary neutrophils cannot be genetically manipulated, dHL-60 cells were used to determine whether GSEC can influence the expression of PFKFB3. We used lentiviral vectors to generate dHL-60 cells with stable GSEC knockdown or overexpression (OE), and an empty vector was used as the control. We found that LPS stimulation significantly increased the expression of PFKFB3 in the empty vector-transfected dHL-60 cells. However, in the GSEC-knockdown cells, the expression of PFKFB3 was significantly reduced (Fig. 4G, H and Supplementary Figs. 8C and 9E, F). In contrast, LPS-enhanced PFKFB3 expression was further increased in the GSEC-overexpressing dHL-60 cells (Fig. 4I, J and Supplementary Figs. 8D and 9G, H). However, these reductions were not observed in GSEC-knockdown dHL-60 cells without LPS stimulation (Supplementary Fig. 10).

### GSEC was essential for enhancing PFKFB3 mRNA stability

**Fig. 5:**
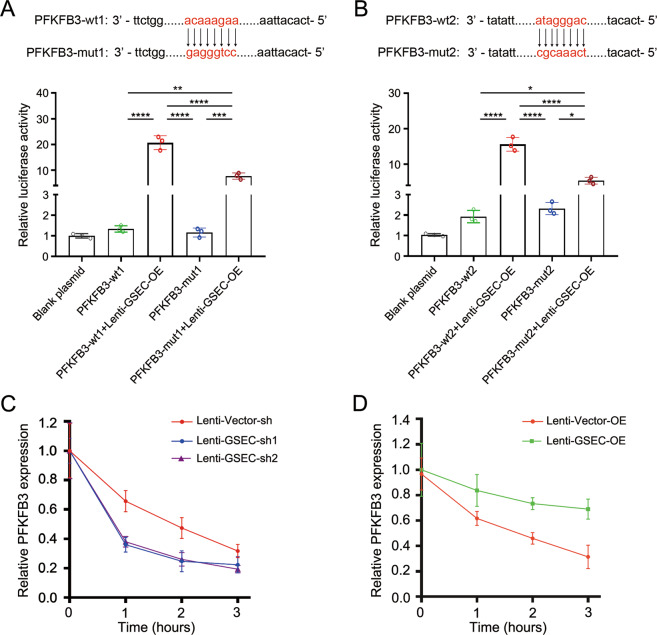
GSEC was essential for enhancing PFKFB3 mRNA stability. **A** GSEC positively regulates PFKFB3 transcription through the active site (acaaagaa) of PFKFB3. **B** GSEC positively regulates PFKFB3 transcription through the active site (ataggac) of PFKFB3. **C** The stability of PFKFB3 expression in GSEC-knockdown dHL-60 cells. **D** The stability of PFKFB3 expression in GSEC-overexpressing dHL-60 cells. The dHL-60 cells were treated LPS for 12 h. Statistics were calculated using one-way ANOVA with Tukey’s post tests. *P < 0.05, **P < 0.01, ***P < 0.001, ****P < 0.0001.

Dual-luciferase reporter assay was used to investigate whether GSEC lncRNA could regulate PFKFB3 mRNA transcription. Results indicated that GSEC lncRNA can positively regulate PFKFB3 transcription through two different regions of PFKFB3 (Fig. 5A, B). A previous study revealed that lncRNAs can regulate mRNA expression by influencing mRNA stability [53]. Thus, we next sought to determine whether the GSEC can influence the stability of PFKFB3 mRNA. Using actinomycin D to block transcription, we observed that PFKFB3 mRNA expression was gradually reduced in LPS-stimulated dHL-60 cells (Fig. 5C, D). The GSEC-knockdown cells showed a faster rate of PFKFB3 mRNA decay than the control cells (Fig. 5C), whereas the GSEC-overexpressing cells showed an attenuated rate of PFKFB3 mRNA decay (Fig. 5D). In addition, we found that lentiviral vector stimulation did not influence the stability of PFKFB3 mRNA, and that GSEC OE did not reduce the rate of GSEC lncRNA decay in dHL-60 cells (Supplementary Fig. 11). These data indicated that GSEC was essential for supporting PFKFB3 expression in inflammatory dHL-60 cells by enhancing PFKFB3 mRNA stability.

### GSEC/PFKFB3-supported glycolytic metabolism enhances dHL-60 cell inflammatory factor expression

**Fig. 6:**
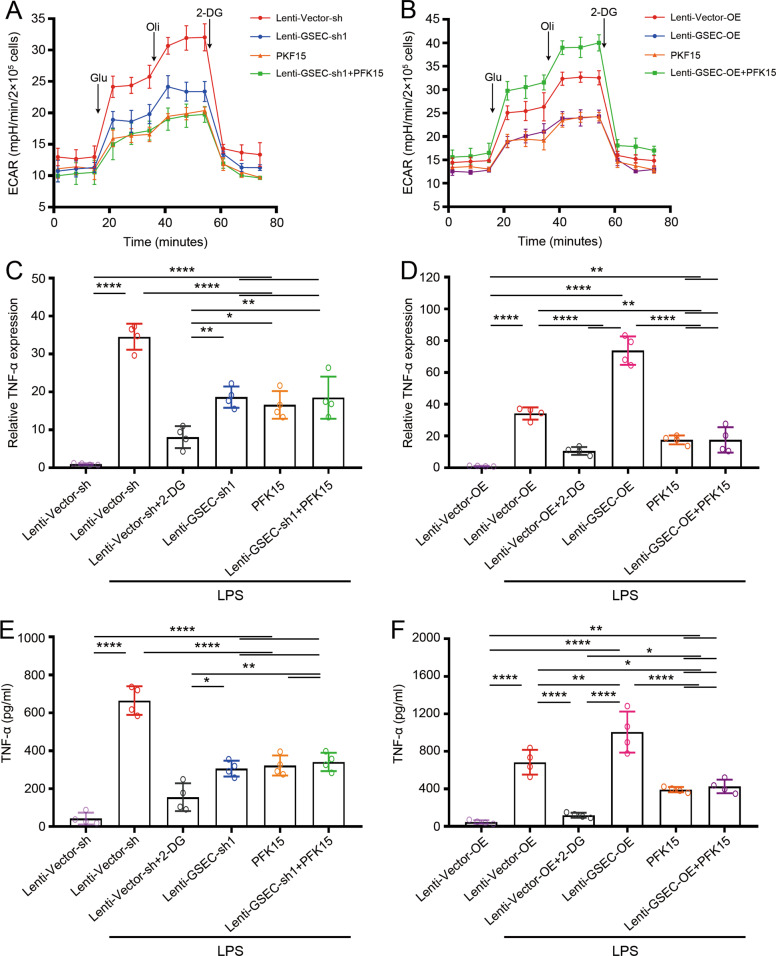
GSEC/PFKFB3-supported glycolytic metabolism enhances dHL-60 cell inflammatory factor expression. **A** The ECAR was detected in GSEC-knockdown dHL-60 cells. **B** The ECAR was detected in GSEC-overexpressing dHL-60 cells. **C** The level of TNF-α mRNA expression was detected in GSEC-knockdown dHL-60 cells. **D** The level of TNF-α mRNA expression was detected in GSEC-overexpressing dHL-60 cells. **E** The level of TNF-α protein was detected in GSEC-knockdown dHL-60 cells. **F** The level of TNF-α protein was detected in GSEC-overexpressing dHL-60 cells. For all experiments: *n* = 4 per group, three independent experiments. Neutrophils and dHL-60 cells were treated LPS for 12 h. *P < 0.05, **P < 0.01, ***P < 0.001, ***P < 0.0001, ^ns^*P* indicates no statistical significance. Glu, glucose; Oli, oligomycin; 2-DG, 2-deoxyglucose.

To investigate whether glycolytic metabolism was regulated by the effects of GSEC on PFKFB3 expression, the extracellular acid ratio (ECAR) was measured in dHL-60 cells. The lentiviral vectors did not affect the ECAR in LPS-stimulated dHL-60 cells (Supplementary Fig. 12). After LPS stimulation, the GSEC-knockdown dHL-60 cells showed a lower ECAR than the control dHL-60 cells, whereas a higher ECAR was observed in the GSEC-overexpressing dHL-60 cells (Fig. 6A, B). This result indicated that GSEC was essential for supporting neutrophil glycolysis. To further investigate whether GSEC reprograms the glycolytic metabolism in neutrophils through PFKFB3 mRNA. PFK15, a small molecule inhibitor of PFKFB3, was used to inhibit PFKFB3 enzyme activity in dHL-60 cells. The results showed that PFK15 markedly reduced the ECAR in the dHL-60 cells. Similar ECARs were detected in the PFK15-treated GSEC-knockdown and GSEC-overexpressing dHL-60 cells. These results indicated that PFKFB3 expression regulated by GSEC was involved in glycolytic reprogramming in dHL-60 cells.

Next, we explored whether inflammatory cytokine expression is regulated by GSEC through glycolysis involving PFKFB3 expression. First, we found that lentiviral vectors did not affect TNF-α expression in dHL-60 cells (Supplementary Fig. 13A). After LPS stimulation, we found that TNF-α expression was markedly upregulated compared with that in unstimulated cells. Then, TNF-α expression was measured in GSEC-knockdown and GSEC-overexpressing dHL-60 cells. We observed that GSEC-knockdown dHL-60 cells expressed a lower level of TNF-α mRNA than LPS-stimulated vehicle cells and TNF-α mRNA expression level in PFK15-inhibited cells was reduced to a level similar to that of the GSEC-knockdown dHL-60 cells (Fig. 6C). PFK15 failed to further reduce the expression of TNF-α mRNA in the GSEC-knockdown cells. In contrast to our findings with GSEC-knockdown cells, we observed that TNF-α mRNA expression was higher in GSEC-overexpressing cells (Fig. 6D). PFK15 inhibited the expression of TNF-α mRNA in vector cells and GSEC-overexpressing cells. 2-Deoxy-d-glucose (2-DG), an inhibitor of hexokinase II, was used to block glycolysis in dHL-60 cells. The results showed that 2-DG significantly inhibited TNF-α mRNA expression (Fig. 6C, D), which indicated that glycolysis supported inflammatory cytokine mRNA expression in activated dHL-60 cells. Furthermore, we found that 2-DG further reduced the mRNA expression of TNF-α in the GSEC-knockdown and GSEC-overexpressing cells stimulated by LPS (Fig. 6C, D and Supplementary Fig. 14A, B). The level of TNF-α protein was similar, as determined by enzyme-linked immunosorbent assay (ELISA) (Fig. 6E, F and Supplementary Figs. 13B and 14C, D). Furthermore, we wondered whether inflammatory IL-1β and IL-6 expression is regulated by GSEC through glycolytic metabolism involving PFKFB3. The expression of IL-1β (Supplementary Figs. 15 and 16) and IL-6 (Supplementary Figs. 17 and 18) was similar to that of TNF-α. These results suggested that GSEC-elevated inflammatory cytokine expression is PFKFB3 dependent. Together, our findings indicated that GSEC/PFKFB3-supported glycolytic metabolism promoted inflammatory cytokine expression in dHL-60 cells.

## Discussion

The proinflammatory response in the acute phase of sepsis eliminates invading pathogens and involves immunocyte activation and subsequent production of cytokines such as TNF-α, IL-1β, and IL-6 [54]. However, a hyperactive proinflammatory response may exert detrimental effects on the host by eliciting high fevers, hypotension, and organ failure [55]. Therefore, to develop effective therapeutic strategies for improving patient prognosis after sepsis, it is crucial to understand the precise molecular mechanisms of sepsis-induced immunocyte activation. Recent studies have shown that numerous metabolic changes in immunocytes appear to be vital for immune system dysfunction and sepsis progression [5, 56–58]. A typical metabolic change is observed in the hyperinflammatory phase of sepsis and is accompanied by a metabolic switch from oxidative phosphorylation to glycolytic metabolism in many immunocytes [58–61]. Our bioinformatics analyses of three independent GEO datasets confirmed that glycolytic metabolism and associated pathways were activated in blood immunocytes. Because of the crucial role of glycolysis in immunocyte proinflammatory activation [62], we speculate that further investigation into the metabolism reprogramming mechanism in immunocyte during sepsis may facilitate the identification of potential therapeutic targets.

Glycolytic metabolism (also termed glycolysis) begins with the uptake of extracellular glucose and subsequent processing of glucose in the cytosol to eventually yield adenosine triphosphate (ATP) and numerous other products [16]. Previous studies have shown that enhanced glycolysis enables immunocytes to generate sufficient ATP and biosynthetic intermediates to carry out its particular effector functions, including antigen presentation and inflammatory cytokine production [58, 63–65]. In addition, recent advances experiments based on animal models of sepsis have revealed that decreases in glycolysis can inhibit the release of proinflammatory cytokines and thus improve the survival outcomes of the animal models [66]. Consistent with these previous studies, our results confirmed that LPS stimulation increased the level of ECAR, a key indicator of glycolysis, in dHL-60 cells. Therefore, investigating the mechanism of sepsis-induced glycolysis may provide a scientific basis for sepsis treatment.

Other studies into the mechanism have revealed that genes encoding key enzymes associated with glycolysis are upregulated in human leukocytes during the initial hyperinflammation induced by endotoxin [67]. In agreement with this study on leukocytes, our results showed that during the acute phase of sepsis, the expression level of PFKFB3 mRNA was upregulated in primary human neutrophils. PFKFB3 is a key regulatory glycolytic enzyme involved in glucose decomposition into ATP and has dual kinase and phosphatase activities [19]. PFKFB3 kinase activity is known to increase the rate of glycolysis and promote the proliferation, migration, invasion, and growth of tumor cells [68–70]. However, recent studies have revealed another role for PFKFB3: PFKFB3-mediated glycolysis is involved in the regulation of the inflammatory response. Jiang et al. [71] found that PFKFB3 promotes the antiviral capacity of macrophages by metabolically supporting the uptake and elimination of virus-infected cells. Zhang et al. [72] demonstrated that TNF-α stimulation increased endothelial PFKFB3 expression and PFKFB3 knockdown almost completely blocked the TNF-α-induced release of proinflammatory cytokines. Similar to these studies, our study showed that both TNF-α and LPS stimulation can significantly increase the expression of PFKFB3 in primary human neutrophils. In addition, we found that the PFKFB3 expression is increased in the neutrophils of septic patients. Some recently published studies have shown that blocking PFKFB3 protects mice from acute lung injury by suppressing hyperinflammation [73–75]. Consistent with these studies, our study also revealed that inhibiting PFKFB3 enzyme activity with PFK15 markedly reduced the rate of glycolysis and the expression of TNF-α, IL-1β, and IL-6 mRNAs and protein in dHL-60 cells. All of these results demonstrate that the glycolytic enzyme PFKFB3 plays a critical role in sepsis-induced neutrophil activation.

In addition to known mRNAs, lncRNAs are thought to be involved in glycolytic reprogramming [29, 76–78]. LncRNAs are widely expressed during immunocyte generation, differentiation, and activation, and they can control important aspects of immunity [79]. A few lncRNAs have been implicated in immunocyte glycolysis regulation, but the underlying mechanisms remain poorly understood. Liu et al. [80] found that the lncRNA AGPG plays a pivotal role in glycolysis in esophageal squamous cell carcinoma by directly binding and regulating PFKFB3. In agreement with this study, our results showed that the expression level of GSEC was significantly increased in sepsis-induced neutrophils and it influenced glycolysis in dHL-60 cells by directly enhancing PFKFB3 mRNA transcription and translation. We also verified that this GSEC regulates the transcription of PFKFB3 through different regions of PFKFB3. Considering the proinflammatory role of lncRNAs, we investigated the expression of inflammatory factors in dHL-60 cells exposed to LPS. We found that GSEC/PFKFB3-supported glycolytic metabolism enhances dHL-60 cell inflammatory factor expression.

In conclusion, our study showed that GSEC plays a pivotal role in promoting glycolysis in neutrophils by enhancing PFKFB3 transcription and translation, facilitating neutrophil inflammatory factors production during the acute phase of sepsis (Supplementary Fig. 19). Therefore, our study suggested that GSEC-regulated PFKFB3-mediated glycolytic reprogramming is a potential sepsis therapeutic target. Notably, the method we used to isolate human neutrophils did not eliminate eosinophils. However, as the eosinophil proportion was found to be <1% of the cell population analyzed, we suggested that this proportion might have no significant influence on our main findings.

## Materials and methods

### Materials

LPS (*Escherichia coli* O55:B5), dimethylsulfoxide (DMSO), fetal bovine serum (FBS), and TNF-α were obtained from Sigma-Aldrich (St. Louis, MO, USA). Hanks’ balanced salt solution (HBSS) and RPMI-1640 medium were obtained from Thermo Scientific (Waltham, MA, USA). TRIzol reagent and a RNeasy kit were obtained from Qiagen (Dusseldorf, NRW, Germany). ELISA kits for IL-1β, TNF-α, and IL-6 detection were obtained from Qiaoyi (Shanghai, China). Rabbit anti-PFKFB3 monoclonal antibody and rabbit anti-β-actin monoclonal antibody were obtained from CST (Boston, MA, USA). GSEC-knockdown and OE lentivirus vectors were constructed by GeneChem (Shanghai, China). A NanoDrop ND-1000 spectrophotometer was obtained from Thermo Fisher Scientific (Waltham, MA, USA). A Seahorse XF96 Flux Analyzer was obtained from Agilent Technologies (Palo Alto, CA, USA). SYBR Premix Ex Taq was obtained from Roche Life Science (Basel, KB, Switzerland).

### GEO database retrieval

We systematically retrieved public microarray datasets by using the key word “sepsis” in the GEO database (https://www.ncbi.nlm.nih.gov/geo/). Datasets were selected according to the following criteria: the data referred to (1) human genes; (2) studies with case control(s); (3) neutrophils (at least those in whole blood) obtained from participants and used for gene expression analysis; and (4) details of gene expression.

After careful filtering, three sets of gene expression data (GSE13904, GSE28750, and GSE64457) were included in this study [32–34]. We included data of 50 leukocyte samples (including 18 samples from control subjects and 32 samples from septic patients (day 1) from the GSE13904 dataset, 30 leukocyte samples (including 20 samples from control subjects and 10 samples from septic patients) from the GSE28750 dataset, and 23 neutrophil samples (including 8 samples from control subjects and 15 samples from septic patients) from the GSE64457 dataset.

### Identification of annotated and differentially expressed genes

Raw CEL files of the three datasets were downloaded from the GEO database and then preprocessed (background correction, quantile normalization, and log2 transformation) using the robust multichip average method with the R package “affy” [81]. Next, hybridization probes were mapped to genes (Entrez Gene IDs) according to the platform table (GPL570). In addition, to profile the gene expression in the data, the expression levels of the genes in the downloaded datasets were compared with those in the NCBI RefSeq transcript (mRNA and lncRNA), Ensembl (lncRNA), and NONCODE 2016 (lncRNA) databases using the BLAST program. The workflow of probe set annotation is summarized in Supplementary Fig. 20. The differentially expressed genes in the septic patients were identified by hierarchical cluster analyses and Venn plots.

### Co-expression network construction and enrichment analyses

A gene co-expression network analysis was performed to elucidate the relationship between lncRNAs and mRNAs, and identify the key genes in septic patients by using the R package “WGCNA” [82]. A co-expression network was constructed by using Cytoscape 3.4.0 software [83]. To investigate the biological function and signaling pathways of mRNAs, DAVID (http://david.ncifcrf.gov) was used for GO annotation and KEGG pathway enrichment of co-expressed mRNAs [43, 47, 84]. A lncRNA–mRNA pathway network was created based on the results of the lncRNA–mRNA co-expression network and pathway analyses by using Cytoscape 3.4.0 software [83].

### Clinical settings

A prospective case–control study was performed to evaluate the expression of genes in neutrophils in septic patients. Septic patients who were admitted to the intensive care unit (ICU) of the Affiliated Hospital of Jiangsu University between October 2018 and December 2019 were recruited. The inclusion criteria for septic patient inclusion were as follows: (1) age ≥ 18 years; (2) clinical evidence of infection with sequential organ failure assessment (SOFA) score ≥ 2. The exclusion criteria were as follows: (1) age > 80 years; (2) patients whose immune system was suppressed (such as individuals with HIV, autoimmune disease, and/or cancer); (3) patients with hematopoietic disease; and (4) patients who received drugs within the last 2 weeks that might affect neutrophil function. A total of 20 septic patients were included in the present study.

Data including age, sex, SOFA score, Acute Physiology and Chronic Health Evaluation II score, absolute lymphocyte count, absolute neutrophil count, primary site of infection, pathogenic bacteria causing infection, and 28-day mortality were collected. Blood from septic patients was collected at admission to the ICU and before treatment of the infection. Ten healthy volunteers who underwent routine physical examinations in the hospital were recruited via clinical history and laboratory studies to serve as controls. There was no significant difference in the basic data (except absolute neutrophil count) between the two groups (Supplementary Table 12).

After written informed consent was obtained, blood specimens were extracted from the cubital veins of septic patients and healthy drug-free donors. This study was approved by the Medical Ethical Committee of Jiangsu University.

### Human neutrophil isolation and stimulation

Human neutrophils were isolated from human peripheral blood byFicoll/Hypaque centrifugation as previously described [50]. Briefly, human blood was collected and mixed with an equal volume of 3% dextran. The mixed blood was incubated for 30 min at room temperature and the supernatant was centrifuged with Ficoll/Hypaque for 30 min. Then, the neutrophil-containing pellet was resuspended in sterile ddH_2_O water to facilitate erythrocyte lysis. Finally, the neutrophils were washed with HBSS and resuspended in RPMI-1640 with 1% heat-inactivated FBS. The purity (>97%) of the neutrophils was measured by flow cytometry and adjusted on the basis of CD66b and Siglec-8 co-staining (Supplementary Fig. 21) [85–87]. The isolated neutrophils in RMPI-1640 culture medium were stimulated with LPS (100 ng/mL, Sigma-Aldrich) and TNF-α (10 ng/mL, Sigma-Aldrich) for 12 h to mimic the activation of neutrophils in sepsis.

### Generation and differentiation of HL-60 cell lines with GSEC knocked down or overexpressed

GSEC-knockdown and OE lentiviral vectors were constructed by GeneChem (Shanghai, China). Briefly, 2 target sequences (5′-GGTCACAACAGTACAAAGA-3′ and 5′-CCAACTATGCCATGGTCTT-3′) for GSEC were cloned into GV493 lentivirus vectors to construct knockdown lentiviral vectors of GSEC, and they were named lenti-GSEC-sh1 and lenti-GSEC-sh2 (Supplementary Fig. 22A and Supplementary Table 13), and a target sequence for GSEC (NONHSAT160878.1) was cloned into a GV367 lentiviral vector to construct OE lentiviral vectors of GSEC, named lenti-GSEC-OE (Supplementary Fig. 22B and Supplementary Table 13). Stable knockdown and OE cell lines were generated as previously described [51]. Briefly, HL-60 cells were transduced with lenti-GSEC-short hairpin RNA (shRNA) virus, lenti-GSEC-OE virus, or control lentiviral vectors, and cultured in RPMI-1640 medium supplemented with polybrene (5 μg/mL). After 72 h of transduction, puromycin (5 μg/mL) was added to the culture medium for stable cell line selection. GSEC expression was determined by quantitative reverse-transcription PCR (qRT-PCR). The results showed that GSEC expression was significantly reduced in the lenti-GSEC-shRNA-transfected cells (Supplementary Fig. 23A) and increased in the lenti-GSEC-OE-transfected cells (Supplementary Fig. 23B). The lentiviral vectors did not affect GSEC expression. We also found that the lentiviral vectors did not affect the expression of PFKFB3 mRNA in dHL-60 cells with or without LPS stimulation (Supplementary Fig. 24).

To induce the differentiation of neutrophil-like HL-60 (dHL-60) cells, HL-60 cells were cultured in the presence of 1.25% DMSO for 6 days. The dHL-60 cells were then collected and stimulated with LPS (1 μg/mL) for 12 h.

### Quantitative reverse-transcription PCR

qRT-PCR was performed to measure gene (GSEC, PFKFB3, TNF-α, IL-1β, and IL-6) expression as previously described [88]. Total RNA in neutrophils was extracted with TRIzol reagent (Qiagen) and a RNeasy kit (Qiagen) according to the manufacturer’s instructions. RNA purity was determined by using a NanoDrop ND-1000 spectrophotometer (Thermo Fisher Scientific), and RNA integrity was evaluated by standard denaturing agarose gel electrophoresis. Then, RT-qPCR was performed using one step SYBR Premix Ex Taq (Roche) according to the manufacturer’s protocol. After PCR amplification, the comparative Ct method was used for relative quantification of gene expression, which was normalized to β-actin expression. The following primers were used: GSEC sense primer 5′-GAGTTCATTTGCTCTCTCTGGCAC-3′ and antisense primer 5′-AAGAGGAGGCCTGATGGGGATA-3′; PFKFB3 sense primer 5′-ATTGCGGTTTTCGATGCCAC-3′ and antisense primer 5′-GCCACAACTGTAGGGTCGT-3′; TNF-α sense primer 5′-CCTCTCTCTAATCAGCCCTCTG-3′ and antisense primer 5′-GAGGACCTGGGAGTAGATGAG-3′; IL-1β sense primer 5′-AGCTACGAATCTCCGACCAC-3′ and antisense primer 5′-CGTTATCCCATGTGTCGAAGAA-3′; and IL-6 sense primer 5′-CACAGACAGCCACTCACC-3′ and antisense primer 5′-AGTGCCTCTTTGCTGCTTTC-3′.

### Dual-luciferase reporter assays

Dual-luciferase reporter assays were used to demonstrate whether GSEC lncRNA can regulate the transcription of PFKFB3 in dHL-60 cells [89]. First, the PFKFB3 plasmids were constructed by GenePharma for use in dual-luciferase reporter assays (Shanghai, China). Briefly, two wild-type PFKFB3 genes and corresponding mutants were cloned into PGL3B vectors to construct luciferase reporter PFKFB3 plasmids, named PFKFB3-wt1, PFKFB3-mut1, PFKFB3-wt2, and PFKFB3-mut2. Then, dHL-60 cells (1.2 × 10^5^) were grown in 96-well plates and co-transfected with the blank plasmids PFKFB3-wt1/2 (with or without lenti-GSEC-OE) and PFKFB3-mut1/2 (with or without lenti-GSEC-OE). Firefly and *Renilla* luciferase activities were measured 48 h after transfection using a Dual-Glo Luciferase Assay System (Promega, WI, USA) following the manufacturer’s protocol. The firefly luciferase activity was normalized to the *Renilla* luciferase activity.

### PFKFB3 mRNA stability assay

PFKFB3 mRNA stability was determined by qRT-PCR as previously described [88]. Briefly, GSEC expression was knocked down by infection with lenti-GSEC-shRNA. The cells were collected and stimulated with LPS (1 μg/mL) for 12 h to mimic activated neutrophils in sepsis. Then, actinomycin D (5 μg/mL) was added to the cell medium to block de novo RNA synthesis. Total RNA was collected at 0, 1, 2, and 3 h following the addition of actinomycin D. PFKFB3 mRNA expression was measured by qRT-PCR. The PFKFB3 mRNA half-life was determined by comparing to the mRNA level before and after actinomycin D was added.

### Glycolysis assay

The ECAR was measured using a Seahorse XF96 Flux Analyzer (Agilent) according to the manufacturer’s instructions [90]. Briefly, cells were stimulated with LPS (1 μg/mL) for 12 h and then collected. For the ECAR determination, 1 μmol/L oligomycin, 0.5 μmol/L carbonyl cyanide p-trifluoromethoxyphenylhydrazone, and 0.5 μmol/L rotenone plus 0.1 μmol/L antimycin A were injected into the wells. The ECAR were measured in 2 min intervals.

### Inflammatory cytokines

The levels of inflammatory cytokines (TNF-α, IL-1β, and IL-6) were determined by ELISA kits according to the manufacturer’s instructions. Briefly, the supernatant of LPS (1 μg/mL, for 12 h)-stimulated dHL-60 cells was added to a 96-well microtiter plate that contained a specific immobilized antibody that could bind TNF-α, IL-1β, or IL-6 in the aliquot. After unbound substances and antibodies were removed with several washings and when color development was stopped by sulfuric acid, the optical density was determined at 540 nm in an ELISA plate reader. The results were calculated on a standard curve concentration and multiplied by the dilution factor.

### Western blot analysis

Both neutrophils and dHL-60 cells were processed with the addition of RIPA buffer containing a protease inhibitor cocktail. For the detection of target proteins, the cell lysates were subjected to SDS electrophoresis on 10% polyacrylamide gels and transferred to nitrocellulose membranes. Following incubation with rabbit anti-PFKFB3 monoclonal antibody (CST) at 4 °C overnight, the membrane was incubated with the secondary antibody for 1 h at room temperature. Then, the band was visualized with an enhanced chemiluminescence (ECL) reagent and Hyper film ECL as described by the manufacturer.

### Statistical analysis

Our data were statistically analyzed with GraphPad Prism software (version 8). Continuous data with a normal distribution are presented as the means ± SD. Statistical analysis of two groups was performed with Student’s *t*-tests. Categorical data are presented as number (%). The statistical analysis of categorical data was performed with *χ*^2^-tests. Correlations were assessed by Pearson’s analysis. An *α*-value of *P* < 0.05 was considered statistically significant.

## Supplementary information

Reproducibility checklist

Supplementary Figures

Supplementary Table 1

Supplementary Table 2

Supplementary Table 3

Supplementary Table 4

Supplementary Table 5

Supplementary Table 6

Supplementary Table 7

Supplementary Table 8

Supplementary Table 9

Supplementary Table 10

Supplementary Table 11

Supplementary Table 12

Supplementary Table 13

## Data Availability

All datasets generated for this study are included in the article.
