# Supplementary material for: Long noncoding RNA GSEC promotes neutrophil inflammatory activation by supporting PFKFB3-involved glycolytic metabolism in sepsis
Source: Cell Death Dis. 2021 Dec 14;12(12):1157. doi: 10.1038/s41419-021-04428-7 (PMC8671582; doi:10.1038/s41419-021-04428-7)
Supplement: Supplementary file 2 — Supplementary Figures [file 41419_2021_4428_MOESM2_ESM.pdf]

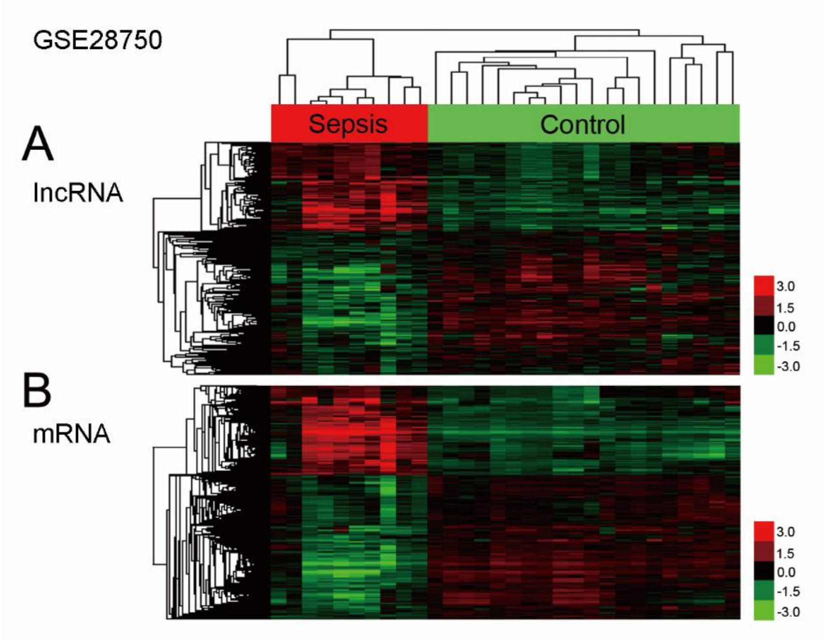

Supplementary Fig. 1 Identification of differentially expressed lncRNAs and mRNAs in immunocytes of septic patients. A Heat map of 722 differentially expressed lncRNAs (including 277 upregulated and 445 downregulated lncRNAs) identified in the microarrays in the GSE28750 dataset. **B** Heat map of 6,610 differentially expressed mRNAs (including 2,530 upregulated and 4,080 downregulated mRNAs) identified in the microarrays in the GSE28750 dataset.

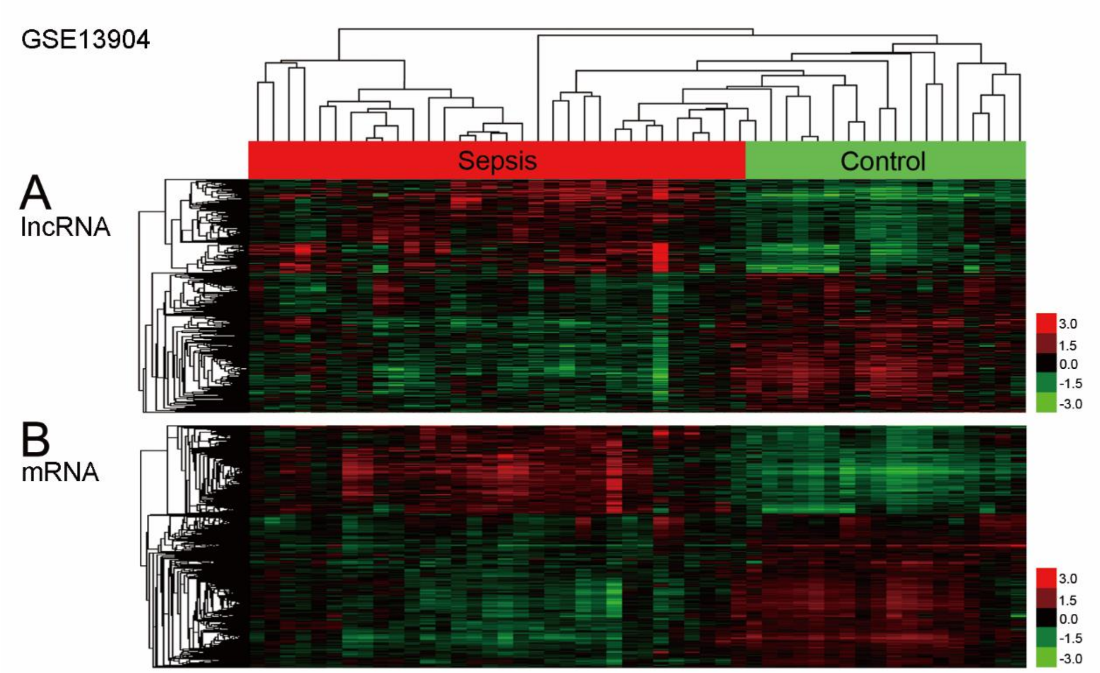

Supplementary Fig. 2 Identification of differentially expressed lncRNAs and mRNAs in immunocytes of septic patients. **A** Heat map of 580 differentially expressed lncRNAs (233 upregulated and 347 downregulated) identified in the microarrays in the GSE13904 dataset. **B** Heat map of 6,073 differentially expressed mRNAs (including 2,217 upregulated and 3,856 downregulated mRNAs) identified in the microarrays in the GSE13904 dataset.

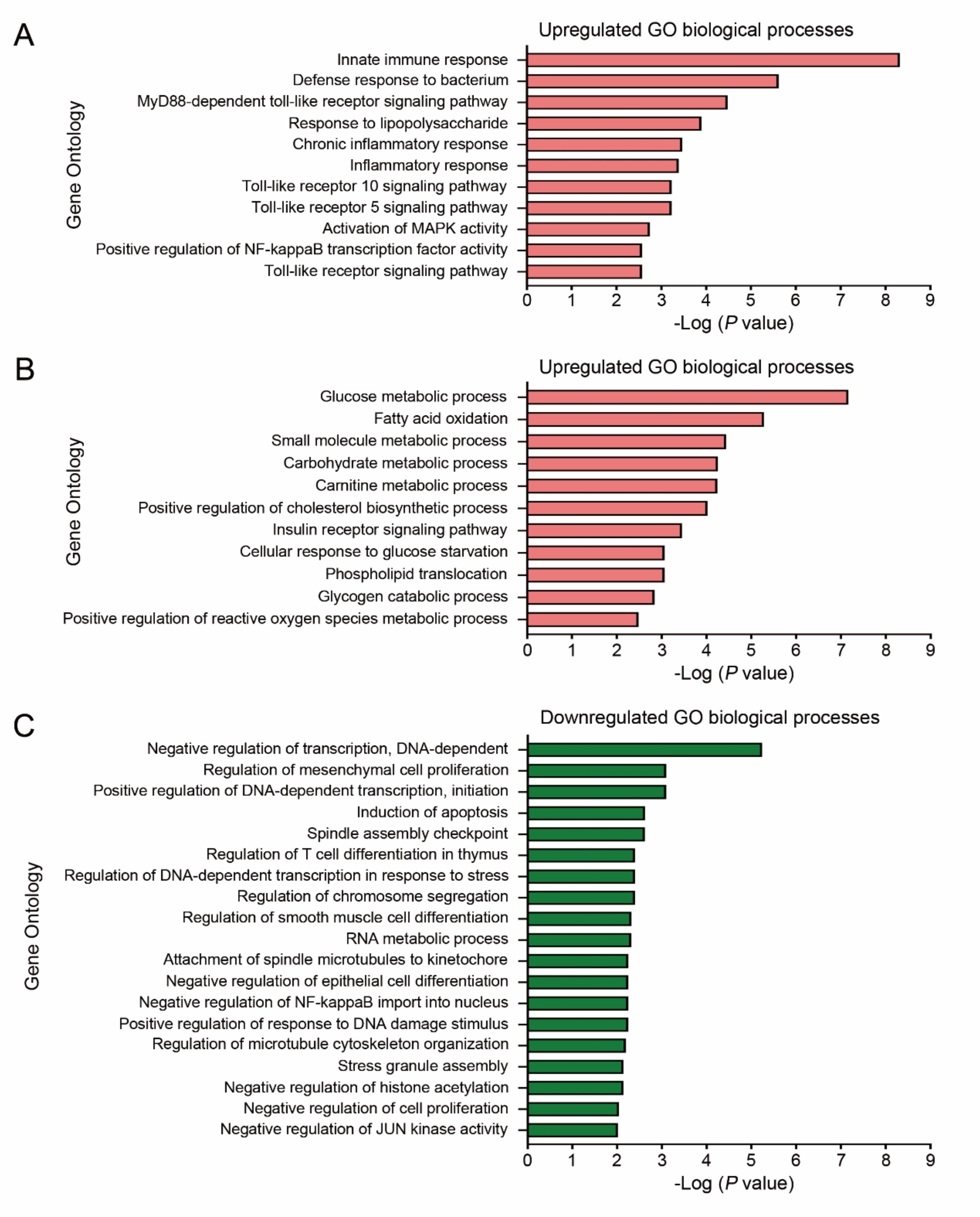

Supplementary Fig. 3 GO biological process of the co-expressed mRNAs. A Significantly upregulated biological processes were related to the immune response. **B** Significantly upregulated biological processes were related to metabolic processes. **C** Significantly downregulated biological processes were related to cell differentiation and gene transcription. The y-axis shows the GO category and the x-axis shows the negative logarithm of the *P* value (-Log (*P* value)).

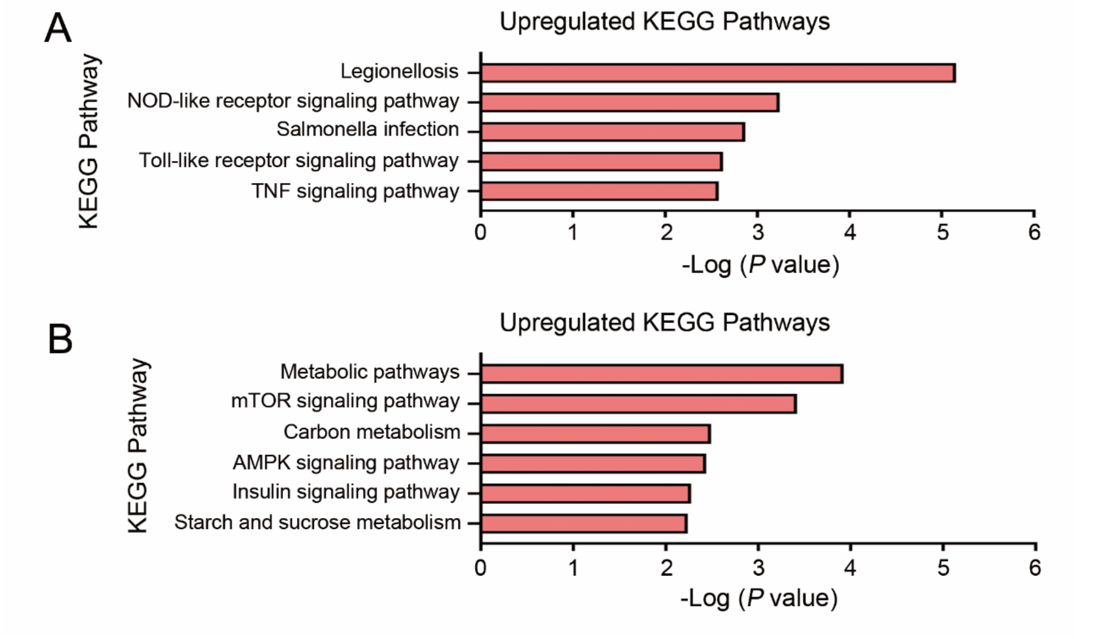

Supplementary Fig. 4 KEGG Pathway analysis of the co-expressed mRNAs. A Significantly upregulated pathways were related to the immune response. **B** Significantly upregulated pathways were related to metabolic processes. The y-axis shows the pathway category and the x-axis shows the negative logarithm of the *P* value (-Log (*P* value)).

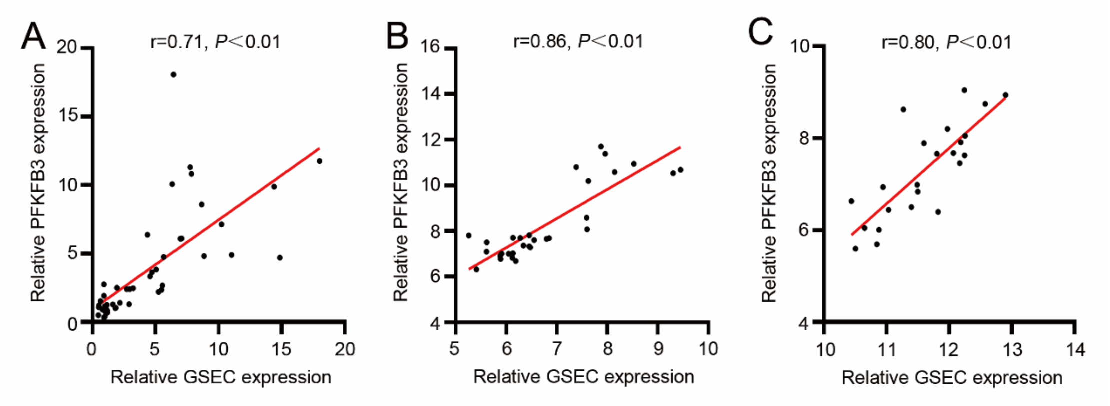

Supplementary Fig. 5 Correlation of GSEC and PFKFB3 genes in immunocytes of septic patients from 3 GEO datasets. A Correlation of GSEC and PFKFB3 genes in septic leucocytes in the GSE13904 dataset. **B** Correlation of the GSEC and PFKFB3 genes in septic leucocytes in the GSE28750 dataset. **C** Correlation of the GSEC and PFKFB3 genes in septic neutrophils in the GSE64457 dataset. Statistics were calculated using Pearson analysis.

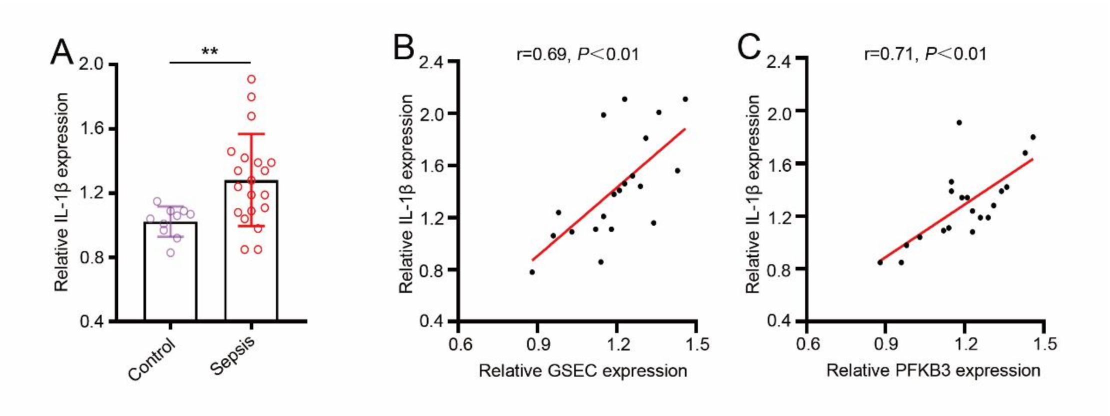

Supplementary Fig. 6 Expression and correlation of interleukin (IL)-1β gene in septic neutrophils. A Expression of IL-1β gene in septic neutrophils. **B** Correlation of IL-1β and GSEC in septic neutrophils. **C** Correlation of IL-1β and PFKFB3 mRNA in septic neutrophils. For each group: septic patients: n = 20, healthy volunteers: n = 10, three independent experiments. Statistics were calculated using Student's t test (A) or Pearson analysis (B and C). $^{**}P < 0.01$.

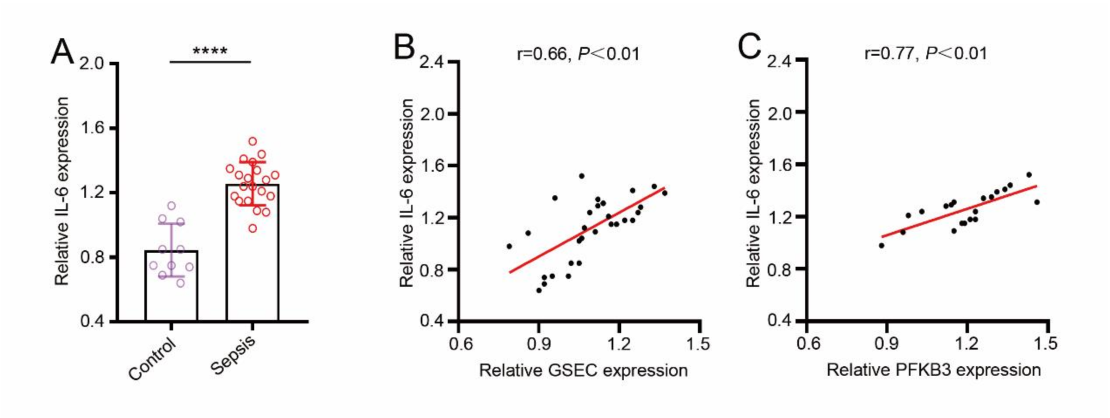

Supplementary Fig. 7 Expression and correlation of IL-6 gene in septic neutrophils. **A** Expression of IL-6 gene in septic neutrophils. **B** Correlation of IL-6 and GSEC in septic neutrophils. **C** Correlation of IL-6 and PFKFB3 mRNA in septic neutrophils. For each group: septic patients: n = 20, healthy volunteers: n = 10, three independent experiments. Statistics were calculated using Student's t test (A) or Pearson analysis (B and C). ****P< 0.0001.

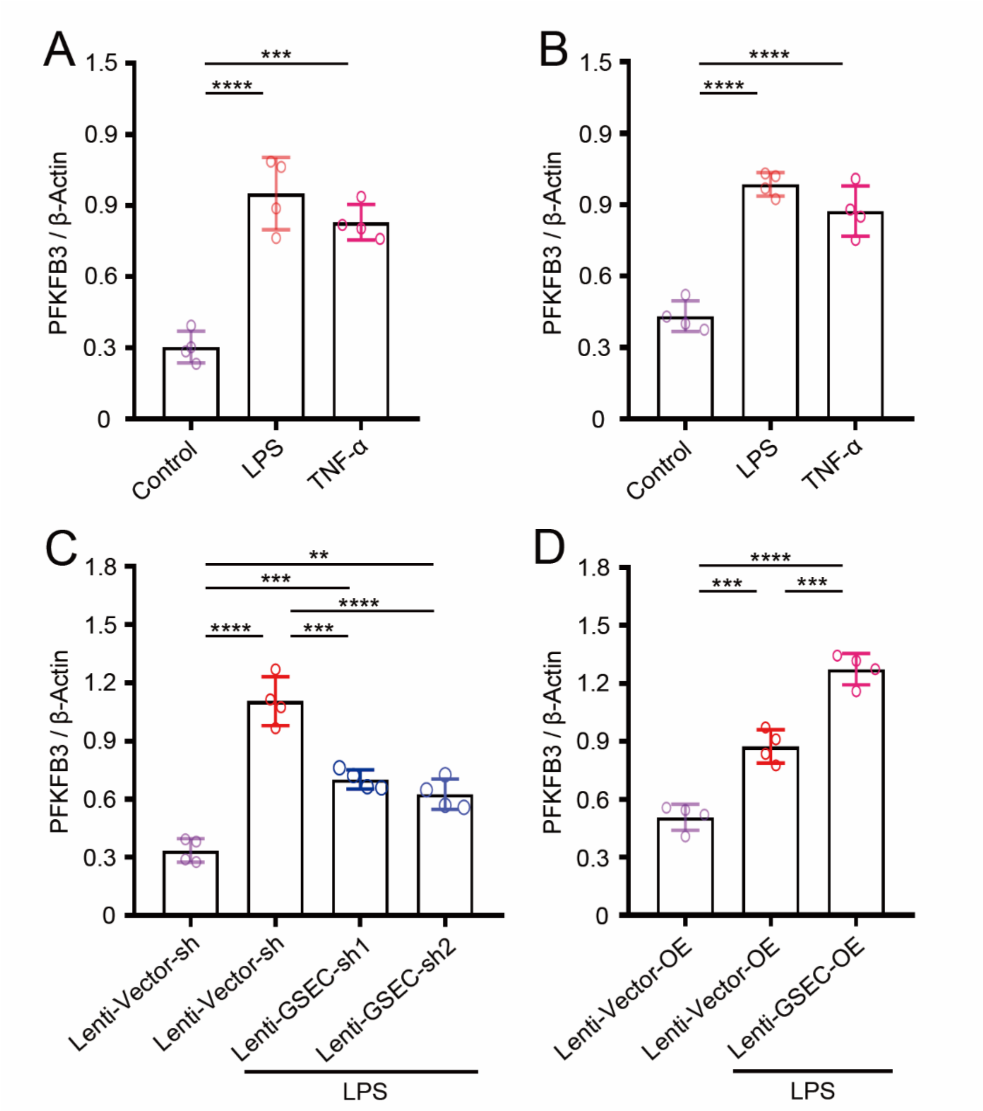

Supplementary Fig. 8 The average ratio of PFKFB3/β-Actin. A The average ratio of PFKFB3/β-Actin in primary human neutrophils. **B** The average ratio of PFKFB3/β-Actin in dHL-60 cells. **C** The average ratio of PFKFB3/β-Actin in GSEC-knockdown dHL-60 cells. **D** The average ratio of PFKFB3/β-Actin in GSEC-overexpressing dHL-60 cells. For all experiments: n = 4 per group, three independent experiments. Statistics were calculated using one-way ANOVA. $^{**}P< 0.01$, $^{***}P< 0.001$, $^{****}P< 0.0001$.

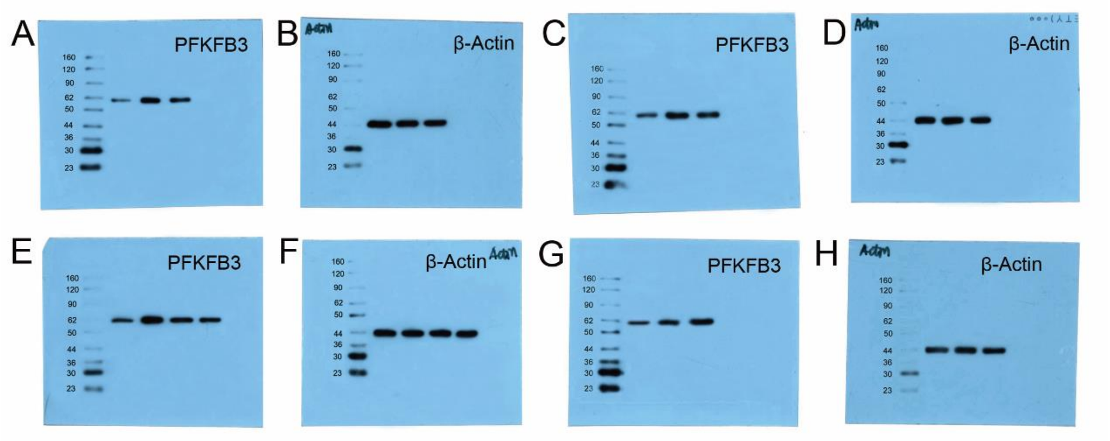

Supplementary Fig. 9 The original western blot bands of PFKFB3 and β-Actin. **A** The original bands of PFKFB3 in primary human neutrophils. **B** The original bands of β-Actin in primary human neutrophils. **C** The original bands of PFKFB3 in dHL-60 cells. **D** The original bands of β-Actin in dHL-60 cells. **E** The original bands of PFKFB3 in GSEC-knockdown dHL-60 cells. **F** The original bands of β-Actin in GSEC-knockdown dHL-60 cells. **G** The original bands of PFKFB3 in GSEC-overexpressing dHL-60 cells. **H** The original bands of β-Actin in GSEC-overexpressing dHL-60 cells.

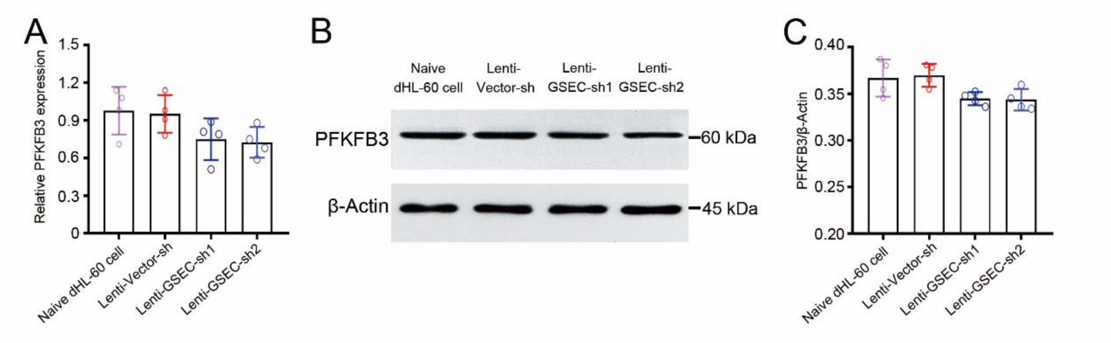

Supplementary Fig. 10 The expression of PFKFB3 in GSEC-knockdown dHL-60 cells without LPS stimulation. A GSEC knockdown did not decrease the expression of PFKFB3 mRNA in dHL-60 cells without LPS stimulation. **B** Western blot analysis showed that GSEC knockdown did not decrease the expression of PFKFB3 in dHL-60 cells without LPS stimulation. **C** The average ratio of PFKFB3/β-Actin in GSEC-knockdown dHL-60 cells without LPS stimulation. Neutrophils and dHL-60 cells were treated LPS or TNF-α for 12 hours. For all experiments: n = 4 per group, three independent experiments. Statistics were calculated using one-way ANOVA with Tukey post-tests.

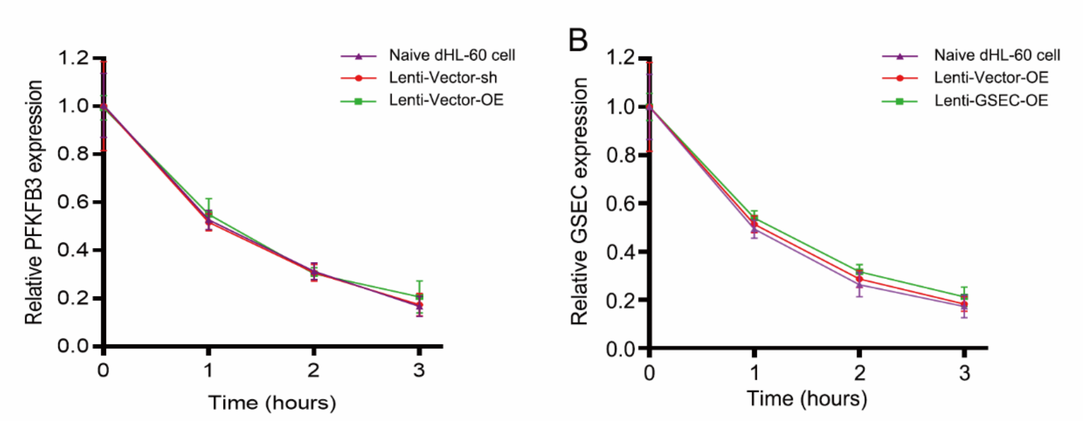

Supplementary Fig. 11 The stability of PFKFB3 expression in Lentivirus vectors stimulation and GSEC-overexpressing dHL-60 cells. **A** Lentivirus vectors stimulation did not influence the stability of PFKFB3 mRNA. **B** GSEC-overexpression did not reduce the decay rate of GSEC lncRNA in dHL-60 cells. For all experiments: n = 4 per group, three independent experiments.

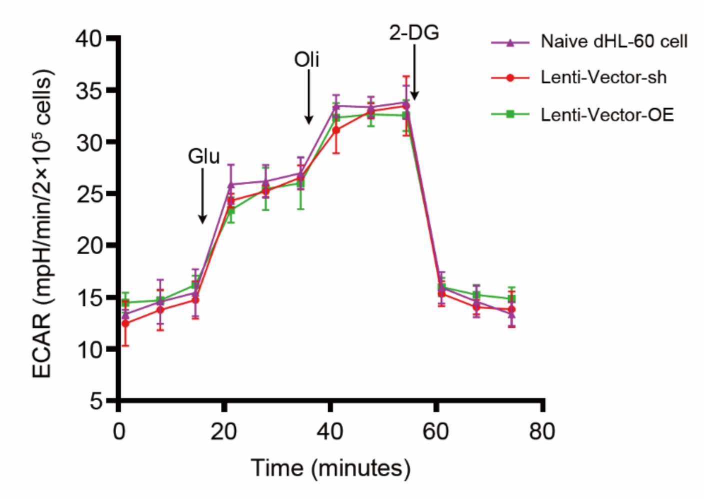

Supplementary Fig. 12 ECAR level between empty vectors and naive dHL-60 cells. There was no statistically difference of ECAR level between empty vectors and naive dHL-60 cells. For all experiments: n = 4 per group, three independent experiments. Glu: glucose; Oli: oligomycin; 2-DG: 2-deoxyglucose.

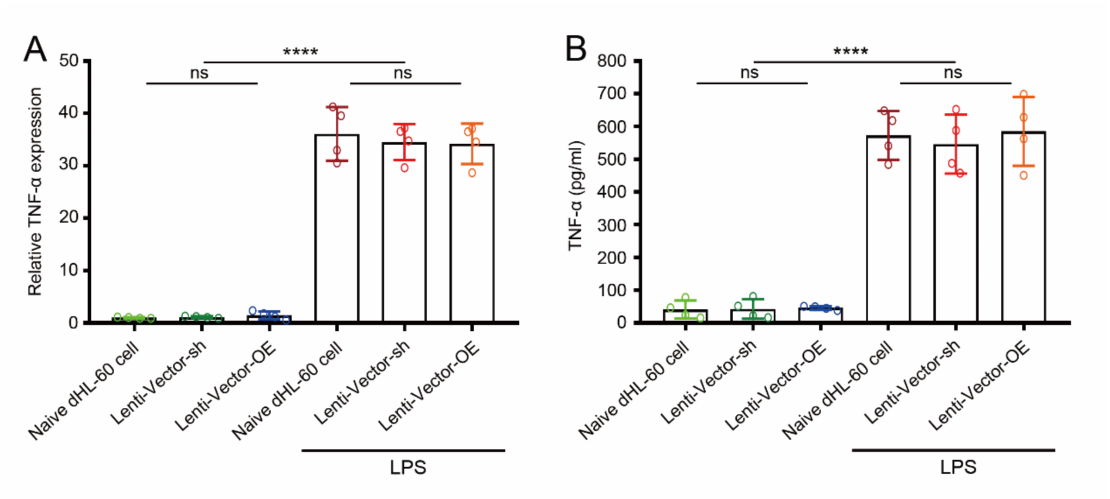

Supplementary Fig. 13 TNF-α level between empty vectors and naive dHL-60 cells. **A** The lentivirus vectors do not affect the expression of TNF-α mRNA in dHL-60 cells with or without LPS stimulation. **B** The lentivirus vectors do not affect the level of TNF-α proteins in dHL-60 cells with or without LPS stimulation. For all experiments: n = 4 per group, three independent experiments. Statistics were calculated using one-way ANOVA with Tukey post-tests. ****P*<0.0001, ^{ns}P indicate no statistical.

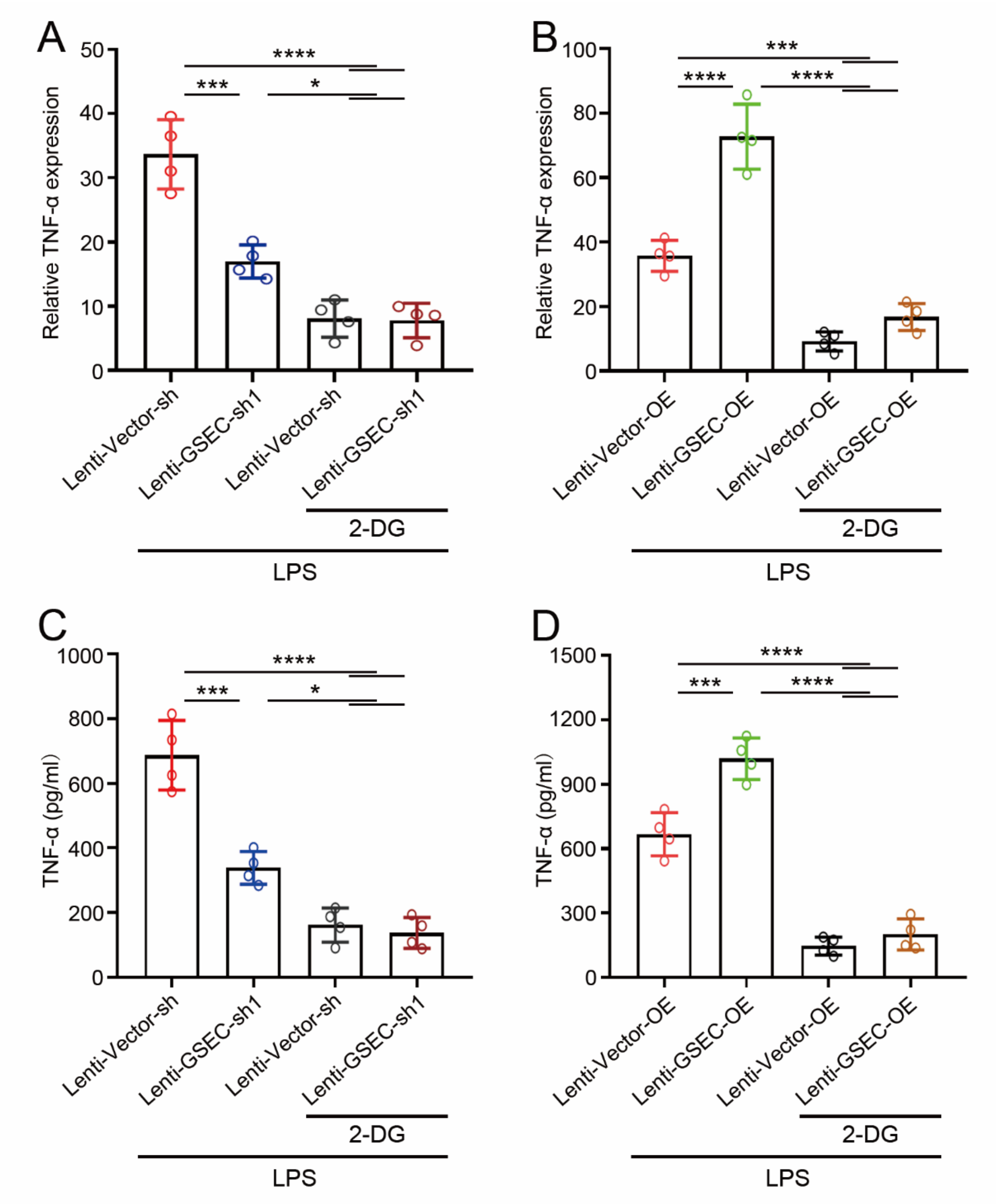

Supplementary Fig. 14 Treatment with 2-Deoxy-D-Glucose (2-DG) reduced the expression of TNF-α. A 2-DG reduced the expression of TNF-α mRNA in dHL-60 cells with or without GSEC-knockdown. **B** 2-DG reduced the expression of TNF-α mRNA in dHL-60 cells with or without GSEC-overexpression. **C** 2-DG reduced the level of TNF-α proteins in dHL-60 cells with or without GSEC-knockdown. **D** 2-DG reduced the level of TNF-α proteins in dHL-60 cells with or without GSEC-overexpression. For all experiments: n = 4 per group, three independent experiments. Statistics were calculated using one-way ANOVA with Tukey post-tests. *$P < 0.05$, ***$P < 0.001$, ****$P < 0.0001$.

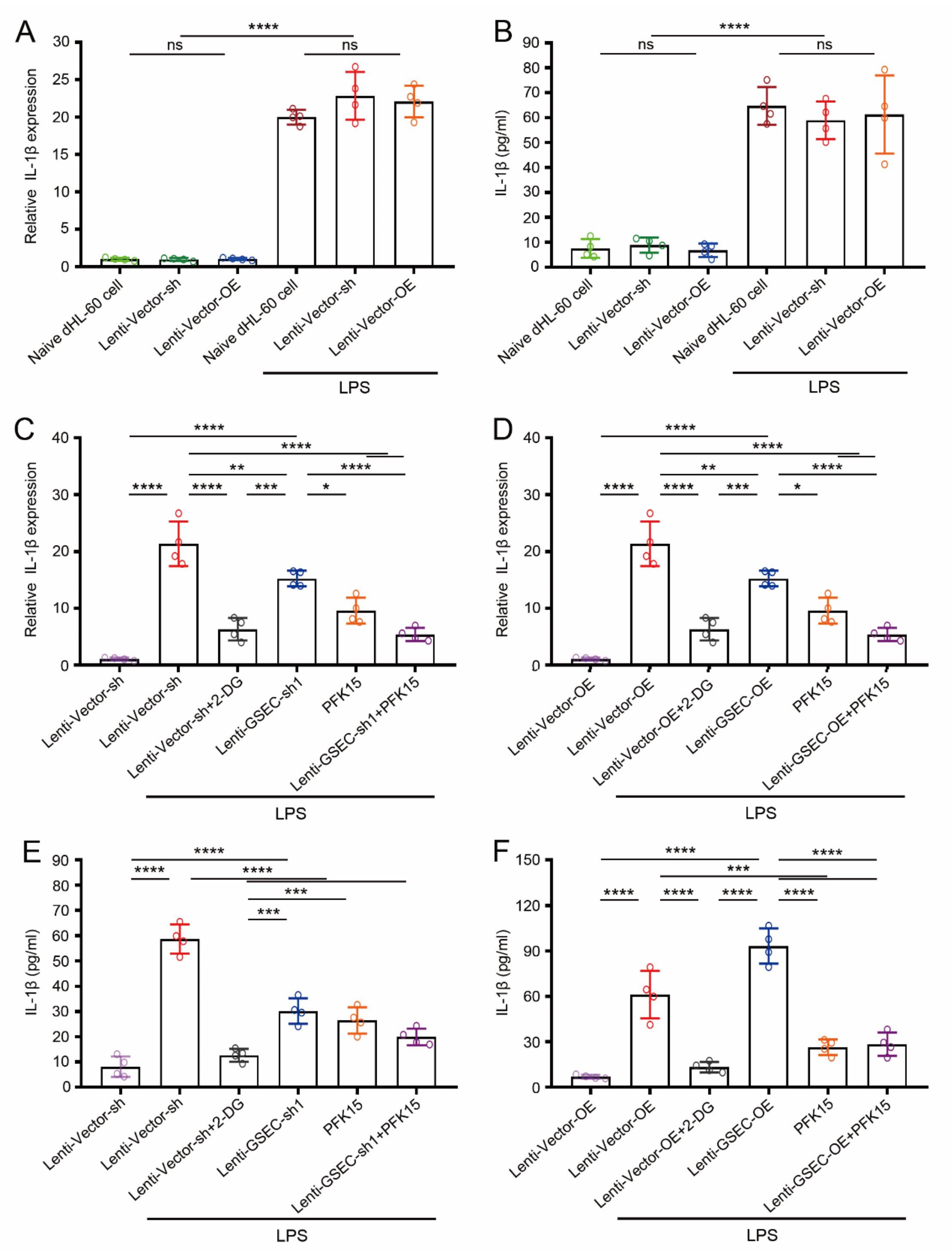

Supplementary Fig. 15 The level of IL-1β in LPS stimulated dHL-60 cells. A The level of IL-1β mRNA was detected in lentivirus vectors transfected dHL-60 cells with or without LPS stimulation. **B** The level of IL-1β protein was detected in lentivirus vectors transfected dHL-60 cells with or without LPS stimulation. **C** The level of IL-1β mRNA was detected in lncRNA GSEC-knockdown dHL-60 cells. **D** The level of IL-1β mRNA was detected in lncRNA GSEC-overexpressing dHL-60 cells. **E** The level of IL-1β protein was detected in lncRNA GSEC-knockdown dHL-60 cells. **F** The level of IL-1β protein was detected in lncRNA GSEC-overexpressing dHL-60 cells. For all experiments: n = 4 per group, three independent experiments. Statistics were calculated using one-way ANOVA with Tukey post-tests. **P*<0.05, ***P*<0.01, ****P*<0.001, ****P*<0.0001, ^{ns}P indicate no statistical.

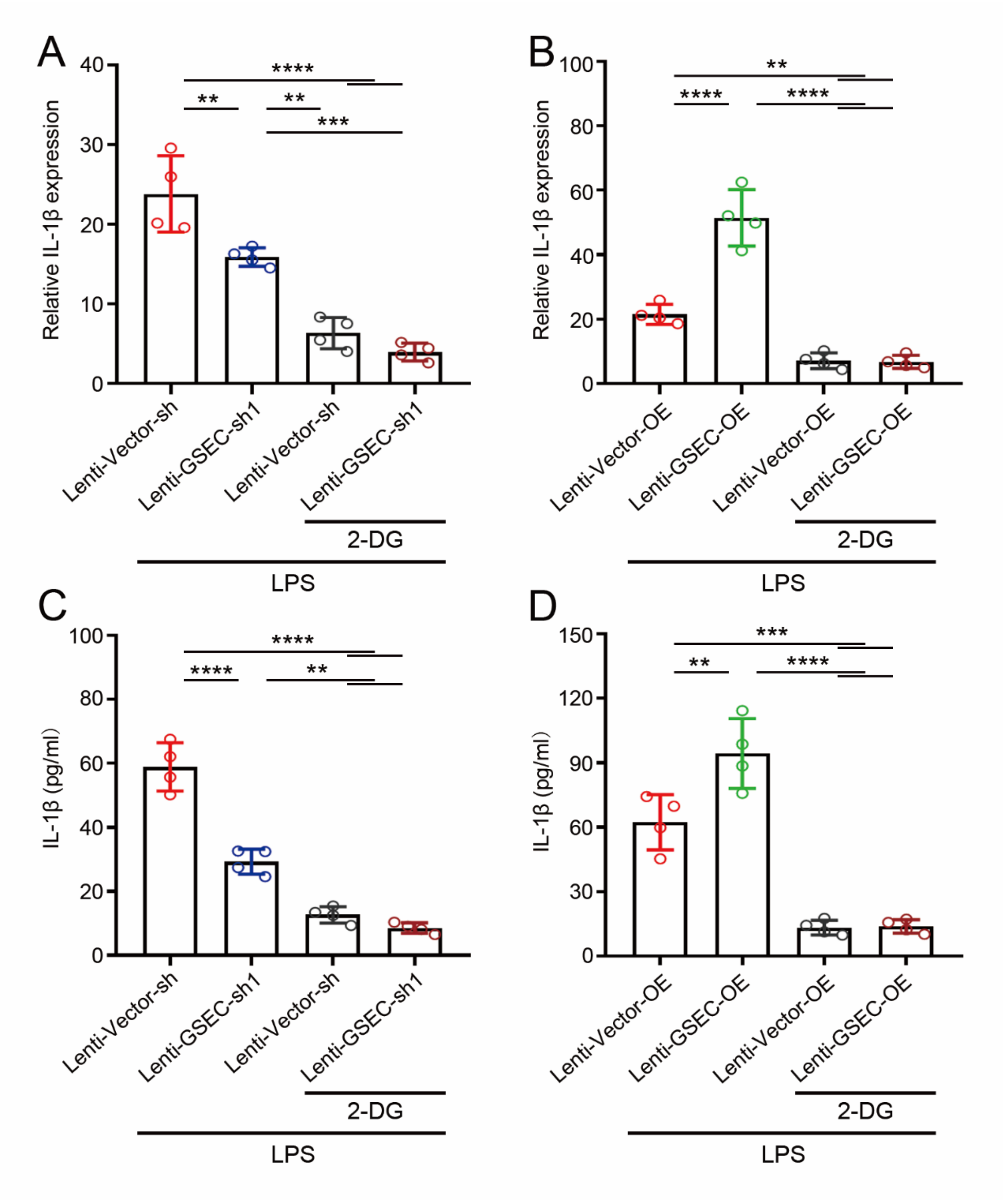

Supplementary Fig. 16 Treatment with 2-DG reduced the expression of IL-1β. A 2-DG reduced the expression of IL-1β mRNA in dHL-60 cells with or without GSEC-knockdown. **B** 2-DG reduced the expression of IL-1β mRNA in dHL-60 cells with or without GSEC-overexpression. **C** 2-DG reduced the level of IL-1β proteins in dHL-60 cells with or without GSEC-knockdown. **D** 2-DG reduced the level of IL-1β proteins in dHL-60 cells with or without GSEC-overexpression. For all experiments: n = 4 per group, three independent experiments. Statistics were calculated using one-way ANOVA with Tukey post-tests. **P*< 0.05, ****P*< 0.001, *****P*< 0.0001.

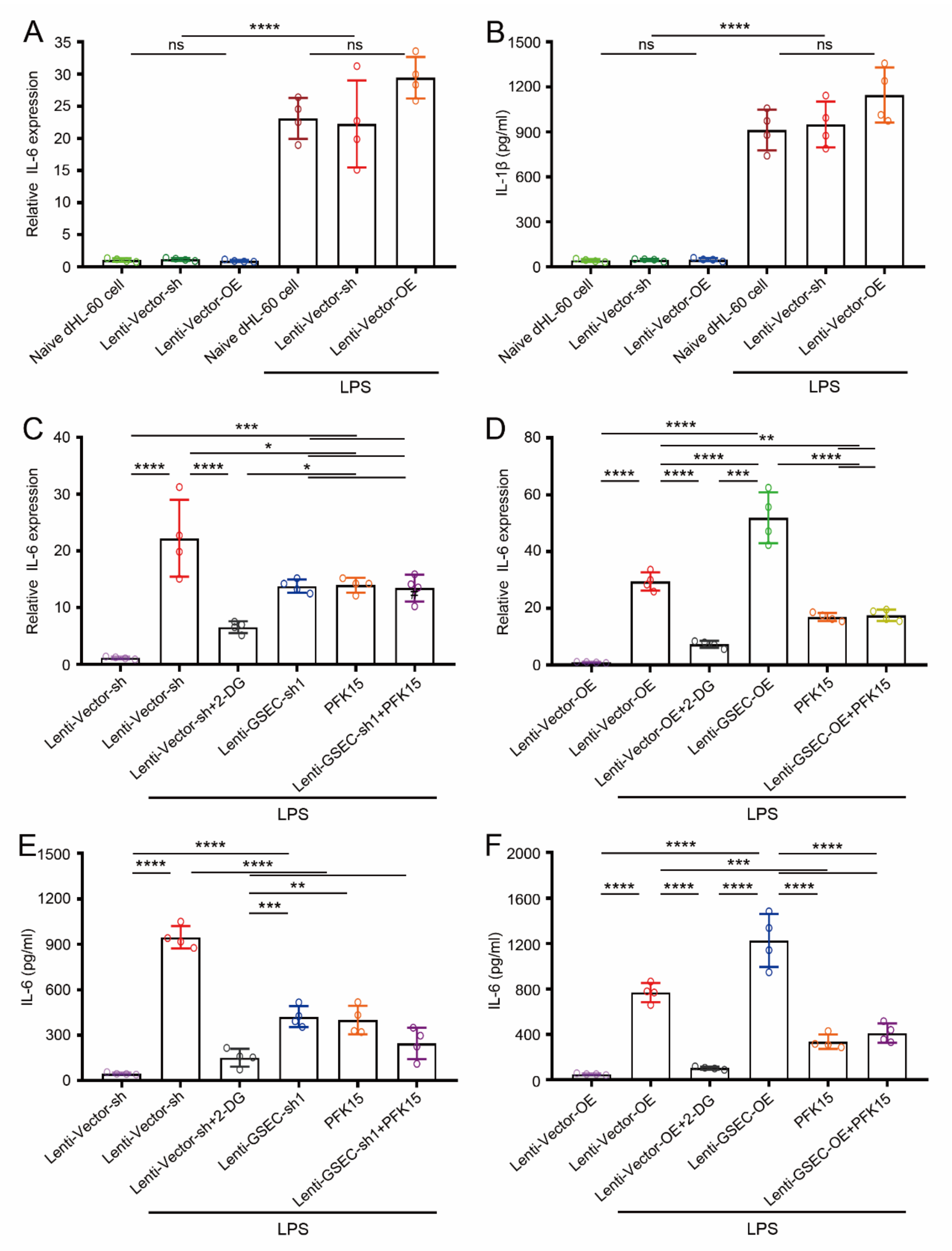

Supplementary Fig. 17 The level of IL-6 in LPS stimulated dHL-60 cells. A The level of IL-6 mRNA was detected in lentivirus vectors transfected dHL-60 cells with or without LPS stimulation. **B** The level of IL-6 protein was detected in lentivirus vectors transfected dHL-60 cells with or without LPS stimulation. **C** The level of IL-6 mRNA was detected in lncRNA GSEC-knockdown dHL-60 cells. **D** The level of IL-6 mRNA was detected in lncRNA GSEC-overexpressing dHL-60 cells. **E** The level of IL-6 protein was detected in lncRNA GSEC-knockdown dHL-60 cells. **F** The level of IL-6 protein was detected in lncRNA GSEC-overexpressing dHL-60 cells. For all experiments: n = 4 per group, three independent experiments. Statistics were calculated using one-way ANOVA with Tukey post-tests. **P*<0.05, ***P*<0.01, ****P*<0.001, ****P*<0.0001, ^{ns}P indicate no statistical.

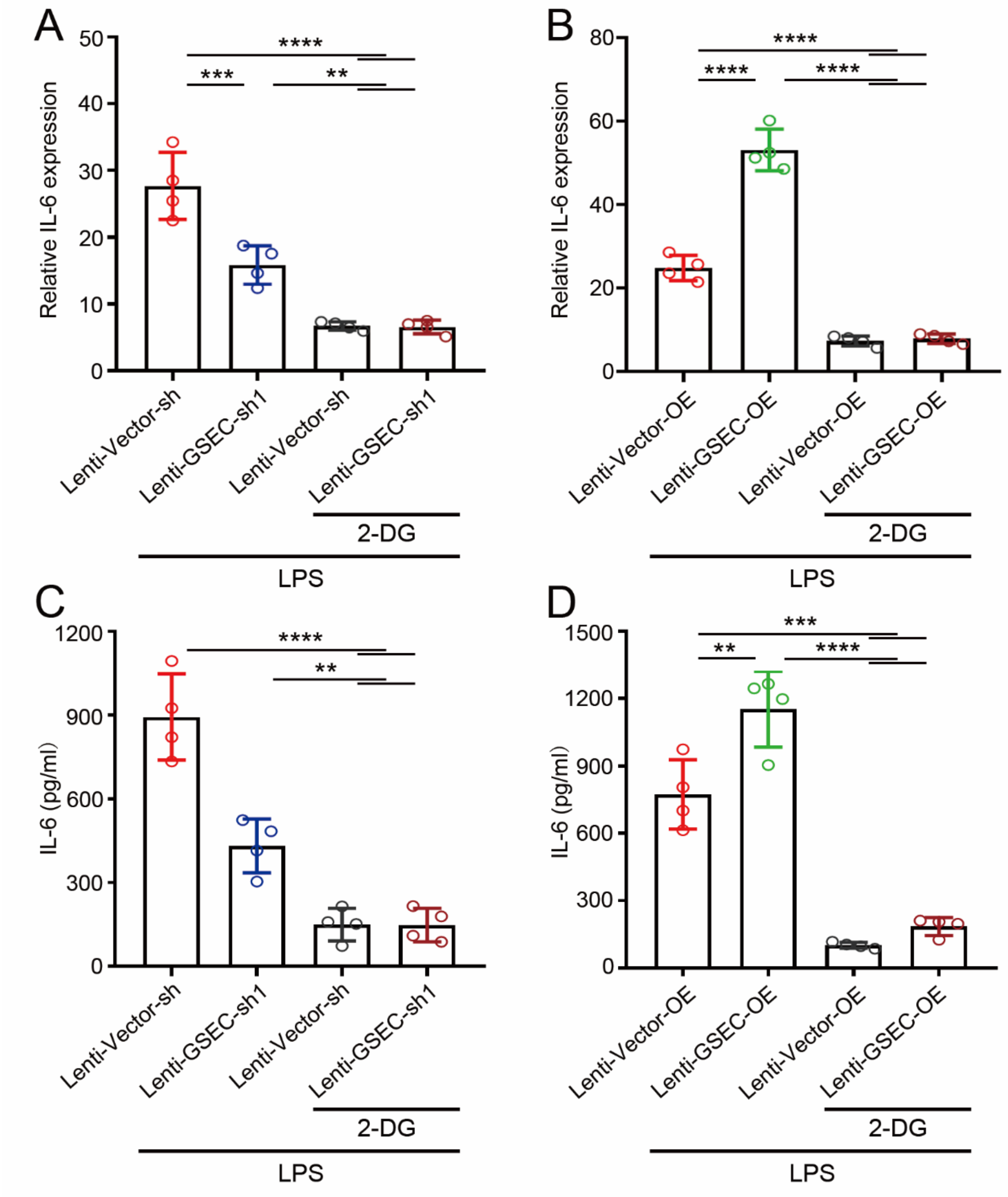

Supplementary Fig. 18 Treatment with 2-DG reduced the expression of IL-6. A 2-DG reduced the expression of IL-6 mRNA in dHL-60 cells with or without GSEC-knockdown. **B** 2-DG reduced the expression of IL-6 mRNA in dHL-60 cells with or without GSEC-overexpression. **C** 2-DG reduced the level of IL-6 proteins in dHL-60 cells with or without GSEC-knockdown. **D** 2-DG reduced the level of IL-6 proteins in dHL-60 cells with or without GSEC- overexpression. For all experiments: n = 4 per group, three independent experiments. Statistics were calculated using one-way ANOVA with Tukey post-tests. **P*< 0.05, ****P*< 0.001, *****P*< 0.0001.

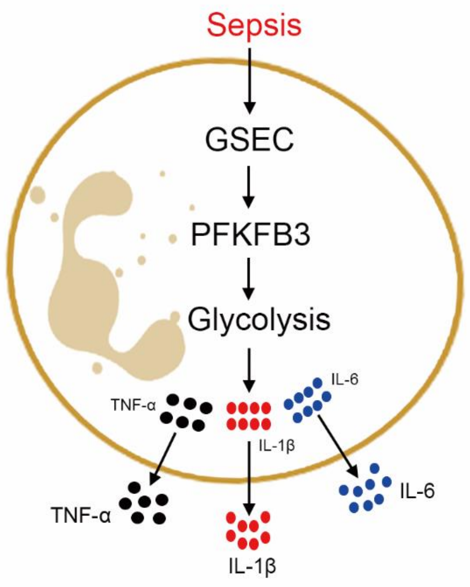

Supplementary Fig. 19 GSEC regulated PFKFB3-mediated glycolytic reprogramming is a potential sepsis therapeutic target. GSEC lncRNA has a pivotal role in promoting glycolysis of neutrophils by enhancing PFKFB3 transcription and translation, thus facilitating the neutrophil inflammatory factors production during the acute phase of sepsis.

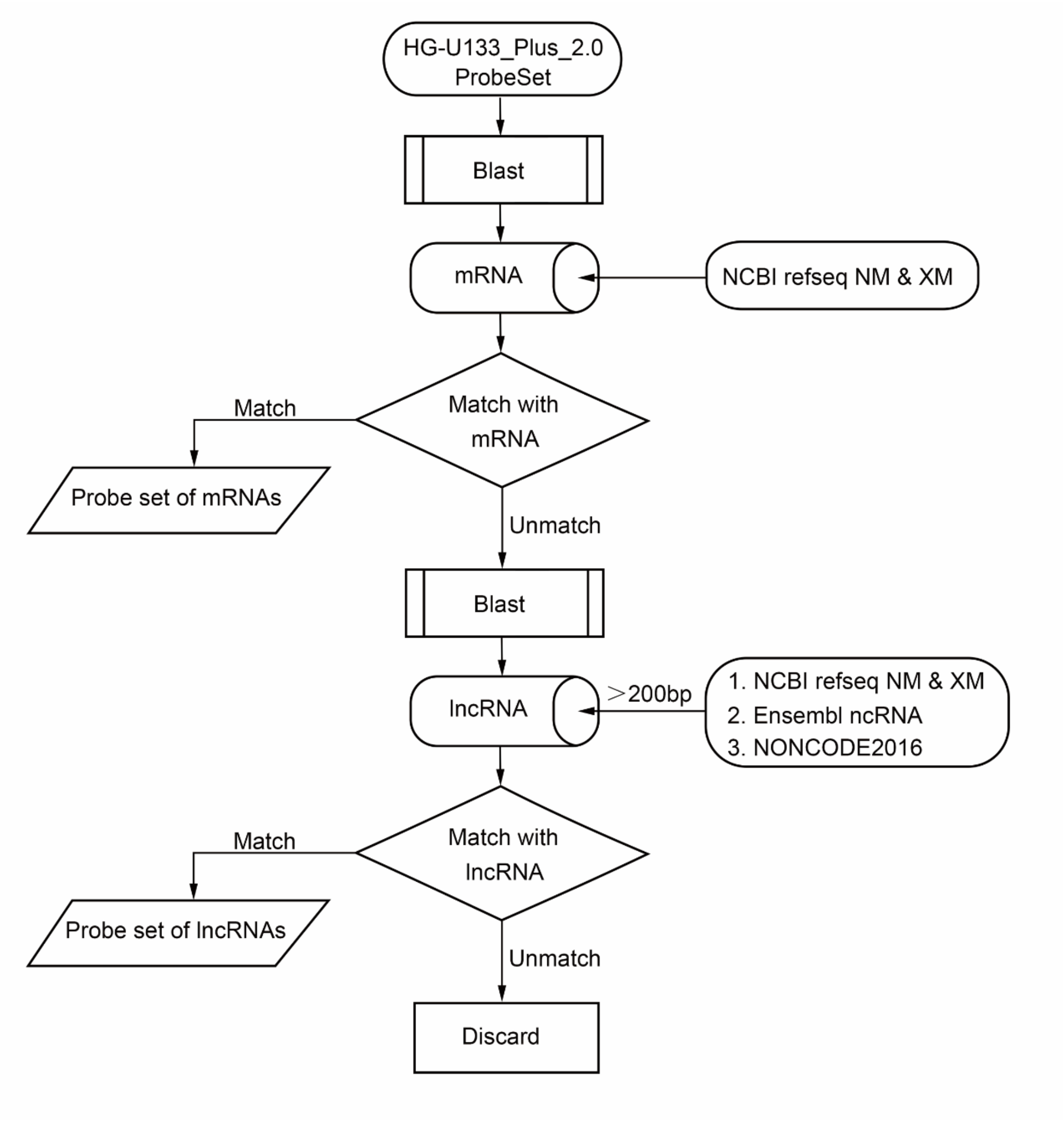

Supplementary Fig. 20 The work flow of the HG-U133_Plus_2.0 annotation.

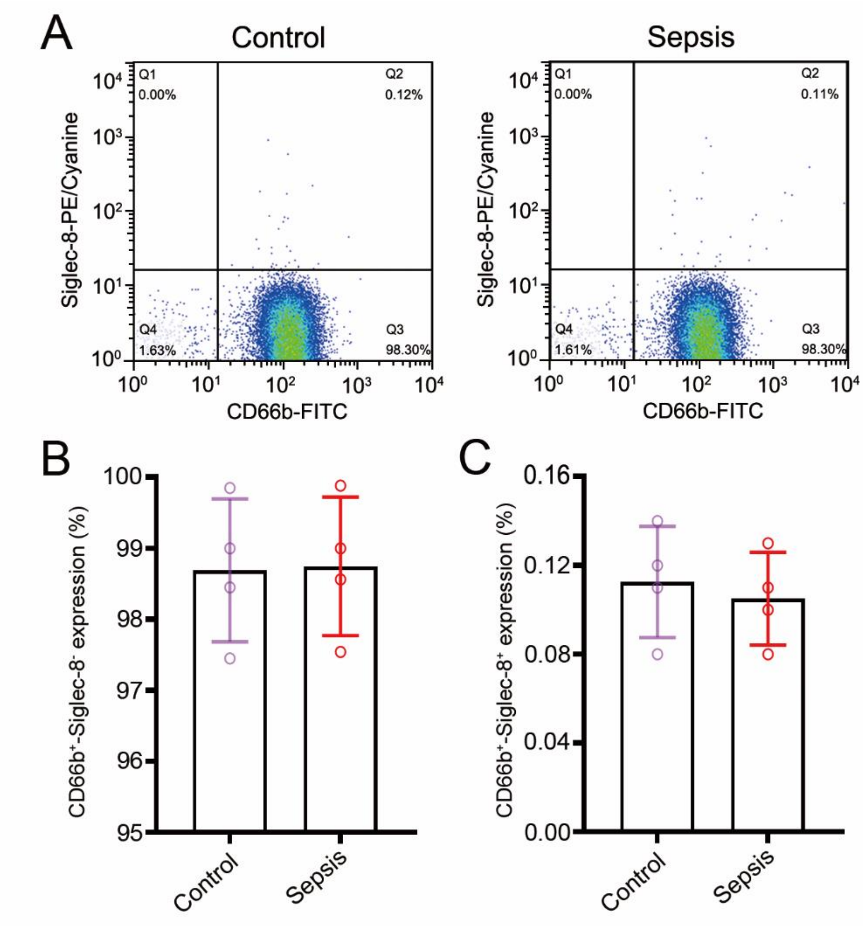

Supplementary Fig. 21 The purity of neutrophils was detected and adjusted by flow cytometry with co-staining CD66b with Siglec-8. **A** Representative images of neutrophils detection by flow cytometry. **B** Mean fluorescence intensity of CD66b^{+}-Siglec-8^{-} cells. **C** Mean fluorescence intensity of CD66b^{+}-Siglec-8^{+} cells.

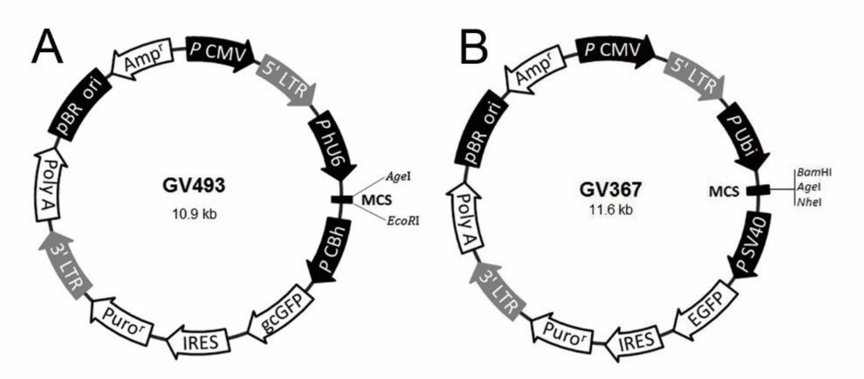

Supplementary Fig. 22 Schematic of lentivirus vectors. A. Schematic of the GV493 lentivirus vector. Two specific target sequences for GSEC (5'-GGTCACAACAGTACAAAGA-3', and 5'-CCAACTATGCCATGGTCTT-3') were respectively cloned into the MCS region of GV493 (hU6-MCS-CBh-gcGFP-IRES-puromycin) to construct knock down lentivirus vectors of GSEC, named as Lenti-GSEC-sh1 and Lenti-GSEC-sh2. **B** Schematic of the GV367 lentivirus vector. A specific target sequence for GSEC (NONHSAT160878.1) was cloned into the MCS region of GV367 (Ubi-MCS-SV40-EGFP-IRES-puromycin) to construct overexpression lentivirus vectors of GSEC, named as Lenti-GSEC-OE.

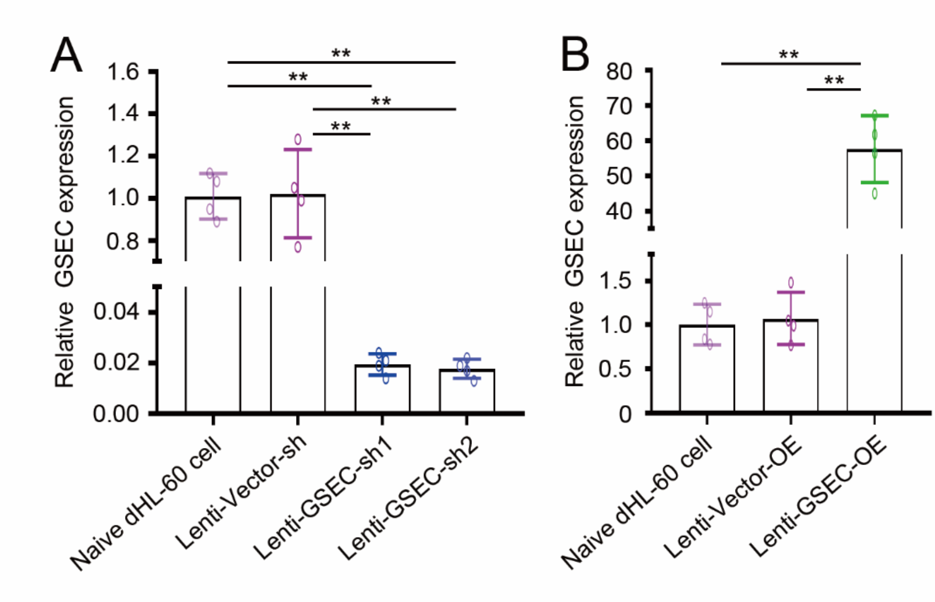

Supplementary Fig. 23 The expression of GSEC in dHL-60 cells. A Both Lenti-GSEC-sh1 and Lenti-GSEC-sh2 significantly reduced the expression of GSEC, and lenti-vector-sh does not reduce the expression of GSEC. **B** Lenti-GSEC-OE significantly increased the expression of GSEC, and lenti-vector-OE does not increase the expression of GSEC. For all experiments: n = 4 per group, three independent experiments. ***P*<0.01.

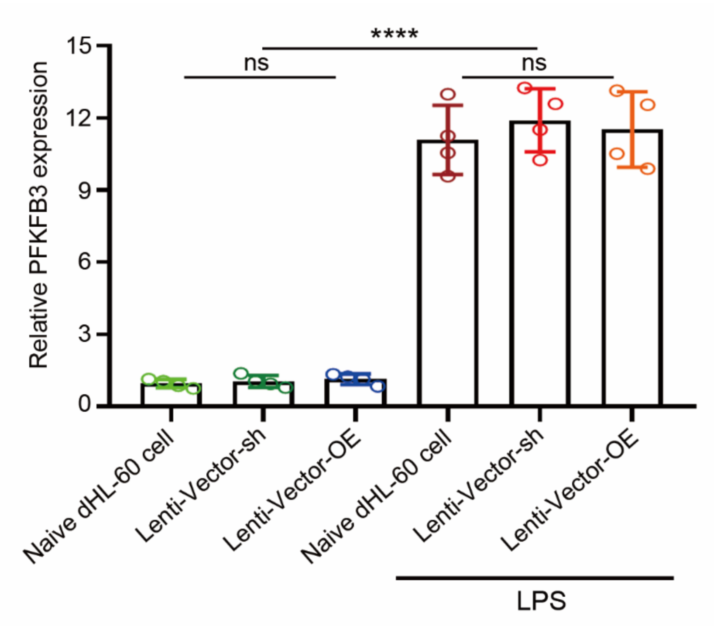

Supplementary Fig. 24. Expression of PFKFB3 mRNA between empty vectors and naive dHL-60 cells. The lentivirus vectors do not affect the expression of PFKFB3 mRNA in dHL-60 cells with or without LPS stimulation. For all experiments: n = 4 per group, three independent experiments. Statistics were calculated using one-way ANOVA with Tukey post-tests. ****P*<0.0001, ^{ns}P indicate no statistical.
