# Supplementary material for: Long noncoding RNA GSEC promotes neutrophil inflammatory activation by supporting PFKFB3-involved glycolytic metabolism in sepsis
Source: Cell Death Dis. 2021 Dec 14;12(12):1157. doi: 10.1038/s41419-021-04428-7 (PMC8671582; doi:10.1038/s41419-021-04428-7)
Supplement: Supplementary file 3 — Supplementary Table 1 [file 41419_2021_4428_MOESM3_ESM.pdf]

Supplementary Table 1. 33 overlapped lncRNAs of 3 datasets

lncRNA	style	Mean Signal of Group Sepsis(GSE13904)	Mean Signal of Group Control(GSE13904)	Fold Change(GSE13904)	FDR(GSE13904)
ENST00000555562.1	up	5.64269	5.347633	1.23	9.81E-03
NONHSAT100917.2	up	5.842245	5.506454	1.26	3.62E-02
NONHSAT211528.1	up	6.050075	5.652882	1.32	9.81E-03
NONHSAT198787.1	up	3.893279	3.450267	1.36	4.93E-04
NONHSAT094090.2	up	5.915071	5.468026	1.36	3.62E-02
NONHSAT216480.1	up	4.77362	4.323711	1.37	1.99E-02
ENST00000441749.1	up	5.701936	5.248951	1.37	7.03E-04
ENST00000607967.1	up	6.627577	6.167803	1.38	9.81E-03
NONHSAT164381.1	up	5.2277885	4.6309415	1.51	1.41E-04
NONHSAT006710.2	up	6.541353	5.749878	1.73	0.00E+00
XR_941546.1	up	4.576459	3.763056	1.76	2.82E-04
ENST00000519323.1	up	5.246531	4.239477	2.01	0.00E+00
NONHSAT150799.1	up	5.336991	4.303819	2.05	0.00E+00
NONHSAT200806.1	up	6.808582	5.74038	2.10	0.00E+00
NONHSAT010176.2	up	6.6163675	5.4890875	2.32	0.00E+00
NONHSAT034261.2	up	6.053029	4.836349	2.32	0.00E+00
ENST00000629441.1	up	6.686111	5.215516	2.77	0.00E+00
NONHSAT081776.2	up	5.8891755	4.4299625	2.82	0.00E+00
XR_926068.1	up	6.836203	5.210496	3.09	0.00E+00
NONHSAT181667.1	up	7.24628	5.43127	3.52	0.00E+00
NONHSAT160878.1	up	7.482223	5.257068	4.68	0.00E+00
NONHSAT131038.2	down	6.9119155	7.8947645	-1.98	1.41E-04
NONHSAT002346.2	down	7.263435	8.137209	-1.83	7.03E-04
NONHSAT009146.2	down	6.620488	7.39816	-1.71	1.71E-03
ENST00000608064.1	down	5.772428	6.536868	-1.70	0.00E+00
NONHSAT134698.2	down	5.851227	6.599442	-1.68	0.00E+00
NONHSAT131053.2	down	7.244893	7.982082	-1.67	4.47E-03
ENST00000612387.1	down	5.528728	6.147776	-1.54	2.82E-04
NR_003945.1	down	7.30771	7.907173	-1.52	1.99E-02
NONHSAT115567.2	down	3.56243	4.121644	-1.47	7.03E-04
XR_918449.1	down	4.430633	4.980176	-1.46	4.47E-03
NONHSAT009860.2	down	5.678602	6.153917	-1.39	9.81E-03
NONHSAT102622.2	down	4.458923	4.892148	-1.35	0.00E+00

p-value(GSE13904)	Mean Signal of Group Sepsis(GSE28750)	Mean Signal of Group Control(GSE28750)	Fold Change(GSE28750)	FDR(GSE28750)
8.11E-03	6.093388	5.659928	1.35	1.40E-03
2.46E-02	6.242706	5.505013	1.67	0.00E+00
1.12E-02	6.560805	5.618696	1.92	0.00E+00
1.61E-03	3.38204	3.031732667	1.28	2.47E-03
2.56E-02	6.402553	5.862841	1.45	1.40E-03
1.68E-02	4.135485	3.612178	1.44	0.00E+00
1.85E-03	5.765401	5.377021	1.31	1.56E-02
8.57E-03	7.126769	6.754033	1.29	1.56E-02
9.70E-04	5.6502635	4.7737335	1.86	2.42E-04
1.13E-04	6.815032	6.130347	1.61	0.00E+00
1.09E-03	4.556502	3.292295	2.40	0.00E+00
4.65E-04	5.928382	4.192942	3.33	0.00E+00
2.08E-04	6.272206	5.289751	1.98	4.83E-04
8.40E-05	6.877776	6.009045	1.83	0.00E+00
3.83E-04	7.1454605	5.8481645	2.54	0.00E+00
1.02E-04	5.749597	4.304224	2.72	0.00E+00
3.60E-05	7.463312	5.711721	3.37	0.00E+00
4.65E-05	6.8753395	4.945581	3.86	0.00E+00
2.50E-05	6.250143	4.926417	2.50	0.00E+00
2.50E-05	7.833467	6.204771	3.09	0.00E+00
1.90E-05	8.146526	6.117758	4.08	0.00E+00
8.91E-04	8.518912	9.174583	-1.58	0.00E+00
2.55E-03	7.106514	7.857799	-1.68	4.83E-04
5.78E-03	6.864264	7.950837	-2.12	0.00E+00
6.60E-05	5.659053	6.171996	-1.43	1.40E-03
4.60E-04	4.76585	6.304235	-2.90	0.00E+00
7.79E-03	6.822975	7.640092	-1.76	4.83E-04
2.23E-03	5.906223	6.552621	-1.57	4.83E-04
2.19E-02	6.764101	7.639629	-1.83	2.32E-04
3.89E-03	5.481512	5.892777	-1.33	7.19E-03
8.74E-03	4.579933	5.199388	-1.54	1.40E-03
1.69E-02	5.85262	6.461651	-1.53	1.40E-03
3.38E-04	4.750693	5.251968	-1.42	0.00E+00

p-value(GSE28750)	Mean Signal of Group Sepsis(GSE64457)	Mean Signal of Group Control(GSE64457)	Fold Change(GSE64457)	FDR(GSE64457)
4.71E-03	3.671216	3.367591	1.23	4.81E-02
2.41E-04	6.759132	5.846787	1.88	2.24E-02
6.72E-04	4.44461	3.328611	2.17	5.87E-03
3.40E-03	2.468579	2.052398	1.33	4.81E-02
3.81E-03	6.631587	5.653006	1.97	1.65E-03
6.97E-04	4.174868	3.30942	1.82	4.81E-02
1.34E-02	5.257129	4.535429	1.65	2.24E-02
1.59E-02	7.83121	7.302178	1.44	3.33E-02
2.17E-03	3.311429	2.55595	1.69	3.33E-02
8.10E-04	6.546593	5.868371	1.60	3.86E-03
6.87E-04	9.745832	6.529862	9.29	0.00E+00
3.13E-04	6.68225	5.323492	2.56	1.65E-03
2.57E-03	6.958998	6.332423	1.54	5.87E-03
1.25E-03	8.674465	7.728832	1.93	1.50E-02
7.50E-05	8.816649	8.215784	1.52	4.07E-02
7.85E-04	2.511083	2.103692	1.33	4.81E-02
1.90E-05	6.355196	5.68277	1.59	3.33E-02
2.05E-05	8.7355735	7.9329695	1.78	1.67E-02
1.10E-03	8.541502	7.665375	1.84	9.12E-03
3.22E-04	5.230427	4.495019	1.66	4.81E-02
1.90E-05	7.466609	6.219441	2.37	3.86E-03
6.89E-04	6.715401	7.4032955	-1.61	2.70E-02
4.52E-03	3.976935	4.744202	-1.70	9.12E-03
2.99E-04	4.116092	5.61524	-2.83	9.12E-03
7.85E-03	3.62846	4.543093	-1.89	1.65E-03
2.00E-05	5.440052	6.541045	-2.15	1.50E-02
5.22E-03	2.330815	2.866392	-1.45	3.86E-03
4.17E-03	2.955728	3.621478	-1.59	2.24E-02
3.57E-03	2.511739	4.244674	-3.32	0.00E+00
1.77E-02	3.574083	4.113434	-1.45	2.24E-02
8.36E-03	3.230642	4.335509	-2.15	2.33E-03
6.92E-03	2.202598	2.562813	-1.28	2.33E-03
9.27E-04	5.690786	6.211382	-1.43	1.50E-02

p-value(GSE64457)
1.88E-02
4.98E-03
1.09E-03
1.61E-02
1.64E-04
1.46E-02
4.53E-03
8.70E-03
1.18E-02
7.62E-04
2.40E-05
2.47E-04
1.02E-03
3.09E-03
1.04E-02
1.31E-02
9.21E-03
5.71E-03
1.56E-03
1.42E-02
6.03E-04
8.94E-03
2.59E-03
2.79E-03
1.94E-04
3.82E-03
7.10E-04
5.95E-03
8.00E-05
8.64E-03
4.18E-04
3.20E-04
3.27E-03
