# Supplementary material for: Long noncoding RNA GSEC promotes neutrophil inflammatory activation by supporting PFKFB3-involved glycolytic metabolism in sepsis
Source: Cell Death Dis. 2021 Dec 14;12(12):1157. doi: 10.1038/s41419-021-04428-7 (PMC8671582; doi:10.1038/s41419-021-04428-7)
Supplement: Supplementary file 4 — Supplementary Table 2 [file 41419_2021_4428_MOESM4_ESM.pdf]

Supplementary Table 2 . 878 overlapped mRNAs of 3 datasets.

Gene Symbol	Gene ID	Gene Description	style
AASDHPPT	60496	aminoadipate-semialdehyde dehydrogenase-phosphopantetheinyl transferase	down
ABHD16A	7920	abhydrolase domain containing 16A	up
ACAA1	30	acetyl-CoA acyltransferase 1	up
ACER3	55331	alkaline ceramidase 3	up
ACVR1B	91	activin A receptor type IB	up
ACVR2A	92	activin A receptor type IIA	down
ADAM17	6868	ADAM metallopeptidase domain 17	up
ADAM19	8728	ADAM metallopeptidase domain 19	up
ADAM28	10863	ADAM metallopeptidase domain 28	down
ADAM9	8754	ADAM metallopeptidase domain 9	up
ADGRE2	30817	adhesion G protein-coupled receptor E2	up
ADGRG3	222487	adhesion G protein-coupled receptor G3	up
ADK	132	adenosine kinase	down
ADM	133	adrenomedullin	up
ADORA3	140	adenosine A3 receptor	up
AGAP4	119016	ArfGAP with GTPase domain, ankyrin repeat and PH domain 4	down
AGFG1	3267	ArfGAP with FG repeats 1	up
AGPAT2	10555	1-acylglycerol-3-phosphate O-acyltransferase 2	up
AGTRAP	57085	angiotensin II receptor-associated protein	up
AIM2	9447	absent in melanoma 2	up
AKIRIN2	55122	akirin 2	up
ALDH3B1	221	aldehyde dehydrogenase 3 family, member B1	up
ALDOA	226	aldolase A, fructose-bisphosphate	up
ALG8	79053	ALG8, alpha-1,3-glucosyltransferase	down
ALPL	249	alkaline phosphatase, liver/bone/kidney	up
AMD1	262	adenosylmethionine decarboxylase 1	down
AMMECR1	9949	Alport syndrome, mental retardation, midface hypoplasia and elliptocytosis chromosomal region gene	down
AMPH	273	amphiphysin	up
ANAPC1	64682	anaphase promoting complex subunit 1	down
ANAPC13	25847	anaphase promoting complex subunit 13	down
ANAPC15	25906	anaphase promoting complex subunit 15	up
ANKRD11	29123	ankyrin repeat domain 11	down
ANKRD44	91526	ankyrin repeat domain 44	down

ANO10	55129	anoctamin 10	up
ANXA1	301	annexin A1	up
ANXA3	306	annexin A3	up
AP3M1	26985	adaptor-related protein complex 3, mu 1 subunit	down
APH1B	83464	APH1B gamma secretase subunit	up
APLP2	334	amyloid beta (A4) precursor-like protein 2	up
APOL3	80833	apolipoprotein L, 3	down
ARG1	383	arginase 1	up
ARHGAP24	83478	Rho GTPase activating protein 24	up
ARID1B	57492	AT rich interactive domain 1B (SWI1-like)	down
ARIH2	10425	ariadne RBR E3 ubiquitin protein ligase 2	down
ARL4A	10124	ADP-ribosylation factor like GTPase 4A	up
ARL8B	55207	ADP-ribosylation factor like GTPase 8B	up
ARPC1B	10095	actin related protein 2/3 complex subunit 1B	up
ARSD	414	arylsulfatase D	up
ASAH1	427	N-acylsphingosine amidohydrolase (acid ceramidase) 1	up
ASPH	444	aspartate beta-hydroxylase	up
ASXL1	171023	additional sex combs like transcriptional regulator 1	down
ATG9A	79065	autophagy related 9A	up
ATP11B	23200	ATPase, class VI, type 11B	up
ATP13A3	79572	ATPase type 13A3	up
ATP2B1	490	ATPase, Ca++ transporting, plasma membrane 1	down
ATP6V0A2	23545	ATPase, H+ transporting, lysosomal V0 subunit a2	down
ATP6V0E1	8992	ATPase, H+ transporting, lysosomal 9kDa, V0 subunit e1	up
ATP6V1C1	528	ATPase, H+ transporting, lysosomal 42kDa, V1 subunit C1	up
ATP8B4	79895	ATPase, class I, type 8B, member 4	up
ATP9A	10079	ATPase, class II, type 9A	up
ATXN7	6314	ataxin 7	down
AZI2	64343	5-azacytidine induced 2	up
BANP	54971	BTG3 associated nuclear protein	down
BATF	10538	basic leucine zipper transcription factor, ATF-like	up
BCAT1	586	branched chain amino-acid transaminase 1, cytosolic	up
BCL6	604	B-cell CLL/lymphoma 6	up
BCLAF1	9774	BCL2-associated transcription factor 1	down
BCOR	54880	BCL6 corepressor	down

BIK	638	BCL2-interacting killer (apoptosis-inducing)	up
BLOC1S1	2647	biogenesis of lysosomal organelles complex-1, subunit 1	up
BPI	671	bactericidal/permeability-increasing protein	up
BRD1	23774	bromodomain containing 1	down
BRI3	25798	brain protein I3	up
BTBD11	121551	BTB (POZ) domain containing 11	down
BTN2A2	10385	butyrophilin, subfamily 2, member A2	down
BTN3A1	11119	butyrophilin, subfamily 3, member A1	down
BTN3A2	11118	butyrophilin, subfamily 3, member A2	down
BUB3	9184	BUB3 mitotic checkpoint protein	down
BZW2	28969	basic leucine zipper and W2 domains 2	down
C11orf71	54494	chromosome 11 open reading frame 71	up
C16orf54	283897	chromosome 16 open reading frame 54	down
C19orf60	55049	chromosome 19 open reading frame 60	down
C19orf66	55337	chromosome 19 open reading frame 66	down
C1orf162	128346	chromosome 1 open reading frame 162	up
C1orf174	339448	chromosome 1 open reading frame 174	down
C1QA	712	complement component 1, q subcomponent, A chain	up
C2orf68	388969	chromosome 2 open reading frame 68	down
C3AR1	719	complement component 3a receptor 1	up
C5orf30	90355	chromosome 5 open reading frame 30	up
C8orf88	100127983	chromosome 8 open reading frame 88	up
CA4	762	carbonic anhydrase IV	up
CAB39	51719	calcium binding protein 39	up
CAMK1D	57118	calcium/calmodulin-dependent protein kinase ID	down
CAMKK2	10645	calcium/calmodulin-dependent protein kinase kinase 2, beta	up
CAPG	822	capping protein (actin filament), gelsolin-like	up
CAPN3	825	calpain 3	up
CARD6	84674	caspase recruitment domain family, member 6	up
CASS4	57091	Cas scaffolding protein family member 4	down
CAST	831	calpastatin	up
CBLL1	79872	Cbl proto-oncogene-like 1, E3 ubiquitin protein ligase	down
CBS	875	cystathionine-beta-synthase	up
CBX7	23492	chromobox homolog 7	down
CCDC71L	168455	coiled-coil domain containing 71-like	up

CCDC84	338657	coiled-coil domain containing 84	down
CCDC88C	440193	coiled-coil domain containing 88C	down
CCDC92	80212	coiled-coil domain containing 92	down
CCND3	896	cyclin D3	up
CCNL2	81669	cyclin L2	down
CCR3	1232	chemokine (C-C motif) receptor 3	down
CD163	9332	CD163 molecule	up
CD177	57126	CD177 molecule	up
CD4	920	CD4 molecule	down
CD47	961	CD47 molecule	down
CD55	1604	CD55 molecule, decay accelerating factor for complement (Cromer blood group)	up
CD63	967	CD63 molecule	up
CD82	3732	CD82 molecule	up
CDC14A	8556	cell division cycle 14A	down
CDC25A	993	cell division cycle 25A	up
CDC42EP3	10602	CDC42 effector protein (Rho GTPase binding) 3	up
CDC42SE2	56990	CDC42 small effector 2	down
CDIP1	29965	cell death-inducing p53 target 1	down
CDK5RAP2	55755	CDK5 regulatory subunit associated protein 2	up
CEBPA	1050	CCAAT/enhancer binding protein (C/EBP), alpha	up
CEP164	22897	centrosomal protein 164kDa	down
CFAP20	29105	cilia and flagella associated protein 20	down
CHPT1	56994	choline phosphotransferase 1	up
CIAO1	9391	cytosolic iron-sulfur assembly component 1	down
CIITA	4261	class II, major histocompatibility complex, transactivator	down
CKAP4	10970	cytoskeleton-associated protein 4	up
CLEC4D	338339	C-type lectin domain family 4, member D	up
CLIC1	1192	chloride intracellular channel 1	up
CNNM3	26505	cyclin and CBS domain divalent metal cation transport mediator 3	down
CNOT6L	246175	CCR4-NOT transcription complex subunit 6-like	down
CNOT7	29883	CCR4-NOT transcription complex subunit 7	down
COA1	55744	cytochrome c oxidase assembly factor 1 homolog	down
COLGALT1	79709	collagen beta(1-O)galactosyltransferase 1	up
COPS7B	64708	COP9 signalosome subunit 7B	down
CORO2A	7464	coronin, actin binding protein, 2A	up

CR1	1378	complement component (3b/4b) receptor 1 (Knops blood group)	up
CREB1	1385	cAMP responsive element binding protein 1	down
CREBL2	1389	cAMP responsive element binding protein-like 2	down
CRTAP	10491	cartilage associated protein	down
CSE1L	1434	CSE1 chromosome segregation 1-like (yeast)	down
CSGALNACT1	55790	chondroitin sulfate N-acetylgalactosaminyltransferase 1	up
CSGALNACT2	55454	chondroitin sulfate N-acetylgalactosaminyltransferase 2	up
CSNK1G2	1455	casein kinase 1, gamma 2	down
CST7	8530	cystatin F (leukocystatin)	up
CTDSPL2	51496	CTD small phosphatase like 2	down
CTSB	1508	cathepsin B	up
CTSC	1075	cathepsin C	down
CTSD	1509	cathepsin D	up
CWF19L2	143884	CWF19-like 2, cell cycle control (S. pombe)	down
CXorf40A	91966	chromosome X open reading frame 40A	down
CYB561	1534	cytochrome b561	down
CYLD	1540	cylindromatosis (turban tumor syndrome)	down
CYP1B1	1545	cytochrome P450, family 1, subfamily B, polypeptide 1	up
CYSLTR1	10800	cysteinyl leukotriene receptor 1	down
CYSTM1	84418	cysteine-rich transmembrane module containing 1	up
CYYR1	116159	cysteine/tyrosine-rich 1	up
DAAM2	23500	dishevelled associated activator of morphogenesis 2	up
DACH1	1602	dachshund family transcription factor 1	up
DBT	1629	dihydrolipoamide branched chain transacylase E2	down
DCAF16	54876	DDB1 and CUL4 associated factor 16	down
DCP1A	55802	decapping mRNA 1A	down
DCTN2	10540	dynactin 2 (p50)	up
DDAH2	23564	dimethylarginine dimethylaminohydrolase 2	up
DDIAS	220042	DNA damage-induced apoptosis suppressor	up
DDX17	10521	DEAD (Asp-Glu-Ala-Asp) box helicase 17	down
DDX18	8886	DEAD (Asp-Glu-Ala-Asp) box polypeptide 18	down
DDX24	57062	DEAD (Asp-Glu-Ala-Asp) box helicase 24	down
DDX27	55661	DEAD (Asp-Glu-Ala-Asp) box polypeptide 27	down
DDX5	1655	DEAD (Asp-Glu-Ala-Asp) box helicase 5	down
DDX50	79009	DEAD (Asp-Glu-Ala-Asp) box polypeptide 50	down

DHRS13	147015	dehydrogenase/reductase (SDR family) member 13	up
DIAPH2	1730	diaphanous-related formin 2	up
DIDO1	11083	death inducer-obliterator 1	down
DIRC2	84925	disrupted in renal carcinoma 2	up
DKC1	1736	dyskeratosis congenita 1, dyskerin	down
DLC1	10395	DLC1 Rho GTPase activating protein	up
DLG5	9231	discs, large homolog 5 (Drosophila)	down
DNAJC13	23317	DnaJ (Hsp40) homolog, subfamily C, member 13	up
DNAJC4	3338	DnaJ (Hsp40) homolog, subfamily C, member 4	up
DNAJC5	80331	DnaJ (Hsp40) homolog, subfamily C, member 5	up
DNASE1L1	1774	deoxyribonuclease I-like 1	up
DND1	373863	DND microRNA-mediated repression inhibitor 1	down
DNMT3A	1788	DNA (cytosine-5-)-methyltransferase 3 alpha	down
DOK3	79930	docking protein 3	up
DPY19L3	147991	dpy-19-like 3 (C. elegans)	up
DSC2	1824	desmocollin 2	up
DSE	29940	dermatan sulfate epimerase	up
DYRK2	8445	dual specificity tyrosine-(Y)-phosphorylation regulated kinase 2	down
DYSF	8291	dysferlin	up
ECHDC3	79746	enoyl-CoA hydratase domain containing 3	up
EDEM1	9695	ER degradation enhancer, mannosidase alpha-like 1	down
EFCAB14	9813	EF-hand calcium binding domain 14	down
EIF1B	10289	eukaryotic translation initiation factor 1B	up
EIF3A	8661	eukaryotic translation initiation factor 3, subunit A	down
EIF4A2	1974	eukaryotic translation initiation factor 4A2	down
EIF4E3	317649	eukaryotic translation initiation factor 4E family member 3	up
EIF5B	9669	eukaryotic translation initiation factor 5B	down
ELAVL1	1994	ELAV like RNA binding protein 1	down
EMILIN2	84034	elastin microfibril interfacer 2	up
ENO1	2023	enolase 1, (alpha)	up
EP400	57634	E1A binding protein p400	down
EPAS1	2034	endothelial PAS domain protein 1	up
ERCC1	2067	excision repair cross-complementation group 1	down
ERLIN2	11160	ER lipid raft associated 2	up
ERMAP	114625	erythroblast membrane-associated protein (Scianna blood group)	up

ESYT2	57488	extended synaptotagmin-like protein 2	down
ETS2	2114	v-ets avian erythroblastosis virus E26 oncogene homolog 2	up
EXOC6	54536	exocyst complex component 6	up
EXOSC4	54512	exosome component 4	up
EXT1	2131	exostosin glycosyltransferase 1	up
F5	2153	coagulation factor V (proaccelerin, labile factor)	up
FADS1	3992	fatty acid desaturase 1	up
FAM105A	54491	family with sequence similarity 105, member A	up
FAM110B	90362	family with sequence similarity 110, member B	up
FAM117B	150864	family with sequence similarity 117, member B	down
FAM118A	55007	family with sequence similarity 118, member A	down
FAM118B	79607	family with sequence similarity 118, member B	up
FAM160A2	84067	family with sequence similarity 160, member A2	up
FAM172A	83989	family with sequence similarity 172, member A	up
FAM200B	285550	family with sequence similarity 200, member B	up
FAM208B	54906	family with sequence similarity 208, member B	down
FAM20C	56975	family with sequence similarity 20, member C	up
FAM73A	374986	family with sequence similarity 73, member A	down
FAM89A	375061	family with sequence similarity 89, member A	up
FAN1	22909	FANCD2/FANCI-associated nuclease 1	down
FANCM	57697	Fanconi anemia complementation group M	down
FARP2	9855	FERM, ARH/RhoGEF and pleckstrin domain protein 2	down
FBXO38	81545	F-box protein 38	up
FBXO9	26268	F-box protein 9	up
FBXW2	26190	F-box and WD repeat domain containing 2	up
FCAR	2204	Fc fragment of IgA receptor	up
FCER1A	2205	Fc fragment of IgE, high affinity I, receptor for; alpha polypeptide	down
FCER1G	2207	Fc fragment of IgE, high affinity I, receptor for; gamma polypeptide	up
FCF1	51077	FCF1 rRNA-processing protein	down
FCMR	9214	Fc fragment of IgM receptor	down
FEM1C	56929	fem-1 homolog c (C. elegans)	up
FERMT3	83706	fermitin family member 3	up
FGFBP2	83888	fibroblast growth factor binding protein 2	down
FGFR1OP2	26127	FGFR1 oncogene partner 2	up
FGL2	10875	fibrinogen-like 2	down

FGR	2268	FGR proto-oncogene, Src family tyrosine kinase	up
FIP1L1	81608	factor interacting with PAPOLA and CPSF1	down
FKBP5	2289	FK506 binding protein 5	up
FKBP9	11328	FK506 binding protein 9	up
FLOT1	10211	flotillin 1	up
FLOT2	2319	flotillin 2	up
FLT3	2322	fms-related tyrosine kinase 3	up
FLT3LG	2323	fms-related tyrosine kinase 3 ligand	down
FLVCR1	28982	feline leukemia virus subgroup C cellular receptor 1	down
FMNL3	91010	formin like 3	down
FN3KRP	79672	fructosamine 3 kinase related protein	down
FOLR3	2352	folate receptor 3 (gamma)	up
FOXN2	3344	forkhead box N2	up
FOXN3	1112	forkhead box N3	down
FOXP1	27086	forkhead box P1	down
FUNDC1	139341	FUN14 domain containing 1	down
G3BP1	10146	GTPase activating protein (SH3 domain) binding protein 1	down
GADD45A	1647	growth arrest and DNA-damage-inducible, alpha	up
GALNT14	79623	polypeptide N-acetylgalactosaminyltransferase 14	up
GAPDH	2597	glyceraldehyde-3-phosphate dehydrogenase	up
GAS7	8522	growth arrest-specific 7	up
GATC	283459	glutamyl-tRNA(Gln) amidotransferase, subunit C	down
GBA	2629	glucosidase, beta, acid	up
GCC2	9648	GRIP and coiled-coil domain containing 2	down
GCNT1	2650	glucosaminyl (N-acetyl) transferase 1, core 2	up
GGH	8836	gamma-glutamyl hydrolase (conjugase, folylpolygammaglutamyl hydrolase)	up
GIMAP4	55303	GTPase, IMAP family member 4	down
GLDN	342035	gliomedin	up
GLTP	51228	glycolipid transfer protein	up
GNG5	2787	guanine nucleotide binding protein (G protein), gamma 5	up
GNG7	2788	guanine nucleotide binding protein (G protein), gamma 7	down
GNLY	10578	granulysin	down
GOLGA3	2802	golgin A3	down
GPATCH1	55094	G-patch domain containing 1	down
GPATCH11	253635	G-patch domain containing 11	down

GPATCH8	23131	G-patch domain containing 8	down
GPR155	151556	G protein-coupled receptor 155	down
GPR160	26996	G protein-coupled receptor 160	up
GPR183	1880	G protein-coupled receptor 183	down
GPR84	53831	G protein-coupled receptor 84	up
GRAMD1A	57655	GRAM domain containing 1A	up
GRAMD1C	54762	GRAM domain containing 1C	down
GRB10	2887	growth factor receptor bound protein 10	up
GRINA	2907	glutamate receptor, ionotropic, N-methyl D-aspartate-associated protein 1 (glutamate binding)	up
GRK5	2869	G protein-coupled receptor kinase 5	down
GSE1	23199	Gse1 coiled-coil protein	down
GTF3C2	2976	general transcription factor IIIC subunit 2	down
**GYG1**	**2992**	**glycogenin 1**	**up**
GZMA	3001	granzyme A	down
H2AFV	94239	H2A histone family, member V	down
H2AFY	9555	H2A histone family, member Y	up
H6PD	9563	hexose-6-phosphate dehydrogenase (glucose 1-dehydrogenase)	up
HACL1	26061	2-hydroxyacyl-CoA lyase 1	down
HAUS4	54930	HAUS augmin like complex subunit 4	up
HDAC9	9734	histone deacetylase 9	down
HES1	3280	hes family bHLH transcription factor 1	up
HGF	3082	hepatocyte growth factor (hepapoietin A; scatter factor)	up
HHEX	3087	hematopoietically expressed homeobox	up
HIPK2	28996	homeodomain interacting protein kinase 2	up
HIST1H2BD	3017	histone cluster 1, H2bd	up
HIST1H2BG	8339	histone cluster 1, H2bg	up
HIVEP2	3097	human immunodeficiency virus type I enhancer binding protein 2	down
HK2	3099	hexokinase 2	up
HK3	3101	hexokinase 3 (white cell)	up
HKR1	284459	HKR1, GLI-Kruppel zinc finger family member	down
HLA-DMA	3108	major histocompatibility complex, class II, DM alpha	down
HLA-DMB	3109	major histocompatibility complex, class II, DM beta	down
HLA-DOB	3112	major histocompatibility complex, class II, DO beta	down
HLA-DQB1	3119	major histocompatibility complex, class II, DQ beta 1	down
HLA-DRA	3122	major histocompatibility complex, class II, DR alpha	down

HMGB2	3148	high mobility group box 2	up
HMGN4	10473	high mobility group nucleosomal binding domain 4	down
HNRNPA0	10949	heterogeneous nuclear ribonucleoprotein A0	down
HNRNPA3	220988	heterogeneous nuclear ribonucleoprotein A3	down
HNRNPC	3183	heterogeneous nuclear ribonucleoprotein C (C1/C2)	down
HNRNPDL	9987	heterogeneous nuclear ribonucleoprotein D like	down
HNRNPH3	3189	heterogeneous nuclear ribonucleoprotein H3 (2H9)	down
HNRNPR	10236	heterogeneous nuclear ribonucleoprotein R	down
HNRNPU	3192	heterogeneous nuclear ribonucleoprotein U (scaffold attachment factor A)	down
HNRNPUL2	221092	heterogeneous nuclear ribonucleoprotein U-like 2	down
HP	3240	haptoglobin	up
HP1BP3	50809	heterochromatin protein 1, binding protein 3	down
HPGD	3248	hydroxyprostaglandin dehydrogenase 15-(NAD)	up
HRH2	3274	histamine receptor H2	up
HS1BP3	64342	HCLS1 binding protein 3	up
HSD3B7	80270	hydroxy-delta-5-steroid dehydrogenase, 3 beta- and steroid delta-isomerase 7	up
HSH2D	84941	hematopoietic SH2 domain containing	down
HSPA1A	3303	heat shock 70kDa protein 1A	up
HSPD1	3329	heat shock 60kDa protein 1 (chaperonin)	down
HVCN1	84329	hydrogen voltage gated channel 1	down
IDNK	414328	idnK, gluconokinase homolog (E. coli)	up
IER3	8870	immediate early response 3	up
IFIT1	3434	interferon-induced protein with tetratricopeptide repeats 1	down
IFNGR2	3460	interferon gamma receptor 2 (interferon gamma transducer 1)	up
IGFBP7	3490	insulin like growth factor binding protein 7	up
IL12RB1	3594	interleukin 12 receptor, beta 1	down
IL18R1	8809	interleukin 18 receptor 1	up
IL18RAP	8807	interleukin 18 receptor accessory protein	up
IL1R1	3554	interleukin 1 receptor, type I	up
IL1R2	7850	interleukin 1 receptor, type II	up
IL4R	3566	interleukin 4 receptor	up
IL7R	3575	interleukin 7 receptor	down
INHBA	3624	inhibin beta A	up
INPP4A	3631	inositol polyphosphate-4-phosphatase type I A	down
IPO11	51194	importin 11	up

IRAK3	11213	interleukin 1 receptor associated kinase 3	up
IRS2	8660	insulin receptor substrate 2	up
ISG20	3669	interferon stimulated exonuclease gene 20kDa	down
ITGA4	3676	integrin alpha 4	down
ITGA7	3679	integrin alpha 7	up
ITGAM	3684	integrin, alpha M (complement component 3 receptor 3 subunit)	up
ITPKB	3707	inositol-trisphosphate 3-kinase B	down
ITPKC	80271	inositol-trisphosphate 3-kinase C	up
ITPRIPL2	162073	inositol 1,4,5-trisphosphate receptor interacting protein-like 2	up
ITSN1	6453	intersectin 1	up
JAG1	182	jagged 1	up
JAK1	3716	Janus kinase 1	down
JAK2	3717	Janus kinase 2	up
KANSL2	54934	KAT8 regulatory NSL complex subunit 2	down
KCNE1	3753	potassium channel, voltage gated subfamily E regulatory beta subunit 1	up
KCTD21	283219	potassium channel tetramerization domain containing 21	up
KDM2B	84678	lysine (K)-specific demethylase 2B	down
KHDRBS1	10657	KH domain containing, RNA binding, signal transduction associated 1	down
KIAA0355	9710	KIAA0355	down
KIAA0930	23313	KIAA0930	up
KIAA1143	57456	KIAA1143	down
KIAA1429	25962	KIAA1429	down
KIAA1715	80856	KIAA1715	up
KIAA1958	158405	KIAA1958	up
KIF1B	23095	kinesin family member 1B	up
KIF3C	3797	kinesin family member 3C	up
KISS1R	84634	KISS1 receptor	down
KIZ	55857	kizuna centrosomal protein	down
KLF14	136259	Kruppel-like factor 14	up
KLF7	8609	Kruppel-like factor 7 (ubiquitous)	up
KLHL22	84861	kelch-like family member 22	down
KLRB1	3820	killer cell lectin-like receptor subfamily B, member 1	down
KLRD1	3824	killer cell lectin-like receptor subfamily D, member 1	down
KMT5A	387893	lysine (K)-specific methyltransferase 5A	up
KRBOX4	55634	KRAB box domain containing 4	down

KRR1	11103	KRR1, small subunit (SSU) processome component, homolog (yeast)	down
LAIR1	3903	leukocyte-associated immunoglobulin-like receptor 1	up
LAMTOR1	55004	late endosomal/lysosomal adaptor, MAPK and MTOR activator 1	up
LAMTOR5	10542	late endosomal/lysosomal adaptor, MAPK and MTOR activator 5	up
LAPTM4B	55353	lysosomal protein transmembrane 4 beta	up
LARS2	23395	leucyl-tRNA synthetase 2, mitochondrial	down
LATS2	26524	large tumor suppressor kinase 2	up
LDHA	3939	lactate dehydrogenase A	up
LDLR	3949	low density lipoprotein receptor	up
LEPROTL1	23484	leptin receptor overlapping transcript-like 1	down
LGALS1	3956	lectin, galactoside-binding, soluble, 1	up
LGALS8	3964	lectin, galactoside-binding, soluble, 8	up
LIG3	3980	ligase III, DNA, ATP-dependent	down
LILRA5	353514	leukocyte immunoglobulin-like receptor, subfamily A (with TM domain), member 5	up
LIN7A	8825	lin-7 homolog A (C. elegans)	up
LIPT1	51601	lipoyltransferase 1	down
LMNB1	4001	lamin B1	up
LOC100507507	100507507	uncharacterized LOC100507507	up
LOC728392	728392	uncharacterized LOC728392	down
LRG1	116844	leucine-rich alpha-2-glycoprotein 1	up
LRMP	4033	lymphoid-restricted membrane protein	down
LRPAP1	4043	LDL receptor related protein associated protein 1	up
LRRFIP2	9209	leucine rich repeat (in FLII) interacting protein 2	up
LRRN1	57633	leucine rich repeat neuronal 1	up
LTA4H	4048	leukotriene A4 hydrolase	up
LTB4R	1241	leukotriene B4 receptor	up
LUC7L3	51747	LUC7-like 3 pre-mRNA splicing factor	down
LYRM4	57128	LYR motif containing 4	down
LYRM7	90624	LYR motif containing 7	down
LYSMD2	256586	LysM, putative peptidoglycan-binding, domain containing 2	down
LZTFL1	54585	leucine zipper transcription factor like 1	down
MAFB	9935	v-maf avian musculoaponeurotic fibrosarcoma oncogene homolog B	up
MAFG	4097	v-maf avian musculoaponeurotic fibrosarcoma oncogene homolog G	up
MAML2	84441	mastermind-like transcriptional coactivator 2	down
MAN2A1	4124	mannosidase, alpha, class 2A, member 1	down

MAN2A2	4122	mannosidase, alpha, class 2A, member 2	up
MAOA	4128	monoamine oxidase A	up
MAP1LC3B	81631	microtubule-associated protein 1 light chain 3 beta	up
MAP2K6	5608	mitogen-activated protein kinase kinase 6	up
MAP7	9053	microtubule-associated protein 7	down
MAPK14	1432	mitogen-activated protein kinase 14	up
MAPK8	5599	mitogen-activated protein kinase 8	down
MAPKAPK2	9261	mitogen-activated protein kinase-activated protein kinase 2	up
MARC1	64757	mitochondrial amidoxime reducing component 1	up
MBD1	4152	methyl-CpG binding domain protein 1	down
MCEMP1	199675	mast cell-expressed membrane protein 1	up
MCTP1	79772	multiple C2 domains, transmembrane 1	up
MCTP2	55784	multiple C2 domains, transmembrane 2	up
MDM4	4194	MDM4, p53 regulator	down
MEF2A	4205	myocyte enhancer factor 2A	up
MEF2C	4208	myocyte enhancer factor 2C	down
MEGF6	1953	multiple EGF-like-domains 6	down
METRNL	284207	meteorin, glial cell differentiation regulator-like	up
METTL21A	151194	methyltransferase like 21A	down
METTL22	79091	methyltransferase like 22	up
METTL3	56339	methyltransferase like 3	down
METTL9	51108	methyltransferase like 9	up
MFN2	9927	mitofusin 2	up
MFNG	4242	MFNG O-fucosylpeptide 3-beta-N-acetylglucosaminyltransferase	down
MFSD12	126321	major facilitator superfamily domain containing 12	down
MFSD13A	79847	major facilitator superfamily domain containing 13A	up
MGEA5	10724	meningioma expressed antigen 5 (hyaluronidase)	down
MGST1	4257	microsomal glutathione S-transferase 1	up
MICAL1	64780	microtubule associated monooxygenase, calponin and LIM domain containing 1	up
MKNK1	8569	MAP kinase interacting serine/threonine kinase 1	up
MLX	6945	MLX, MAX dimerization protein	up
MMP8	4317	matrix metallopeptidase 8	up
MMP9	4318	matrix metallopeptidase 9	up
MPEG1	219972	macrophage expressed 1	down
MPHOSPH8	54737	M-phase phosphoprotein 8	down

MPP7	143098	membrane protein, palmitoylated 7	up
MPRIP	23164	myosin phosphatase Rho interacting protein	down
MRFAP1L1	114932	Morf4 family associated protein 1-like 1	down
MRPS25	64432	mitochondrial ribosomal protein S25	down
MS4A6A	64231	membrane-spanning 4-domains, subfamily A, member 6A	up
MSL3	10943	male-specific lethal 3 homolog (Drosophila)	up
MSTO1	55154	misato 1, mitochondrial distribution and morphology regulator	down
MTERF2	80298	mitochondrial transcription termination factor 2	down
MTERF4	130916	mitochondrial transcription termination factor 4	down
MTF1	4520	metal-regulatory transcription factor 1	up
MTHFD2	10797	methylenetetrahydrofolate dehydrogenase (NADP+ dependent) 2, methenyltetrahydrofolate cyclohydr	up
MTX1	4580	metaxin 1	up
MYCBP2	23077	MYC binding protein 2, E3 ubiquitin protein ligase	down
MYCL	4610	v-myc avian myelocytomatosis viral oncogene lung carcinoma derived homolog	down
MYD88	4615	myeloid differentiation primary response 88	up
MYL12A	10627	myosin light chain 12A	up
MYO10	4651	myosin X	up
MZF1	7593	myeloid zinc finger 1	down
N4BP2L1	90634	NEDD4 binding protein 2-like 1	down
NAA40	79829	N(alpha)-acetyltransferase 40, NatD catalytic subunit	down
NAIP	4671	NLR family, apoptosis inhibitory protein	up
NANS	54187	N-acetylneuraminic acid synthase	up
NAPRT	93100	nicotinate phosphoribosyltransferase	up
NATD1	256302	N-acetyltransferase domain containing 1	up
NCBP2	22916	nuclear cap binding protein subunit 2	down
NCL	4691	nucleolin	down
NDUFAF1	51103	NADH dehydrogenase (ubiquinone) complex I, assembly factor 1	up
NEK6	10783	NIMA-related kinase 6	up
NFATC2IP	84901	nuclear factor of activated T-cells, cytoplasmic, calcineurin-dependent 2 interacting protein	down
NFATC3	4775	nuclear factor of activated T-cells, cytoplasmic, calcineurin-dependent 3	down
NFE2	4778	nuclear factor, erythroid 2	up
NFIL3	4783	nuclear factor, interleukin 3 regulated	up
NFKBIA	4792	nuclear factor of kappa light polypeptide gene enhancer in B-cells inhibitor, alpha	up
NFKBIZ	64332	nuclear factor of kappa light polypeptide gene enhancer in B-cells inhibitor, zeta	up
NFX1	4799	nuclear transcription factor, X-box binding 1	down

NKTR	4820	natural killer cell triggering receptor	down
NLRC4	58484	NLR family, CARD domain containing 4	up
NLRP1	22861	NLR family, pyrin domain containing 1	down
NME8	51314	NME/NM23 family member 8	up
NMRK1	54981	nicotinamide riboside kinase 1	up
NONO	4841	non-POU domain containing, octamer-binding	down
NOP10	55505	NOP10 ribonucleoprotein	up
NOV	4856	nephroblastoma overexpressed	down
NQO2	4835	NAD(P)H dehydrogenase, quinone 2	up
NSUN7	79730	NOP2/Sun domain family, member 7	up
NT5DC1	221294	5'-nucleotidase domain containing 1	down
NUB1	51667	negative regulator of ubiquitin-like proteins 1	down
NUP160	23279	nucleoporin 160kDa	down
NUP93	9688	nucleoporin 93kDa	down
OGFRL1	79627	opioid growth factor receptor-like 1	down
OLAH	55301	oleoyl-ACP hydrolase	up
OPLAH	26873	5-oxoprolinase (ATP-hydrolysing)	up
ORM1	5004	orosomucoid 1	up
ORMDL3	94103	ORMDL sphingolipid biosynthesis regulator 3	down
OSCAR	126014	osteoclast associated, immunoglobulin-like receptor	up
OXSR1	9943	oxidative stress responsive 1	up
P2RY10	27334	purinergic receptor P2Y, G-protein coupled, 10	down
PAG1	55824	phosphoprotein membrane anchor with glycosphingolipid microdomains 1	up
PAM	5066	peptidylglycine alpha-amidating monooxygenase	up
PAPSS2	9060	3'-phosphoadenosine 5'-phosphosulfate synthase 2	up
PATZ1	23598	POZ (BTB) and AT hook containing zinc finger 1	down
PBXIP1	57326	pre-B-cell leukemia homeobox interacting protein 1	down
PCGF5	84333	polycomb group ring finger 5	down
PCMT1	5110	protein-L-isoaspartate (D-aspartate) O-methyltransferase	up
PCOLCE2	26577	procollagen C-endopeptidase enhancer 2	up
PCSK7	9159	proprotein convertase subtilisin/kexin type 7	down
PDCD4	27250	programmed cell death 4 (neoplastic transformation inhibitor)	down
PDE4B	5142	phosphodiesterase 4B, cAMP-specific	down
PDE4D	5144	phosphodiesterase 4D, cAMP-specific	up
PDGFC	56034	platelet derived growth factor C	up

PDLIM7	9260	PDZ and LIM domain 7 (enigma)	up
PECR	55825	peroxisomal trans-2-enoyl-CoA reductase	up
PER1	5187	period circadian clock 1	up
PFKFB2	5208	6-phosphofructo-2-kinase/fructose-2,6-biphosphatase 2	up
PFKFB3	5209	6-phosphofructo-2-kinase/fructose-2,6-biphosphatase 3	up
PGAP3	93210	post-GPI attachment to proteins 3	down
PGD	5226	phosphogluconate dehydrogenase	up
PGK1	5230	phosphoglycerate kinase 1	up
PGLYRP1	8993	peptidoglycan recognition protein 1	up
PGM1	5236	phosphoglucomutase 1	up
PGS1	9489	phosphatidylglycerophosphate synthase 1	up
PHACTR2	9749	phosphatase and actin regulator 2	up
PHB	5245	prohibitin	down
PHF1	5252	PHD finger protein 1	down
PHF20	51230	PHD finger protein 20	down
PHF21A	51317	PHD finger protein 21A	up
PHKA2	5256	phosphorylase kinase, alpha 2 (liver)	up
PHTF1	10745	putative homeodomain transcription factor 1	up
PI4KA	5297	phosphatidylinositol 4-kinase, catalytic, alpha	down
PIK3C3	5289	phosphatidylinositol 3-kinase, catalytic subunit type 3	down
PIK3CB	5291	phosphatidylinositol-4,5-bisphosphate 3-kinase, catalytic subunit beta	up
PIM2	11040	Pim-2 proto-oncogene, serine/threonine kinase	down
PIM3	415116	Pim-3 proto-oncogene, serine/threonine kinase	up
PITPNB	23760	phosphatidylinositol transfer protein, beta	down
PIWIL4	143689	piwi-like RNA-mediated gene silencing 4	up
PKM	5315	pyruvate kinase, muscle	up
PLA2G4A	5321	phospholipase A2, group IVA (cytosolic, calcium-dependent)	up
PLB1	151056	phospholipase B1	up
PLIN3	10226	perilipin 3	up
PLIN5	440503	perilipin 5	up
PLOD1	5351	procollagen-lysine, 2-oxoglutarate 5-dioxygenase 1	up
PLP2	5355	proteolipid protein 2 (colonic epithelium-enriched)	up
PNISR	25957	PNN-interacting serine/arginine-rich protein	down
PNPLA6	10908	patatin-like phospholipase domain containing 6	up
POLH	5429	polymerase (DNA directed), eta	down

POLR2D	5433	polymerase (RNA) II (DNA directed) polypeptide D	down
POR	5447	P450 (cytochrome) oxidoreductase	up
PPARD	5467	peroxisome proliferator-activated receptor delta	down
PPHLN1	51535	periphilin 1	down
PPP1R2	5504	protein phosphatase 1, regulatory (inhibitor) subunit 2	down
PPP1R3D	5509	protein phosphatase 1, regulatory subunit 3D	up
PPP1R3E	90673	protein phosphatase 1, regulatory subunit 3E	down
PPP1R8	5511	protein phosphatase 1, regulatory subunit 8	down
PPP2R5C	5527	protein phosphatase 2, regulatory subunit B', gamma	down
PPP6R2	9701	protein phosphatase 6, regulatory subunit 2	down
PPTC7	160760	PTC7 protein phosphatase homolog	down
PQLC1	80148	PQ loop repeat containing 1	up
PQLC3	130814	PQ loop repeat containing 3	down
PRCP	5547	prolylcarboxypeptidase	up
PRF1	5551	perforin 1 (pore forming protein)	down
PRKAA1	5562	protein kinase, AMP-activated, alpha 1 catalytic subunit	up
PRKACB	5567	protein kinase, cAMP-dependent, catalytic, beta	down
PRKAG2	51422	protein kinase, AMP-activated, gamma 2 non-catalytic subunit	down
PRKD2	25865	protein kinase D2	down
PRKX	5613	protein kinase, X-linked	down
PRPF40A	55660	PRP40 pre-mRNA processing factor 40 homolog A	down
PRR5L	79899	proline rich 5 like	down
PRRC2C	23215	proline-rich coiled-coil 2C	down
PRSS33	260429	protease, serine, 33	down
PRUNE2	158471	prune homolog 2 (Drosophila)	up
PSMG4	389362	proteasome (prosome, macropain) assembly chaperone 4	down
PTGDR2	11251	prostaglandin D2 receptor 2	down
PTGR1	22949	prostaglandin reductase 1	up
PTK2B	2185	protein tyrosine kinase 2 beta	up
PTMA	5757	prothymosin, alpha	down
PTTG1IP	754	pituitary tumor-transforming 1 interacting protein	up
PTX3	5806	pentraxin 3, long	up
PUM2	23369	pumilio RNA binding family member 2	down
PXK	54899	PX domain containing serine/threonine kinase	up
PYGL	5836	phosphorylase, glycogen, liver	up

QSOX1	5768	quiescin Q6 sulfhydryl oxidase 1	up
RAB20	55647	RAB20, member RAS oncogene family	up
RAB22A	57403	RAB22A, member RAS oncogene family	down
RAB27A	5873	RAB27A, member RAS oncogene family	up
RAB31	11031	RAB31, member RAS oncogene family	up
RAB32	10981	RAB32, member RAS oncogene family	up
RAB7A	7879	RAB7A, member RAS oncogene family	up
RABGAP1L	9910	RAB GTPase activating protein 1-like	down
RABGEF1	27342	RAB guanine nucleotide exchange factor (GEF) 1	up
RAD23B	5887	RAD23 homolog B, nucleotide excision repair protein	up
RALGAPA2	57186	Ral GTPase activating protein, alpha subunit 2 (catalytic)	up
RANBP9	10048	RAN binding protein 9	up
RARRES3	5920	retinoic acid receptor responder (tazarotene induced) 3	down
RASGEF1A	221002	RasGEF domain family member 1A	up
RASSF5	83593	Ras association (RalGDS/AF-6) domain family member 5	down
RBBP6	5930	retinoblastoma binding protein 6	down
RBM12	10137	RNA binding motif protein 12	down
RBM15	64783	RNA binding motif protein 15	down
RBM25	58517	RNA binding motif protein 25	down
RBM26	64062	RNA binding motif protein 26	down
RBM27	54439	RNA binding motif protein 27	down
RBM4	5936	RNA binding motif protein 4	down
RBM4B	83759	RNA binding motif protein 4B	down
RBM8A	9939	RNA binding motif protein 8A	down
RBMS1	5937	RNA binding motif, single stranded interacting protein 1	up
RECK	8434	reversion-inducing-cysteine-rich protein with kazal motifs	down
REPIN1	29803	replication initiator 1	down
RETN	56729	resistin	up
RFT1	91869	RFT1 homolog	up
RGL4	266747	ral guanine nucleotide dissociation stimulator-like 4	up
RGPD5	84220	RANBP2-like and GRIP domain containing 5	down
RHOBTB3	22836	Rho-related BTB domain containing 3	down
RHOU	58480	ras homolog family member U	up
RICTOR	253260	RPTOR independent companion of MTOR, complex 2	down
RIN3	79890	Ras and Rab interactor 3	up

RIOK3	8780	RIO kinase 3	up
RNASEH1	246243	ribonuclease H1	down
RNASEH2B	79621	ribonuclease H2, subunit B	down
RNF10	9921	ring finger protein 10	up
RNF4	6047	ring finger protein 4	down
ROGDI	79641	rogdi homolog	up
ROPN1L	83853	rhophilin associated tail protein 1-like	up
RPA1	6117	replication protein A1	down
RPAP3	79657	RNA polymerase II associated protein 3	down
RPS27A	6233	ribosomal protein S27a	down
RPS6KA5	9252	ribosomal protein S6 kinase, 90kDa, polypeptide 5	down
RRAGD	58528	Ras-related GTP binding D	up
RSBN1	54665	round spermatid basic protein 1	up
RTN4	57142	reticulon 4	up
RUNX2	860	runt-related transcription factor 2	down
S100A12	6283	S100 calcium binding protein A12	up
S100A6	6277	S100 calcium binding protein A6	up
S100A9	6280	S100 calcium binding protein A9	up
SAMSN1	64092	SAM domain, SH3 domain and nuclear localization signals 1	up
SAP30	8819	Sin3A associated protein 30kDa	up
SAP30L	79685	SAP30-like	up
SARAF	51669	store-operated calcium entry-associated regulatory factor	down
SART3	9733	squamous cell carcinoma antigen recognized by T-cells 3	down
SCAF11	9169	SR-related CTD-associated factor 11	down
SCPEP1	59342	serine carboxypeptidase 1	up
SDHC	6391	succinate dehydrogenase complex, subunit C, integral membrane protein, 15kDa	up
SENP6	26054	SUMO1/sentrin specific peptidase 6	down
SEPT9	10801	septin 9	down
SERF1A	8293	small EDRK-rich factor 1A (telomeric)	down
SERPINB1	1992	serpin peptidase inhibitor, clade B (ovalbumin), member 1	up
SERPINB10	5273	serpin peptidase inhibitor, clade B (ovalbumin), member 10	up
SERPINB8	5271	serpin peptidase inhibitor, clade B (ovalbumin), member 8	up
SESN2	83667	sestrin 2	up
SET	6418	SET nuclear proto-oncogene	down
SETD6	79918	SET domain containing 6	down

SFI1	9814	SFI1 centrin binding protein	down
SGK1	6446	serum/glucocorticoid regulated kinase 1	down
SGMS2	166929	sphingomyelin synthase 2	up
SGTB	54557	small glutamine-rich tetratricopeptide repeat (TPR)-containing, beta	up
SH3GLB1	51100	SH3-domain GRB2-like endophilin B1	up
SIGLEC5	8778	sialic acid binding Ig-like lectin 5	up
SIGLEC9	27180	sialic acid binding Ig-like lectin 9	up
SIPA1L2	57568	signal-induced proliferation-associated 1 like 2	up
SKIV2L2	23517	superkiller viralicidic activity 2-like 2 (S. cerevisiae)	down
SLC11A1	6556	solute carrier family 11 (proton-coupled divalent metal ion transporter), member 1	up
SLC16A3	9123	solute carrier family 16 (monocarboxylate transporter), member 3	up
SLC16A6	9120	solute carrier family 16, member 6	up
SLC1A3	6507	solute carrier family 1 (glial high affinity glutamate transporter), member 3	up
SLC22A4	6583	solute carrier family 22 (organic cation/zwitterion transporter), member 4	up
SLC23A2	9962	solute carrier family 23 (ascorbic acid transporter), member 2	down
SLC25A16	8034	solute carrier family 25 (mitochondrial carrier), member 16	down
SLC25A40	55972	solute carrier family 25, member 40	up
SLC26A8	116369	solute carrier family 26 (anion exchanger), member 8	up
SLC2A3	6515	solute carrier family 2 (facilitated glucose transporter), member 3	up
SLC2A4RG	56731	SLC2A4 regulator	down
SLC30A1	7779	solute carrier family 30 (zinc transporter), member 1	up
SLC36A1	206358	solute carrier family 36 (proton/amino acid symporter), member 1	up
SLC37A3	84255	solute carrier family 37, member 3	up
SLC39A8	64116	solute carrier family 39 (zinc transporter), member 8	up
SLC44A1	23446	solute carrier family 44 (choline transporter), member 1	up
SLC5A3	6526	solute carrier family 5 (sodium/myo-inositol cotransporter), member 3	down
SLC7A6	9057	solute carrier family 7 (amino acid transporter light chain, y+L system), member 6	down
SLC9A7	84679	solute carrier family 9, subfamily A (NHE7, cation proton antiporter 7), member 7	down
SLCO3A1	28232	solute carrier organic anion transporter family, member 3A1	up
SLX4IP	128710	SLX4 interacting protein	up
SMAGP	57228	small cell adhesion glycoprotein	down
SMARCC1	6599	SWI/SNF related, matrix associated, actin dependent regulator of chromatin, subfamily c, member 1	down
SMARCD3	6604	SWI/SNF related, matrix associated, actin dependent regulator of chromatin, subfamily d, member 3	up
SMPDL3A	10924	sphingomyelin phosphodiesterase, acid-like 3A	up
SMS	6611	spermine synthase	down

SMURF2	64750	SMAD specific E3 ubiquitin protein ligase 2	down
SNAPC5	10302	small nuclear RNA activating complex polypeptide 5	down
SNRNP200	23020	small nuclear ribonucleoprotein, U5 200kDa subunit	down
SNTB2	6645	syntrophin, beta 2 (dystrophin-associated protein A1, 59kDa, basic component 2)	up
SNX3	8724	sorting nexin 3	up
SOCS3	9021	suppressor of cytokine signaling 3	up
SON	6651	SON DNA binding protein	down
SP140	11262	SP140 nuclear body protein	down
SP140L	93349	SP140 nuclear body protein-like	down
SPCS2	9789	signal peptidase complex subunit 2	down
SPTLC2	9517	serine palmitoyltransferase, long chain base subunit 2	up
SQRDL	58472	sulfide quinone reductase-like (yeast)	up
SRPK1	6732	SRSF protein kinase 1	up
SRRM1	10250	serine/arginine repetitive matrix 1	down
SRRT	51593	serrate, RNA effector molecule	down
SRSF10	10772	serine/arginine-rich splicing factor 10	down
SRSF5	6430	serine/arginine-rich splicing factor 5	down
SSBP2	23635	single-stranded DNA binding protein 2	down
SSBP3	23648	single stranded DNA binding protein 3	down
SSH1	54434	slingshot protein phosphatase 1	up
SSPN	8082	sarcospan	down
SSR1	6745	signal sequence receptor, alpha	down
ST3GAL4	6484	ST3 beta-galactoside alpha-2,3-sialyltransferase 4	up
ST6GAL1	6480	ST6 beta-galactosamide alpha-2,6-sialyltranferase 1	down
ST6GALNAC3	256435	ST6 (alpha-N-acetyl-neuraminyl-2,3-beta-galactosyl-1,3)-N-acetylgalactosaminide alpha-2,6-sialyltra	up
STOM	2040	stomatin	up
STRAP	11171	serine/threonine kinase receptor associated protein	down
STS	412	steroid sulfatase (microsomal), isozyme S	up
SULT1B1	27284	sulfotransferase family 1B member 1	up
SUPT16H	11198	SPT16 homolog, facilitates chromatin remodeling subunit	down
SYN2	6854	synapsin II	up
SYNE1	23345	spectrin repeat containing, nuclear envelope 1	up
SYNE2	23224	spectrin repeat containing, nuclear envelope 2	down
SYNRG	11276	synergin, gamma	down
TAF1D	79101	TATA box binding protein associated factor 1D	down

TAF3	83860	TATA box binding protein associated factor 3	down
TAF4	6874	TAF4 RNA polymerase II, TATA box binding protein (TBP)-associated factor, 135kDa	down
TAGAP	117289	T-cell activation RhoGTPase activating protein	down
TARDBP	23435	TAR DNA binding protein	down
TBC1D8	11138	TBC1 domain family, member 8 (with GRAM domain)	up
TCAIM	285343	T cell activation inhibitor, mitochondrial	up
TCF20	6942	transcription factor 20 (AR1)	down
TCN1	6947	transcobalamin I (vitamin B12 binding protein, R binder family)	up
TCN2	6948	transcobalamin II	up
TCTN1	79600	tectonic family member 1	down
TDRD9	122402	tudor domain containing 9	up
TERF1	7013	telomeric repeat binding factor (NIMA-interacting) 1	down
TFB2M	64216	transcription factor B2, mitochondrial	down
TFDP1	7027	transcription factor Dp-1	up
TGFBR3	7049	transforming growth factor beta receptor III	down
TGIF2	60436	TGFB-induced factor homeobox 2	down
TGS1	96764	trimethylguanosine synthase 1	down
THADA	63892	thyroid adenoma associated	down
TIGD3	220359	tigger transposable element derived 3	down
TIMP2	7077	TIMP metallopeptidase inhibitor 2	up
TK2	7084	thymidine kinase 2, mitochondrial	up
TLR2	7097	toll-like receptor 2	up
**TLR5**	**7100**	**toll-like receptor 5**	up
TM2D3	80213	TM2 domain containing 3	down
TMCO3	55002	transmembrane and coiled-coil domains 3	up
TMED8	283578	transmembrane p24 trafficking protein family member 8	up
TMEM11	8834	transmembrane protein 11	up
TMEM110	375346	transmembrane protein 110	up
TMEM169	92691	transmembrane protein 169	up
TMEM185B	79134	transmembrane protein 185B	up
TMEM260	54916	transmembrane protein 260	up
TMEM33	55161	transmembrane protein 33	up
TMEM70	54968	transmembrane protein 70	up
TMLHE	55217	trimethyllysine hydroxylase, epsilon	up
TNKS	8658	tankyrase, TRF1-interacting ankyrin-related ADP-ribose polymerase	down

TNPO3	23534	transportin 3	up
TOLLIP	54472	toll interacting protein	up
TOMM20	9804	translocase of outer mitochondrial membrane 20 homolog (yeast)	down
TOP2B	7155	topoisomerase (DNA) II beta	down
TOR4A	54863	torsin family 4, member A	up
TP53	7157	tumor protein p53	down
TP53BP1	7158	tumor protein p53 binding protein 1	down
TP53I11	9537	tumor protein p53 inducible protein 11	up
TP53I3	9540	tumor protein p53 inducible protein 3	up
TPM4	7171	tropomyosin 4	up
TPR	7175	translocated promoter region, nuclear basket protein	down
TPST1	8460	tyrosylprotein sulfotransferase 1	up
TPST2	8459	tyrosylprotein sulfotransferase 2	up
TRA2A	29896	transformer 2 alpha homolog (Drosophila)	down
TRABD	80305	TraB domain containing	up
TRAF3IP2	10758	TRAF3 interacting protein 2	down
TRIM13	10206	tripartite motif containing 13	down
TRIQK	286144	triple QxxK/R motif containing	up
TRPM2	7226	transient receptor potential cation channel, subfamily M, member 2	up
TRPS1	7227	trichorhinophalangeal syndrome I	up
TSPAN13	27075	tetraspanin 13	down
TSPAN14	81619	tetraspanin 14	up
TSPO	706	translocator protein (18kDa)	up
TSPYL1	7259	TSPY-like 1	down
TTC17	55761	tetratricopeptide repeat domain 17	down
TTC22	55001	tetratricopeptide repeat domain 22	down
TUBB	203068	tubulin, beta class I	down
TWF1	5756	twinfilin actin binding protein 1	up
TXN	7295	thioredoxin	up
TXNDC16	57544	thioredoxin domain containing 16	down
TYW1	55253	tRNA-yW synthesizing protein 1 homolog (S. cerevisiae)	down
UBAP1	51271	ubiquitin associated protein 1	up
UBE2F	140739	ubiquitin-conjugating enzyme E2F (putative)	up
UBE2H	7328	ubiquitin conjugating enzyme E2H	up
UBE2J1	51465	ubiquitin-conjugating enzyme E2, J1	up

UGCG	7357	UDP-glucose ceramide glucosyltransferase	up
UNC119B	84747	unc-119 lipid binding chaperone B	down
UPP1	7378	uridine phosphorylase 1	up
USB1	79650	U6 snRNA biogenesis 1	up
USPL1	10208	ubiquitin specific peptidase like 1	down
UTP23	84294	UTP23, small subunit (SSU) processome component, homolog (yeast)	down
UTP6	55813	UTP6, small subunit (SSU) processome component, homolog (yeast)	down
VAMP2	6844	vesicle associated membrane protein 2	down
VAPA	9218	VAMP associated protein A	up
VAT1	10493	vesicle amine transport 1	up
VIM	7431	vimentin	up
VNN1	8876	vanin 1	up
VPS35	55737	VPS35 retromer complex component	up
VSTM1	284415	V-set and transmembrane domain containing 1	up
VWA5A	4013	von Willebrand factor A domain containing 5A	up
WBP11	51729	WW domain binding protein 11	down
WDR19	57728	WD repeat domain 19	down
WDR73	84942	WD repeat domain 73	down
WDR82	80335	WD repeat domain 82	down
WHSC1L1	54904	Wolf-Hirschhorn syndrome candidate 1-like 1	down
WIPF1	7456	WAS/WASL interacting protein family, member 1	down
WIPI1	55062	WD repeat domain, phosphoinositide interacting 1	up
WSB1	26118	WD repeat and SOCS box containing 1	up
XRCC5	7520	X-ray repair complementing defective repair in Chinese hamster cells 5 (double-strand-break rejoining	down
YIPF1	54432	Yip1 domain family member 1	up
YOD1	55432	YOD1 deubiquitinase	up
YPEL1	29799	yippee like 1	down
YTHDC1	91746	YTH domain containing 1	down
YWHAH	7533	tyrosine 3-monooxygenase/tryptophan 5-monooxygenase activation protein, eta	up
YY1	7528	YY1 transcription factor	down
ZAK	51776	sterile alpha motif and leucine zipper containing kinase AZK	up
ZBED6CL	113763	ZBED6 C-terminal like	down
ZBTB4	57659	zinc finger and BTB domain containing 4	down
ZBTB7B	51043	zinc finger and BTB domain containing 7B	up
ZCCHC7	84186	zinc finger, CCHC domain containing 7	down

ZDHHC19	131540	zinc finger, DHHC-type containing 19	up
ZDHHC3	51304	zinc finger, DHHC-type containing 3	up
ZEB1	6935	zinc finger E-box binding homeobox 1	down
ZEB2	9839	zinc finger E-box binding homeobox 2	up
ZFAND5	7763	zinc finger, AN1-type domain 5	up
ZFP36L2	678	ZFP36 ring finger protein-like 2	down
ZNF134	7693	zinc finger protein 134	down
ZNF14	7561	zinc finger protein 14	down
ZNF211	10520	zinc finger protein 211	down
ZNF224	7767	zinc finger protein 224	down
ZNF225	7768	zinc finger protein 225	down
ZNF226	7769	zinc finger protein 226	down
ZNF227	7770	zinc finger protein 227	down
ZNF263	10127	zinc finger protein 263	down
ZNF264	9422	zinc finger protein 264	down
ZNF274	10782	zinc finger protein 274	down
ZNF3	7551	zinc finger protein 3	down
ZNF319	57567	zinc finger protein 319	up
ZNF329	79673	zinc finger protein 329	down
ZNF33A	7581	zinc finger protein 33A	down
ZNF366	167465	zinc finger protein 366	up
ZNF383	163087	zinc finger protein 383	down
ZNF419	79744	zinc finger protein 419	down
ZNF438	220929	zinc finger protein 438	up
ZNF544	27300	zinc finger protein 544	down
ZNF559	84527	zinc finger protein 559	down
ZNF561	93134	zinc finger protein 561	down
ZNF606	80095	zinc finger protein 606	down
ZNF638	27332	zinc finger protein 638	down
ZNF75A	7627	zinc finger protein 75a	down
ZNF764	92595	zinc finger protein 764	down
ZNF766	90321	zinc finger protein 766	down
ZNF787	126208	zinc finger protein 787	up
ZNF791	163049	zinc finger protein 791	down
ZNF805	390980	zinc finger protein 805	down

ZNF830	91603	zinc finger protein 830	down
ZNF85	7639	zinc finger protein 85	down
ZNF91	7644	zinc finger protein 91	down
ZSCAN18	65982	zinc finger and SCAN domain containing 18	down
ZSCAN25	221785	zinc finger and SCAN domain containing 25	down

Mean Signal of Group Sepsis(GSE13904)	Mean Signal of Group Control(GSE13904)	Fold Change(GSE13904)	p-value(GSE13904)	FDR(GSE13904)
7.520664	8.13324	-1.528987	0.012112	0.009809
7.620739	7.115468	1.419389	0.003611	0.001706
7.6439125	7.2625665	1.3059495	0.0202425	0.0280525
6.5819255	5.378529833	2.445297	0.001162667	0.000744333
7.239641	6.291854	1.9298255	0.0000505	0
3.734191	4.225346	-1.40557	0.006592	0.001706
7.271232	6.679820667	1.507185333	0.005093667	0.006720667
7.896762	7.507011	1.310167	0.023305	0.036225
5.336329	6.393428	-2.080744	0.000138	0
5.684702	4.4210765	2.478821	0.0002495	0
6.870742	5.806455	2.1488735	0.0000675	0
8.9474075	7.0438195	3.8260195	0.0000185	0
5.230758	5.8793095	-1.574575	0.0002635	0
8.993976	6.899734	4.270018	0.00002	0
4.610588	3.693496	1.888305	0.002537	0.000703
6.910873	7.436938	-1.439996	0.000142	0
7.279742	6.094845	2.48286525	0.0009845	0.0004265
7.611589	7.147979	1.378988	0.000406	0
8.333074	7.074300333	2.407761	3.56667E-05	0
7.800992	6.449267	2.552172	0.000904	0
7.363533333	6.376634	2.020456333	0.000363333	0
7.198056	6.50801	1.613336	0.000567	0
10.00045967	9.453340667	1.463495667	0.013943667	0.020190333
6.431511	7.134919	-1.628347	0.000463	0
7.463927	5.251892	4.7121155	0.000029	0
6.833442	7.226969	-1.3136	0.007532	0.004466
6.18495	6.6645765	-1.394707	0.0162065	0.0148445
5.361446	4.6129	1.6801	0.010216	0.009809
6.379941	6.800062	-1.33804	0.004268	0.001706
9.013879	9.413874	-1.319504	0.006485	0.001706
7.045649	6.716949	1.255881	0.023409	0.036225
7.286696	7.728757	-1.358544	0.003763	0.000703
8.821449	9.724462	-1.869967	0.000289	0

6.737948	5.667419	2.100204	0.000019	0
8.612117	7.785103	1.7747525	0.004158	0.002233
10.166528	6.467896	12.983718	0.000018	0
6.9968825	7.4457395	-1.3670615	0.0036295	0.000994
7.1140375	6.416279	1.6229365	0.000382	0
8.639330286	7.735709714	1.893806571	0.000658857	0.000140714
6.256922	6.992877	-1.6655	0.002424	0.000703
5.761108	4.189005	5.763238333	0.001906667	0.000803
5.419693	4.89051475	1.45056325	0.00685525	0.006513
6.522263	7.167874	-1.564402	0.005286	0.001706
6.267492	6.7940185	-1.4405255	0.0029055	0.000853
7.648819	6.797779	1.8038	0.001864	0.000703
7.55887	7.072233	1.401176	0.019263	0.036225
10.570718	9.975982	1.510195	0.002903	0.001706
5.985170833	5.508527333	1.4016075	0.006005667	0.005563833
9.969848	9.4938935	1.39518375	0.01870675	0.0280525
5.486566375	4.318557625	2.463023	0.00044175	0.000087875
6.48737	6.891881333	-1.324481	0.015682	0.013347333
7.26186	6.721911	1.453921	0.001379	0.000282
6.70439	5.6517516	2.1094208	0.0001268	0
5.514812	5.067909	1.363111	0.017775	0.01988
6.945722	7.880671	-1.911981	0.0004755	0
4.531184	5.0590685	-1.454635	0.003073	0.000853
8.2282498	7.5639114	1.5984184	0.0008032	0.000197
7.070369	6.03806125	2.11801925	0.00409325	0.00497
5.61651	4.484531	2.191592	0.00068	0
6.133439	5.376529	1.7211265	0.0016285	0.0004925
5.84146	6.137999	-1.228195	0.016967	0.009809
5.802323333	5.246503667	1.475975	0.009639333	0.013798
8.1690655	8.5814115	-1.331938	0.010179	0.005256
8.100595	7.64195	1.374251	0.000469	0
5.8498985	4.69939325	2.44682725	0.0030345	0.00287875
9.50718525	7.907586	3.24148675	0.000577	0.00017575
6.772294333	7.539430667	-1.714274	0.000466333	0
4.843904	5.3565265	-1.438713	0.0158325	0.0181125

6.385013	5.840384	1.458644	0.008983	0.009809
9.048426	8.337797	1.636518	0.00043	0
7.7008	5.455984	4.739767	0.000019	0
5.6873185	6.219293	-1.4459495	0.007214	0.003086
8.9414305	8.275339	1.612603	0.000767	0.000141
4.495154	5.1722345	-1.6308725	0.009601	0.00994
6.523962	7.111037	-1.502198	0.002718	0.000703
7.5956725	8.232187	-1.5822375	0.014105	0.0181125
7.115533	7.7455875	-1.547765	0.0276745	0.023017
7.63439975	8.58213775	-1.96495275	0.00334075	0.00129225
7.955849	8.77498	-1.764343	0.000275	0
6.788807	6.523957	1.201511	0.020217	0.036225
9.902149	10.238672	-1.262709	0.031078	0.036225
6.5719505	6.909121	-1.263765	0.0233325	0.01988
7.516306	7.9238635	-1.328692	0.0101235	0.0071375
9.880911	9.174967	1.631212	0.004326	0.001706
7.152984	7.704386	-1.465509	0.007947	0.004466
6.634675	5.06523	2.967906	0.00011	0
6.93813	7.349712	-1.330143	0.013178	0.009809
8.935739	7.106402	3.553738	0.000026	0
6.570211	5.751657	1.763637	0.011468	0.009809
5.862968	4.89472	1.991704	0.000139	0
7.700933	5.5958405	4.652427	0.0000235	0
6.931397	5.96547	1.953317	0.008006	0.009809
7.476265	7.928647	-1.368298	0.024987	0.01988
7.806831	6.730469667	2.109560333	0.000227667	0
8.865475	7.84256	2.03202	0.000063	0
5.764804667	5.166282667	1.517476333	0.007354	0.008684
8.938577	7.316699	3.077756	0.000019	0
4.998889	5.572661	-1.48841	0.035687	0.036225
8.720274	8.052988	1.588148	0.001675	0.0004925
6.004664	6.714236	-1.635318	0.00235	0.000703
5.185406	4.632629	1.466907	0.001488	0.000282
7.911667	8.476752	-1.479475	0.002604	0.000703
7.590283	6.758585	1.779779	0.000135	0

6.148274	6.812282	-1.584478	0.000536	0
5.38879	6.092907	-1.670708	0.006304	0.0049045
6.553435	7.180223	-1.544124	0.000682	0
10.987742	10.576943	1.329422	0.012035	0.01988
7.812029	8.482059	-1.591106	0.000362	0
5.599301	7.016176	-2.670065	0.000215	0
7.454284333	5.548439333	3.804944667	0.000567333	0.000094
10.34229	6.011257	20.126612	0.000018	0
7.713811	8.160146	-1.362575	0.024551	0.01988
7.682118333	8.421927	-1.759317	0.008082333	0.006861
10.52411233	9.336753333	2.327815667	0.00173	0.001488667
10.735264	9.366746	2.582051	0.00002	0
7.6241505	7.0670355	1.471378	0.001845	0.000703
3.960795	4.951247	-1.986807	0.000116	0
2.84535	2.55443	1.223421	0.023675	0.036225
9.287026	8.0021395	2.4451765	0.000052	0
10.32713	11.270569	-1.923107	0.000037	0
5.817304	6.233152	-1.3341005	0.0014465	0.000141
6.966486	5.731315	2.383168	0.000108	0
8.201105	6.978096	2.334331	0.00002	0
5.32022	5.630155	-1.239652	0.015926	0.009809
7.19954	7.868416	-1.589833	0.001347	0.000282
7.436547667	6.515325333	1.943278333	0.002013667	0.000662667
6.131618	6.535014	-1.322618	0.014158	0.009809
5.760128	6.330803	-1.485218	0.00362	0.000703
7.710004667	6.264840333	3.130885333	0.003397333	0.003269667
8.3152215	5.9800035	5.073979	0.000019	0
11.339255	10.801011	1.452205	0.00094	0
3.060761	3.647028	-1.501358	0.000093	0
7.430166667	8.243421	-1.793152333	0.009908	0.012075
9.5801025	10.2978635	-1.6760245	0.007001	0.0049045
4.49378	4.903862	-1.328803	0.007303	0.0025845
7.295857	6.4575895	1.788594	0.0000625	0
5.831035	6.172723	-1.267238	0.01371	0.009809
5.641472	4.9216325	1.650123	0.001078	0.000141

7.760894833	6.414688333	2.670978167	0.002973	0.003313333
8.13248	8.607318	-1.395232	0.0123635	0.00994
7.561451	8.4658765	-1.8947745	0.000238	0
8.923251	9.300122	-1.298522	0.039226	0.036225
6.802329	7.473903667	-1.596436333	0.005944667	0.002291667
4.965652	4.495851	1.384918	0.025442	0.036225
7.847310333	7.075275667	1.708905333	0.013373667	0.019270333
8.405573	8.800187	-1.314591	0.005503	0.001706
11.092035	9.182275	3.757466	0.000022	0
6.267022667	7.103684667	-1.792582333	0.001113667	0.000234333
9.680058	8.8700804	1.7707	0.0004834	0
5.27316	5.688244	-1.333376	0.002768	0.000703
7.4653	6.243428	2.332491	0.000048	0
5.6702335	6.362153	-1.6154535	0.0008345	0
7.151016	7.502677	-1.276029	0.012907	0.010793
5.378126	5.807291	-1.3464725	0.0188465	0.0148445
6.739988	7.3422865	-1.555579167	0.011749667	0.009987
7.96309725	5.98990175	4.4182015	0.00202975	0.00245225
6.097971	6.74364	-1.564465	0.005358	0.001706
10.866394	8.54021	5.014769	0.000019	0
4.124501	3.615434	1.42313	0.0076355	0.0071375
6.765687	5.263736	2.832253	0.000363	0
6.361475667	4.839728	2.880038667	7.83333E-05	0
4.041406	4.540939	-1.413756	0.00137	0.000282
4.720407	5.603639	-1.844502	0.003082	0.000703
7.192054	7.534587	-1.267981	0.017536	0.009809
8.356255	8.085171	1.206714	0.018881	0.036225
7.539632	6.758775667	1.721602333	0.000864	0.000234333
6.812792	5.809391	2.00472	0.00009	0
9.8292075	10.8379815	-2.1786005	0.000389	0
6.9266095	7.626085	-1.644586	0.00296475	0.00067275
7.0903165	7.626048	-1.4564035	0.0042505	0.002233
7.350164	7.683864	-1.261327	0.011354333	0.008209333
8.55788	9.2168885	-1.5795645	0.0031495	0.000994
8.082286	8.924017	-1.792199	0.000234	0

7.914965	6.871102	2.061741	0.000025	0
6.782835	5.999051	1.721641	0.000404	0
5.97612575	6.33536825	-1.28632825	0.0151345	0.01396075
6.380647	5.3246885	2.0827635	0.0000435	0
6.9139515	7.5059675	-1.5079285	0.0014545	0.000141
4.684002	4.04683	1.585122	0.003516	0.002233
3.211302	3.746485	-1.449125	0.0003	0
6.859544	6.046123	1.757374	0.001449	0.000282
7.036811	6.5101235	1.4406365	0.010251	0.009809
7.39158	6.648069667	1.677294667	0.000312667	0
8.187621	7.413852	1.70973	0.000175	0
7.462305	7.79646	-1.260638	0.013642	0.009809
6.2941465	6.668448	-1.298923	0.0124415	0.00994
8.635575	7.4566075	2.3581385	0.0000455	0
7.429391	6.394642	2.048758	0.00072	0
6.84583375	5.54187525	2.56074275	0.00077875	0.00017575
8.332978	7.410653	1.895167	0.000268	0
6.51375125	7.74222475	-2.386052	0.0028445	0.0011165
10.150101	7.811751	5.05724	0.000018	0
7.385988	5.974498	2.660118	0.000071	0
7.915863	8.268808	-1.277165	0.008404	0.004466
8.953132	9.536563	-1.498408	0.000389	0
10.779715	10.305645	1.389023	0.006731	0.004466
6.9457265	7.5859065	-1.5625985	0.0083615	0.0049045
10.048133	10.993588	-1.925797	0.000501	0
8.218133667	7.111624667	2.157086333	0.000200333	0
5.17383625	5.65421525	-1.40053075	0.016393	0.016549
8.539643	8.921271	-1.30281	0.032002	0.036225
6.406550667	5.429565667	2.07302	0.002060333	0.001488667
8.889389	7.983462	1.937216	0.0070995	0.010081
5.9896185	6.4083345	-1.341551	0.002993	0.000853
5.818139	5.049006	1.704245	0.000815	0
5.734853	6.583294	-1.800554	0.000074	0
4.774431	4.264914	1.423573	0.004896	0.004466
5.44534	4.979203	1.381405	0.007084	0.004466

6.552370667	7.215235333	-1.604704667	0.013782333	0.009896333
6.077989	5.183702667	1.924874333	0.000362	0
6.261566667	5.426539333	1.804863667	0.001720667	0.000568667
7.322253	6.547111	1.730995333	0.00541	0.006626667
4.880047333	4.254180667	1.565982	0.013719667	0.018701667
7.087560667	5.616568333	2.809587	0.000366	0.000094
5.115616	4.605841	1.427384	0.001728	0.0004925
6.099914	5.432569333	1.588322667	0.006405667	0.006539333
4.8906905	4.3147825	1.493452	0.0007315	0.000141
7.504364	8.710318	-2.306899	0.000122	0
5.62663	5.988512	-1.2851	0.011656	0.009809
4.532813	4.210063	1.250712	0.008458	0.009809
7.400241	6.577331	1.76897	0.000031	0
5.089048	4.680052	1.327761	0.012661	0.01988
7.054721	6.458125667	1.514808333	0.00799	0.006633333
6.284448	6.928885	-1.563129	0.017996	0.01988
5.207814	4.769865	1.354677	0.007284	0.004466
3.945019	4.735211	-1.729305	0.000098	0
6.890709	5.787562	2.148228	0.000276	0
5.332445	5.955971	-1.540635	0.000501	0
4.4047915	4.7448135	-1.2662585	0.019172	0.0203455
6.15774	6.590149	-1.349485	0.000188	0
4.315734	3.596736	1.646038	0.005774	0.004466
6.923821333	6.101320667	1.807323333	0.002944	0.003269667
4.007518	3.631374	1.297868	0.001637	0.000282
6.7671578	5.024488	3.736636	0.0000484	0
4.425142	7.888567	-11.030491	0.000019	0
11.55346	9.882674	3.1924705	0.000019	0
6.857129	7.432726	-1.490294	0.004092	0.000703
9.1689065	10.276105	-2.1646415	0.000394	0
7.007478	6.261447	1.677172	0.000839	0
8.846381	8.236683	1.52594	0.00218	0.000703
5.392589	7.946174	-5.870911	0.000019	0
6.868146667	6.032615667	1.785138333	0.00785	0.009990333
10.29339	10.874752	-1.496261	0.016516	0.009809

10.419058	9.235027	2.272108	0.000028	0
5.206698	5.901673	-1.618855	0.001111	0
9.223078667	7.410564	3.530244667	0.000117	0
5.626464	4.868595	1.690991	0.000099	0
8.406373	7.124297	2.5490415	0.00003225	0
9.083262	7.4786045	3.1105425	0.0000205	0
5.924648	5.143498	1.718499	0.002009	0.000703
5.6674165	6.679986	-2.057604	0.0027475	0.000853
4.8307695	5.6165645	-1.764507	0.009956	0.00994
4.786144	5.389751667	-1.526381	0.001721333	0.000568667
6.064128	6.46283	-1.318321	0.016147	0.009809
8.45938	6.791099	3.178357	0.00044	0
3.907865	3.30092	1.523031	0.000059	0
8.175655	8.569477	-1.31387	0.00508	0.001706
7.7339218	8.3584362	-1.5531386	0.0131718	0.0113616
4.552959	5.548749	-1.994171	0.000597	0
7.7055325	8.386899	-1.60639	0.0047685	0.002233
8.502615	6.651212	3.60851	0.000034	0
7.171899	5.658164	2.855482	0.000034	0
12.30209617	11.62423183	1.604544833	0.000973167	0.000141
8.4829378	6.6628838	3.5985112	0.0000188	0
6.439632	6.923183	-1.398181	0.00434	0.001706
7.108365	6.146772	1.947459	0.000032	0
7.255505	8.323222	-2.096113	0.000043	0
5.532439	5.221896	1.240175	0.005262	0.004466
5.109432	4.47096	1.55668	0.016023	0.01988
9.504319	10.081397	-1.491825	0.0041	0.001706
4.499296	4.072281	1.344449	0.017784	0.01988
7.283959	6.660813	1.54023	0.001889	0.000703
11.147812	10.343146	1.746742	0.000087	0
6.492844	6.77394	-1.215117	0.020064	0.01988
7.7975215	10.153928	-5.124914	0.0000195	0
6.879064	7.232894	-1.277949	0.012036	0.009809
4.952634	5.37264	-1.337934	0.013497	0.009809
5.351906	6.220125	-1.825408	0.000087	0

7.033072	7.405828	-1.294824	0.005243	0.001706
6.493779	7.010842	-1.431039	0.0208	0.01988
8.749466	7.775812	1.963808	0.001027	0.000282
7.276368	9.229668	-3.872595	0.000018	0
7.898117	5.053614	7.182584	0.000019	0
7.373332	6.5248435	1.8010515	0.0002185	0
3.451393	4.305997	-1.808263	0.000418	0
6.48548075	4.9603115	3.00578275	0.000049	0
8.423425	7.566163	1.811597	0.000229	0
6.439205	7.021435	-1.497162	0.008809	0.004466
6.134539	6.743614	-1.527048	0.0047565	0.002233
7.6982075	8.027771	-1.256816	0.0129855	0.012173
**10.605027**	**8.186451**	**5.35319**	**0.0000185**	**0**
7.175937	9.324389	-4.433518	0.000025	0
7.005544667	7.496774667	-1.416032333	0.011618333	0.012169
8.5476005	7.858899	1.624256	0.0054315	0.00633275
6.964501	6.361164	1.519226	0.000463	0
6.317603	6.999498	-1.604246	0.0002	0
8.777087	7.649067	2.185585	0.000044	0
5.5443275	6.2555735	-1.6520185	0.000769	0.000141
3.401446	3.133698	1.203927	0.010363	0.009809
5.0372438	4.0367484	2.1444766	0.0010318	0.0003976
8.348748	7.799138	1.46369	0.015705	0.01988
6.779896333	6.199037333	1.502048333	0.014327667	0.019270333
8.5306745	7.3773085	2.2361985	0.0002835	0
5.9318575	5.1886235	1.6877815	0.0100455	0.010793
5.9550215	6.535058	-1.496483	0.0037825	0.000994
7.572717	6.616088	1.94077	0.000029	0
9.903487	7.722515	4.53459	0.000018	0
5.541707	6.052624	-1.424956	0.001774	0.000282
8.444853	9.259117	-1.7584	0.001432	0.000282
7.596509	8.547314	-1.93295	0.000119	0
7.699412	8.504015	-1.746665	0.000854	0
7.9668005	8.7541255	-1.725938	0.0039505	0.0012045
10.9558705	11.7024215	-1.677925	0.0017575	0.000282

9.765716	9.048275	1.644263	0.020025	0.036225
8.998420667	9.804115667	-1.748027	0.000102667	0
6.498581667	7.043533667	-1.473337	0.007264667	0.006626667
7.64628	8.4712705	-1.828651	0.005186	0.002233
7.9609735	8.476869	-1.440955	0.0116695	0.00994
7.790585333	8.560487	-1.712037667	0.001856333	0.000568667
7.6842445	8.210233	-1.440886	0.024691	0.0280525
7.156091	7.8634395	-1.6328265	0.0006345	0
5.0409245	5.546588	-1.427181	0.015426	0.0182535
7.028638	7.43267	-1.3232	0.020936	0.01988
8.5866705	5.7632815	7.0917595	0.000019	0
9.4496195	10.1245425	-1.6004185	0.000173	0
6.681094333	5.428537	2.415025	0.016381667	0.027419667
8.490069	6.567562	3.790811	0.000018	0
7.457991	7.121072	1.263056	0.002938	0.001706
6.977975	6.639517	1.264405	0.015195	0.01988
5.313594	5.684612	-1.293265	0.014445	0.009809
8.764342667	7.455189333	2.495113333	6.36667E-05	0
8.842259	9.547716	-1.630661	0.004465	0.001706
8.72726	9.378393	-1.570401	0.000196	0
6.82851	6.280415	1.462154	0.019401	0.036225
8.378362	7.524034	1.807917	0.000528	0
7.424151	8.951137	-2.881831	0.0356	0.036225
10.459623	9.730436	1.657705	0.000147	0
7.625631	7.083503	1.456119	0.016924	0.01988
6.945572	7.579405	-1.551682	0.007193	0.004466
7.826299	5.648125	4.525802	0.000102	0
10.765873	8.855389	3.759352	0.000052	0
7.534709	5.708454	3.546155	0.000044	0
10.8145505	7.514274	10.079321	0.000019	0
8.588455	7.878849	1.635357	0.000971	0.000282
9.8703215	11.533384	-3.218651	0.000082	0
3.258986	2.874375	1.305508	0.011693	0.009809
7.333908	7.9543045	-1.544218	0.0019255	0.000282
6.9846625	5.913391	2.368761	0.003966	0.0049045

7.879101	5.578899333	4.941798667	1.86667E-05	0
9.0374885	8.226433	1.761621	0.001661	0.000853
9.075986	9.516696	-1.357272	0.037001	0.036225
6.72795575	7.99150425	-2.4123775	0.00179575	0.0004265
5.670195	4.906151	1.698245	0.00078	0
10.051182	8.552634	2.825584	0.000019	0
6.804884	7.248888	-1.3634105	0.0036415	0.000994
6.446394	5.92209	1.438239	0.001375	0.000282
4.979021667	4.324554333	1.591144667	0.001372	0.000568667
4.2947645	3.9274295	1.2900015	0.015061	0.0203455
5.2131265	4.368763	1.795619	0.000608	0.000141
10.222919	10.665502	-1.359036	0.001518	0.000282
6.710038	5.787606	1.909889	0.002721	0.001488667
6.755878	7.441471	-1.608363	0.000758	0
4.778589	2.93687	3.584367	0.000107	0
6.188028	5.694685	1.407703	0.004738	0.001706
6.906397	7.73426	-1.775055	0.000116	0
9.393018	9.939728	-1.460751	0.00166	0.000282
5.441014	6.133964	-1.616585	0.000798	0
7.587379667	6.658609	1.907587333	0.00007	0
7.0046	7.618342	-1.5678765	0.0103425	0.00994
3.292194	3.728053	-1.352716	0.00403	0.000703
8.10218	7.472103	1.547648	0.000765	0
6.4584005	5.86753	1.506586	0.0080635	0.010081
7.707062667	5.832825667	3.698894667	0.000018	0
6.345202	5.86973	1.3981905	0.005849	0.0049045
4.275442	4.743439	-1.383187	0.005742	0.001706
6.602712	7.225431	-1.539775	0.001875	0.000282
4.645834	4.27613	1.292087	0.022381	0.036225
6.7782335	5.99676375	1.78079825	0.00156325	0.00060225
6.0954065	6.515235	-1.3377775	0.0058135	0.002233
7.137273	9.588399	-5.46843	0.000018	0
6.018925	7.559691667	-2.994199667	2.66667E-05	0
6.2985565	5.89697475	1.3227125	0.00490975	0.00283525
4.1231115	4.6498065	-1.4542055	0.001383	0.000141

5.405648	6.0823	-1.601057	0.0099185	0.004466
7.6877685	7.127457	1.475101	0.011806	0.012173
9.403859	8.898615	1.419363	0.00084	0
9.707353	9.14799	1.4736255	0.0022555	0.000703
5.213123667	4.818949333	1.314218667	0.014480333	0.016833333
4.855366	5.280302	-1.342513	0.020077	0.01988
8.0480235	7.135723	1.892688	0.0002675	0
11.426289	10.835757	1.505802	0.010895	0.009809
5.8174675	5.3044805	1.432031	0.006789	0.0071375
8.067987	8.9647225	-1.91627	0.0045575	0.002233
11.033294	10.362318	1.59215	0.00867	0.009809
6.593355333	5.805363333	1.738384667	0.001618667	0.000328333
5.5495145	5.9121705	-1.285851	0.0198375	0.0148445
7.986998	6.390825333	3.505221667	0.003559667	0.003269667
5.74479775	4.3463005	2.6496125	0.00009775	0
5.564643	6.671202	-2.153315	0.000045	0
7.903306	6.196401	3.264596	0.000018	0
6.853842	6.171979	1.6477515	0.005952	0.0049045
7.397059	7.983244	-1.501271	0.003183	0.000703
8.857428	6.779061	4.223288	0.000019	0
8.996309	9.415355	-1.337043	0.031563	0.036225
8.643216	7.82333	1.765266	0.000147	0
5.58457175	5.02654625	1.48385275	0.0059285	0.009232
4.135517	2.738229	2.63406	0.0001	0
11.211436	10.465877	1.676624	0.000687	0
7.309250333	5.798013333	2.928298333	0.000024	0
7.1823626	7.9436454	-1.7276758	0.0019782	0.0004818
5.848303	6.309151	-1.37848	0.004129	0.000994
6.362167667	7.236127	-1.859327667	0.001739667	0.000568667
9.071557	9.803902	-1.661337	0.003832	0.000703
4.8086485	5.5583	-1.686193	0.0007775	0
8.8564295	7.98293	1.8335165	0.006806	0.00994
6.763159	6.111876	1.6257975	0.0080535	0.00994
8.001358	8.779136	-1.714489	0.010398	0.004466
7.72382	8.574717	-1.803622	0.000205	0

7.4088905	6.713886	1.620039	0.0012825	0.000282
5.654044	4.708287	1.926199	0.001682	0.000703
9.5898295	8.7810685	1.766299	0.0003005	0
6.0253505	4.7780675	2.4677575	0.000109	0
3.754595	4.260204	-1.419723	0.01708	0.009809
9.236562667	7.307818	3.888326	0.000019	0
5.695877	6.311358333	-1.538886	0.000952	0.000094
6.942668333	6.348865667	1.553694	0.006223333	0.007195333
8.670713	7.326145	2.539542	0.000028	0
7.494462	7.818635	-1.251946	0.02964	0.036225
11.203763	7.449975	13.489719	0.000018	0
7.597253	6.395658	2.302229333	0.000258667	0
7.6597412	6.3628708	2.5376372	0.0000494	0
8.580357	9.373668	-1.733048	0.0001	0
6.85430475	6.06199	1.78007825	0.0057725	0.0093025
7.288106333	8.161444667	-1.850001333	0.003443667	0.001488667
5.606397	6.0318545	-1.344649	0.0128725	0.010793
7.210284	6.538925	1.592572	0.001121	0.000282
4.3258025	4.980812	-1.575113	0.004809	0.002233
6.763389	6.35595	1.326329	0.003428	0.001706
6.4037535	7.0184635	-1.5312755	0.001374	0.0003515
8.833094667	7.628322333	2.320801667	0.000072	0
6.9593145	6.3614575	1.513573	0.0019225	0.000853
8.449123	9.173071	-1.651696	0.000619	0
6.371595	6.959381	-1.502938	0.000624	0
5.891912	5.230066	1.582105	0.000186	0
9.345457	10.022104	-1.59842	0.001269	0.000282
7.616433	6.15993925	2.82886075	0.0000305	0
8.068306	7.449509	1.535594	0.003062	0.001706
8.247409333	6.700313	3.002957667	0.000029	0
8.047827	7.667049	1.30580575	0.012161	0.01625925
7.971973	3.8652705	19.131326	0.0000275	0
10.915127	7.169017	13.418112	0.000018	0
9.533214	10.1478615	-1.5346265	0.00982	0.005256
7.240031	7.677577	-1.354298	0.01005	0.004466

5.7799365	5.277155	1.4270585	0.00061	0.000141
6.945382	7.526906333	-1.497582	0.001018667	0.000234333
7.081856	8.069838	-1.983408	0.000577	0
5.168947	6.1503875	-1.9889765	0.000143	0
8.799941429	8.212405143	1.508269571	0.007872143	0.009120571
8.471196	7.949657	1.435486	0.006747	0.004466
6.258246	6.567429	-1.239006	0.001751	0.000282
3.8353705	4.3307365	-1.409715	0.002929	0.0004925
5.6076585	6.630475	-2.0336335	0.000135	0
8.076798667	6.843999667	2.352826333	0.000022	0
4.364201	4.047655	1.245345	0.011001	0.009809
8.817769	8.024842	1.732586	0.000036	0
8.8419395	9.3218895	-1.398649	0.0203905	0.0189655
6.406515	6.762247	-1.279635	0.024445	0.01988
11.41099	10.755043	1.57565	0.000119	0
7.497066667	6.915939	1.510516333	0.014155667	0.020190333
4.1919405	3.542944	1.597929	0.001971	0.000853
6.913091	7.207351	-1.226256	0.01245	0.009809
6.156118	6.807146	-1.589756	0.00451	0.002233
4.397633	5.264096	-1.823189	0.000223	0
7.621837333	5.633843	4.176114667	1.93333E-05	0
6.99166	6.504319	1.401858	0.007302	0.004466
7.831792	7.362164	1.384752	0.008122	0.009809
8.335396	7.379807	1.93937	0.000123	0
6.174103	7.025707	-1.804507	0.000158	0
8.327839	9.063049	-1.664641	0.001129	0
6.545316	5.9444	1.516679	0.002111	0.000703
7.032608	6.594492	1.354834	0.014243	0.01988
6.964019	7.67980625	-1.6715735	0.002988	0.001187
7.130062	7.649315	-1.4539335	0.009882	0.00994
10.068224	9.137018	1.90687	0.000216	0
8.793531	7.303754	2.808457	0.000028	0
10.873071	9.921791	1.933588	0.000298	0
10.0239435	9.2477945	1.725453	0.0017095	0.000853
5.8914435	6.3846085	-1.4169405	0.0094395	0.004466

7.164548	7.882718	-1.645093	0.000086	0
6.9539435	5.2871515	3.3191235	0.0002115	0
6.746787	7.27203	-1.440248	0.005495667	0.002057333
6.451033	5.641349	1.752828	0.00049	0
4.827969	4.251295	1.491407	0.013185	0.01988
10.213494	10.794224	-1.495606	0.002715	0.000703
10.649256	10.234326	1.333234	0.009025	0.009809
3.346034	3.949667	-1.519538	0.000852	0
9.577101	7.484953	4.263824	0.000019	0
6.331405	4.544257	3.451321	0.000028	0
5.09674	5.665710667	-1.500882667	0.002937667	0.001582667
6.036651	6.543802	-1.421241	0.001563	0.000282
5.292226	6.37552	-2.118869	0.00052	0
6.383221	6.726715	-1.268826	0.023788	0.01988
9.935729	10.57817	-1.560968	0.006215	0.001706
6.960302333	4.082935333	7.960233333	0.000109	0
5.334363	4.279133	2.07805	0.000151	0
6.215912667	4.657587	3.307179333	0.000634333	0.000234333
6.696828	7.2820025	-1.5004295	0.0111435	0.00994
7.525338	5.792819	3.323074	0.000019	0
8.016434	7.65417	1.285441	0.008907	0.009809
6.225869	7.705084	-2.885271333	0.000174333	0
10.560519	9.935314	1.542429	0.005296	0.004466
6.270941333	5.579912333	1.620970667	0.003373667	0.001957333
5.2783855	4.861622	1.338325	0.0147185	0.023017
5.930893	6.4354335	-1.4303005	0.003244	0.000994
7.998132	8.525192	-1.44099	0.005244	0.001706
6.809905	7.911539	-2.145976	0.00008	0
8.5416385	7.884256	1.5796755	0.0083215	0.00994
4.001637	3.095425	1.874119	0.010008	0.009809
6.173412	6.828239	-1.599237	0.000876	0.000141
7.055248	8.0428605	-2.0048815	0.0035925	0.002628
5.124323	5.802142	-1.620396	0.005603	0.002233
3.162292	2.85048	1.241266	0.019418	0.036225
5.471263	4.622347	1.8418795	0.008279	0.0102915

6.018182	5.2635156	1.697353	0.0016138	0.0010338
6.541966	5.822553	1.646512	0.001287	0.000282
5.395666	4.861337	1.448268	0.018437	0.036225
5.629131	4.34711	2.701259333	0.003185667	0.003269667
9.396065	7.186573	4.625123	0.000019	0
6.097327	6.593911	-1.41087	0.000309	0
10.4558	8.21437	4.728655	0.000018	0
8.732725167	8.102772	1.553660333	0.005592167	0.0048755
8.313482	6.243653	4.19837	0.000019	0
7.603558	6.933814	1.590792	0.002007	0.000703
8.5140825	7.016687	2.824602	0.000054	0
6.6046305	6.076794	1.4462605	0.008115	0.0102915
6.793584	7.243776	-1.366221	0.001127	0
9.031565	9.579522	-1.462013	0.000254	0
7.265664	7.768508	-1.417003	0.007543	0.004466
7.412428	6.290712667	2.197557333	0.000047	0
6.6933745	6.2473745	1.369225	0.0123115	0.0181125
5.985429667	5.019776333	2.009182333	0.000787667	0.000234333
7.253407	7.755967	-1.416725	0.010553	0.004466
4.922439	5.336531	-1.33246	0.027742	0.036225
6.9382135	6.0186215	1.904121	0.000172	0
7.379408	7.75422	-1.296671	0.009341	0.004466
8.875383	8.260765	1.531153	0.001256	0.000282
8.563152	9.009047	-1.362159	0.000142	0
4.784647	4.219886	1.479143	0.000976	0.000282
8.0865065	7.3430155	1.705829	0.008768	0.00994
4.299921	3.422834	1.836663	0.002646	0.000703
7.534912	7.243939	1.223465	0.023467	0.036225
9.086798	7.883962	2.301916	0.000061	0
7.303954	6.738523	1.47983	0.001735	0.000703
7.0808	6.344893	1.665444	0.000121	0
11.036926	9.994628	2.059505	0.000171	0
8.172699	9.2606305	-2.1518435	0.000159	0
8.309852	7.690214	1.536489	0.000496	0
4.221139	4.506996	-1.219134	0.002971	0.000703

5.969082	6.47673	-1.42173	0.004358	0.001706
6.758879	5.850385	1.877085	0.000251	0
6.453441	7.130758	-1.599163	0.000565	0
6.7415555	7.578271	-1.78658	0.0003205	0
7.417222667	8.239213667	-1.815725667	0.007875667	0.006720667
7.992531	6.64412	2.571673	0.000052	0
5.392854	5.840693	-1.363995	0.011554	0.009809
8.047457	8.733256	-1.608593	0.000182	0
7.79816	8.37651775	-1.4985655	0.0130355	0.00994
5.465502	5.802365	-1.263007	0.027928	0.036225
8.162368	8.771206	-1.52503	0.008455	0.004466
8.333394	7.936419	1.316744	0.003913	0.001706
7.542135	8.402847	-1.815935	0.002588	0.000703
9.582946	9.004539	1.493199	0.002083	0.000703
7.7817255	9.105402	-2.547243	0.000184	0
5.657845	5.185481	1.387382	0.025784	0.036225
7.022471333	8.325490333	-2.580137333	0.000237667	0
5.614398	5.945805	-1.25824	0.026446	0.01988
5.841885	6.552195	-1.636155	0.031018	0.036225
5.9896765	6.7882105	-1.7394755	0.001462	0.0003515
7.100558	7.782069	-1.603819	0.000209	0
5.1869765	6.108431	-1.935499	0.0007255	0.000141
9.731896	10.189784	-1.37353	0.001085	0
5.577032	6.340113	-1.7321465	0.0052655	0.002233
3.375618	2.931154	1.360808	0.004883	0.004466
5.2852835	5.827636	-1.4671455	0.0072865	0.0050455
5.815076	6.396338	-1.496157	0.000423	0
4.655533	4.3741955	1.2154195	0.01186	0.0148445
7.657473	7.1654465	1.4087245	0.0026995	0.002233
9.6397155	10.400106	-1.7007575	0.001879	0.0003515
10.097064	9.655764	1.357827	0.00342	0.001706
3.949376	3.095867	1.806891	0.00129	0.000282
8.9226935	9.538107	-1.535386	0.0023995	0.000853
7.345878333	6.316023667	2.083910667	0.002160333	0.001582667
10.060017	7.962448	4.279877	0.000018	0

7.575556	6.776974	1.73939	0.000039	0
8.500457	7.250941	2.377616	0.000067	0
7.026269	7.620817	-1.51	0.000187	0
9.2037384	8.0609848	2.2342492	0.0004908	0.0001128
10.86933333	9.557981333	2.513118333	0.000032	0
7.372666	6.251487	2.175464	0.0000255	0
7.404534	6.78796975	1.5515165	0.00435625	0.0060865
7.2276395	8.097064	-1.844028	0.0007485	0.000141
8.229996	7.625567	1.520377	0.001446	0.000282
6.7443605	5.996504	1.698762	0.001999	0.000853
7.047655	6.04792675	2.05231075	0.00141175	0.0011165
5.984691	5.545573	1.355775	0.010162	0.009809
7.859694	8.894131	-2.048313	0.000816	0
4.938512	4.102496	1.785113	0.020489	0.036225
10.174346	10.474985	-1.23169	0.020487	0.01988
5.66155425	6.17107575	-1.4252405	0.0087605	0.005256
6.500079	7.093292	-1.5086175	0.0062285	0.0025845
6.64626125	7.37679725	-1.6719925	0.00142425	0.00024625
7.39384425	8.04972625	-1.583588	0.00423525	0.001543
7.289138	8.3357855	-2.119703	0.000645	0.000141
8.126768	8.648129	-1.435309	0.004837	0.001706
6.39282	7.369347	-1.967722	0.000183	0
6.509987	6.827095	-1.24583	0.0224	0.01988
7.937615	8.376415	-1.355476	0.002674	0.000703
9.938703	9.186866	1.716138167	0.00116	0.0004015
4.015602	4.903502	-1.85048	0.00053	0
7.597968	8.240203	-1.560746	0.000021	0
8.57377	6.148967	5.369556	0.000055	0
6.271844	5.905528	1.289057	0.01331	0.01988
8.170472	6.079462	4.260462	0.000022	0
6.555918	7.428434	-1.830854	0.000249	0
4.0219295	4.5585135	-1.46837	0.0151105	0.0181125
7.507463	6.559672	1.928917	0.003617	0.001706
8.390217	9.366845	-1.967861	0.000555	0
7.335302	6.623938	1.6387065	0.00310575	0.0016135

9.0377095	8.38107	1.5815985	0.019051	0.023017
7.11795	7.434586	-1.245423	0.005122	0.001706
5.801805	6.482394667	-1.607732	0.006332667	0.006626667
9.983303	9.498904	1.399002	0.020249	0.036225
8.509538	8.910673	-1.320546	0.02318	0.01988
7.150916	6.566658	1.499267	0.002716	0.000703
6.5744325	5.4245415	2.2555885	0.000041	0
7.3782435	7.997152	-1.5386635	0.0080435	0.0049045
5.707218	6.394116	-1.609819	0.011072	0.004466
11.267776	11.997527	-1.658353	0.001037	0
7.3672605	8.2815855	-1.8871815	0.000539	0
7.397581	6.186786	2.316337	0.000038	0
8.803767	8.304343	1.413649	0.022318	0.036225
9.732381	9.065351333	1.592855667	0.001093667	0.000234333
7.262249	8.176117	-1.884089	0.000767	0
12.758059	10.260637	5.646756	0.000018	0
11.545545	10.688574	1.811231	0.000162	0
13.799033	12.444232	2.557619	0.000018	0
7.401971667	5.984692667	2.707733	0.000334667	0
5.761711	5.1057855	1.612714	0.0064105	0.0057575
4.815043	4.5080635	1.2372725	0.009596	0.012173
10.619871	11.35472	-1.664223	0.001157	0
6.6042175	7.01287	-1.3299755	0.0188235	0.0148445
8.612597	9.078309	-1.380999	0.004363	0.001706
7.1974715	6.4129245	1.746433	0.0004755	0
6.3399435	5.8767675	1.3808035	0.011188	0.012173
4.051113	4.777457	-1.65444	0.001572	0.000282
6.974993	7.614655	-1.56732025	0.005918	0.0050405
4.8658225	5.516734	-1.572644	0.005139	0.002233
9.7092565	8.094329	3.08541475	0.000028	0
4.818694	4.137274	1.603717	0.013402	0.01988
6.461566	5.766241	1.61925	0.000047	0
6.851766	6.3611245	1.405984	0.001121	0.0003515
9.2908506	10.038551	-1.693302	0.0001934	0
5.0469365	5.4156685	-1.2912305	0.026623	0.023017

5.4078705	5.854511	-1.363983	0.0061345	0.0025845
7.780083	8.452429	-1.593662	0.021205	0.01988
5.452132333	4.771257667	1.655942	0.003672	0.002057333
3.679016	3.327233	1.276137	0.006059	0.004466
9.434818333	8.275128667	2.307546333	0.000150667	0
8.860545	7.290541	2.969054	0.000019	0
7.053922	5.616561	2.708249	0.000019	0
6.567718333	5.214303333	2.677234333	0.000119	0
7.1048185	7.9231125	-1.763434	0.0011725	0.000141
7.2880304	6.1991822	2.2336856	0.0011602	0.0008932
8.170674333	6.863433333	2.588564	5.66667E-05	0
7.410576	6.2018695	2.471547	0.002949	0.002233
5.986914	5.339584	1.566267	0.000499	0
9.290007	7.315237	3.930656	0.000019	0
5.998088	6.399665	-1.320951	0.008649	0.004466
5.372117	6.000076	-1.545377	0.002707	0.000703
5.839295	5.34141	1.412142	0.004791	0.004466
6.727421	5.037937	3.225413	0.000019	0
9.677912667	7.635476	4.152354667	1.83333E-05	0
5.101553	5.6379415	-1.4559495	0.0007045	0
4.745668	4.366021	1.301024	0.011944	0.01988
7.52309	6.416514	2.15334	0.000028	0
6.964443	6.007895	1.940662	0.000536	0
4.217204	3.827327	1.310282	0.002707	0.000703
5.881233	5.546387	1.261244	0.021586	0.036225
7.6543865	8.586138	-1.941867	0.0024755	0.000853
5.696689667	6.377593	-1.64662	0.012286	0.013563667
5.887464	6.452109	-1.479024	0.013597	0.009809
7.36819475	6.59646425	1.71569775	0.00026375	0
3.583225	3.205112	1.299641	0.012662	0.01988
5.930425	6.321659	-1.311515	0.006177	0.001706
7.592048	7.980632	-1.309108	0.016485	0.009809
7.713241	6.462033	2.380407	0.000032	0
7.156574	5.737206	2.674683	0.001514	0.000282
5.118094	5.639006	-1.434863	0.020483	0.01988

4.921695667	5.471807667	-1.476764333	0.010115333	0.006539333
6.496608	6.885799	-1.3111045	0.02174	0.0203455
7.966678	8.325242	-1.282149	0.025503	0.01988
6.6666675	5.9038175	1.7024635	0.001156	0.0003515
11.186309	10.44076975	1.6777775	0.000512	0.0000705
6.9929575	5.346163	3.5350565	0.0000625	0
9.527336	9.852212	-1.252557	0.036222	0.036225
5.984879	6.525606	-1.454705	0.018182	0.01988
7.075584	7.81219	-1.666251	0.000271	0
9.604005	10.120472	-1.430448	0.005875	0.001706
6.9730698	5.7292588	2.4552894	0.0002724	0.0000564
9.936037	8.713991	2.332772	0.000023	0
8.4893765	7.479243	2.041782	0.000027	0
8.437397	9.016866	-1.494299	0.001076	0
7.652902	8.0436375	-1.3123145	0.023965	0.0203455
6.26758875	7.17644375	-1.882283	0.000968	0.0000705
8.7025155	9.287909	-1.501859	0.0150585	0.0182535
4.65144475	5.23793225	-1.515497	0.00114825	0.00017575
6.122658	6.768791	-1.564968	0.001918	0.000282
6.426169	5.631501667	1.781269333	0.005185667	0.006626667
4.536011	4.946063	-1.328734	0.006857	0.001706
10.626219	10.986808	-1.28395	0.027684	0.036225
6.029199	5.525132	1.418207	0.002741	0.000703
8.57976	9.730898	-2.22089	0.000026	0
5.810287	4.242923	2.963626	0.000033	0
7.878169667	6.690345333	2.470483667	0.000232667	0
8.867406	9.218801	-1.275794	0.032943	0.036225
4.811650333	4.232269	1.49842	0.006779667	0.006539333
7.383985	5.893525	2.809786	0.000023	0
8.057126	8.478212	-1.338936	0.001646	0.000282
6.008401	5.025835	1.975976	0.000059	0
4.854814333	4.147431	1.648147667	0.005582	0.006955
5.95618	6.852171	-1.860889	0.001354	0.000282
7.869614	8.464806	-1.510891	0.0005355	0
7.280098667	7.999565333	-1.650589333	0.003433667	0.000803

5.274118	5.701769	-1.345041	0.003147	0.000703
6.020525	6.731585	-1.637006	0.000294	0
8.402470667	9.265622333	-1.828802	0.001094333	0.000234333
6.719605	7.227954	-1.422421	0.0245	0.01988
7.448168	6.022042	2.68724	0.000049	0
5.263842	4.908794	1.279029	0.008983	0.009809
6.839487	7.111511	-1.207501	0.010925	0.004466
7.719954	5.694464	4.071301	0.000101	0
6.201801	5.688672	1.427142	0.003649	0.001706
5.680028	5.960493	-1.214586	0.0245	0.01988
6.96721	4.770399	4.584647	0.000029	0
6.160304	6.611799	-1.367456	0.022299	0.01988
6.246312	7.287496	-2.057916	0.00008	0
8.545992	7.992563	1.467569	0.013409	0.01988
5.446735	7.2091915	-3.4011795	0.0000285	0
5.479432	5.98214	-1.416871	0.001455	0.000282
5.874262	6.171441	-1.228739	0.024496	0.01988
6.152493	6.449888	-1.228923	0.01647	0.009809
5.893751	6.391692	-1.412197	0.004911	0.001706
8.945935667	7.431037667	2.863804333	0.000024	0
5.7528435	5.1363435	1.5358005	0.001198	0.0003515
10.815822	9.223707	3.014911	0.00002	0
9.079175	6.667899	5.319447	0.000018	0
8.2952435	8.757811	-1.378035	0.03684	0.036225
5.797619	4.813638333	2.01007	0.001034333	0.000234333
9.1050945	8.3070565	1.7890715	0.009084	0.00994
7.500614	7.083872	1.33491	0.001647	0.000282
6.761614	6.188113	1.48813	0.001004	0.000282
4.940678	4.670335	1.206095	0.010515	0.009809
5.611434	4.9973515	1.5668765	0.001565	0.0003515
7.8813965	7.0138385	1.836079	0.0019515	0.000853
7.5294026	6.8236736	1.6482108	0.0031808	0.0022994
5.561887	5.266929	1.226849	0.011759	0.009809
5.496316333	4.944833333	1.472877	0.001696333	0.000662667
6.08314	6.841964	-1.69211	0.000182	0

7.310462667	6.798741667	1.434883333	0.003898	0.003363667
5.9561185	5.5173345	1.356033	0.008674	0.009809
7.965951	8.7730935	-1.76753	0.0032775	0.000853
8.481494	9.321683	-1.790284	0.000085	0
6.8673875	6.373793	1.408257	0.000661	0
5.786592	6.28818	-1.415772	0.033146	0.036225
5.144646	5.47372	-1.256207	0.008645	0.004466
6.2548695	5.531735	1.654309	0.000189	0
6.561341	5.479758	2.116357	0.000033	0
5.8676735	5.2615795	1.529752	0.01393	0.018464
4.967459	5.63581	-1.589256	0.000681	0
6.377178	5.203822	2.255357	0.000809	0
7.517039333	6.805945333	1.643429	0.00078	0.000094
7.880945	8.383424	-1.417656	0.0138615	0.010793
6.948874	6.44184	1.421126	0.000892	0
6.722988	7.086011	-1.286117	0.009652	0.004466
6.21541	6.7570515	-1.4556505	0.003462	0.000703
6.868435667	6.02248	1.806983	0.000606	0
6.543911	6.143182	1.320175	0.007768	0.009809
6.2002815	5.48499075	1.653036	0.00188475	0.0011165
7.549048	8.851335	-2.466194	0.000084	0
7.388513	6.923705	1.380134	0.008091	0.009809
11.106158	9.658433	2.727775	0.00002	0
8.286037	8.960788	-1.596321	0.000951	0
7.550556	8.016554	-1.381272	0.002406	0.000703
3.883022	4.328818	-1.362066	0.022851	0.01988
9.965787	10.453783	-1.402495	0.003777	0.000703
5.235095	4.699369	1.4507845	0.0168635	0.0280525
7.835249	6.5041985	2.531695	0.000031	0
4.927217	5.573226	-1.564834	0.004893	0.001706
6.198345	6.600181	-1.321188	0.006006	0.001706
8.110126	7.370112	1.672408	0.000043	0
8.00917425	7.45706	1.47374825	0.00431225	0.0053965
7.8292388	6.911151	1.9068488	0.0009034	0.0002534
8.068206333	7.380277833	1.632063167	0.0076895	0.008545833

8.764613667	7.572647	2.369341667	0.002550667	0.001488667
6.950187	7.431945	-1.396445	0.00159	0.000282
8.712941	7.034644	3.2005	0.000019	0
8.0277815	7.1726725	1.83815	0.0000875	0
5.943675	6.771909	-1.775511	0.000117	0
4.622043	5.1867505	-1.5104155	0.0176155	0.0182535
7.20188	7.841463	-1.557879	0.004239	0.001706
6.928975	7.233341	-1.234876	0.014039	0.009809
8.7329385	8.219461	1.4278175	0.003713	0.002374
7.451604	7.058854	1.312894	0.002306	0.000703
8.9318415	7.9067205	2.0755375	0.001085	0.0003515
8.924477	6.195869	6.7934935	0.0000185	0
8.277332667	7.649910333	1.569442667	0.003855	0.002151333
6.288776	5.220529	2.096884	0.000946	0
6.254809	5.495786	1.692345	0.002009	0.000703
7.661119	8.212962	-1.465958	0.003053	0.000703
4.923933	5.467291	-1.457361	0.000461	0
4.562584	4.88155	-1.247436	0.018545	0.01988
8.888425	9.28405	-1.315512	0.017979	0.01988
7.130867	7.712161	-1.496191	0.003522	0.000703
9.804018	10.397675	-1.509067	0.00082	0
7.2474605	5.9970265	2.3828065	0.000059	0
8.071828	6.998792	2.161926	0.000529333	0.000047
9.33949	9.670576	-1.25796	0.019482	0.01988
7.897752	7.015971	1.842648	0.000086	0
5.46802	4.585071	1.844141	0.009297	0.009809
5.2334195	5.9084825	-1.6100665	0.0016925	0.0003515
9.165397	9.823695	-1.578219	0.000151	0
6.3061025	5.669868	1.554305	0.009072	0.009809
7.5556415	8.040963	-1.4000885	0.0093925	0.0057575
5.991825	4.922541571	2.228536714	0.000916714	0.000243714
6.370094	6.652292	-1.216046	0.025813	0.01988
7.416103	8.567458	-2.221225	0.000046	0
8.54127	7.786651	1.687185	0.000051	0
7.54783	8.612778	-2.092095	0.000094	0

6.4947835	5.3088525	2.402013	0.002153	0.000853
6.896005333	6.313467333	1.512383333	0.003579	0.003269667
6.568474	7.451388	-1.844096	0.000062	0
6.036731	5.2373735	1.7461535	0.000774	0.000141
5.027444	4.437698	1.504981	0.008574	0.009809
10.042293	11.025607	-1.977001	0.000126	0
5.9355115	6.2948855	-1.283682	0.002876	0.000994
5.516257	6.343537	-1.774338	0.000299	0
6.212757	6.700508	-1.402258	0.000561	0
5.208505	5.764899333	-1.470913333	0.011766667	0.009990333
5.611845	5.992673	-1.302089	0.002326	0.000703
4.4634955	5.1687715	-1.6358015	0.0015865	0.0003515
4.6295735	5.315531	-1.613661	0.00698	0.0049045
6.735678	7.162932	-1.344672	0.002363	0.000703
5.919272	6.443351	-1.438015	0.003739	0.000703
7.058836	7.557701	-1.413101	0.02446	0.01988
6.329533	6.85385	-1.438253	0.000944	0
6.559179	6.158145	1.320455	0.008283	0.009809
4.13805	4.93237	-1.73426	0.000184	0
6.762064	7.323327	-1.47556	0.022563	0.01988
4.804833	4.008611	1.736548	0.001695	0.000703
3.746615	4.14118	-1.314547	0.010812	0.004466
6.098196	6.503423	-1.329612	0.001046	0.000141
6.676222	5.448822	2.3430155	0.00003	0
5.83534	6.275819	-1.357054	0.008314	0.004466
5.824208	6.771783	-1.928629	0.000624	0
5.710501	6.304901	-1.509845	0.000545	0
5.644864	6.116235	-1.3964765	0.0036705	0.002233
7.211256667	7.672596333	-1.380000667	0.018128	0.011385
4.787503	5.767885	-1.972988	0.000301	0
4.743432	5.041891	-1.2298315	0.0103415	0.010081
5.858112	6.678356	-1.765704	0.000169	0
5.912277	5.5523155	1.283538	0.0160385	0.01988
7.611817667	8.044093	-1.349581333	0.009389333	0.007429667
5.707155	6.285303	-1.492931	0.002096	0.000282

6.133352	6.625411	-1.406451	0.011344	0.004466
6.206056	6.850065	-1.562665	0.000028	0
5.31359	6.5193925	-2.3744055	0.0001575	0
6.47418	7.191765	-1.644427	0.000482	0
5.408523	5.85773	-1.365289	0.009472	0.004466

Mean Signal of Group Sepsis(GSE28750)	Mean Signal of Group Control(GSE28750)	Fold Change(GSE28750)	p-value(GSE28750)	FDR(GSE28750)
6.58833	7.058423	-1.385199	0.012036	0.003006
7.947649	7.411685	1.44991	0.007441	0.003006
8.4821765	8.00172	1.3990945	0.002049	0.0003575
6.786925333	5.0320535	3.834150333	0.001117167	0.000272333
7.495813	6.3378655	2.231401	0.000503	0
3.607251	4.110076	-1.416985	0.008487	0.001402
7.8004255	7.1739305	1.554169	0.004967	0.0017445
7.845338	7.308845	1.450442	0.004032	0.001402
4.4868975	5.7057085	-2.3367675	0.0003775	0
6.40559	4.352575	5.2443305	0.0002605	0
4.916333	4.327145	1.5044	0.00079	0
9.033953	7.9996395	2.048183	0.007515	0.007897
4.662604	5.2356935	-1.5133185	0.0086335	0.0035955
9.544091	8.143615	2.639886	0.000586	0
5.526499	4.260025	2.405728	0.002181	0.000232
5.919855	6.652924	-1.6649815	0.003091	0.000701
8.6293405	6.85446475	3.47839875	0.000133	0
7.374003	7.026563	1.2741395	0.011351	0.008482
8.694045333	7.963704333	1.660093667	0.012843667	0.015813667
9.394103	7.467431	3.801772	0.000554	0
8.642091	7.734125	1.876399	0.004612	0.001402
7.732205	7.318465	1.332134	0.00879	0.007191
8.856557	8.238908	1.534374	0.003421	0.000483
6.30586	7.045039	-1.669226	0.00302	0.000232
9.411892	7.703796	3.267292	0.000618	0
7.658268	8.318696	-1.580552	0.0039	0.000232
4.129118	4.471342	-1.267709	0.015854	0.007191
4.731024	4.220831	1.424241	0.022806	0.031647
5.6492465	6.281516	-1.5510015	0.009495	0.0035955
8.885294	9.646716	-1.695161	0.003716	0.000232
7.510286	6.905883	1.52035	0.000597	0
7.21854	7.701086	-1.397207	0.001388	0
8.078458	8.615813	-1.451309	0.020121	0.007191

7.264498	6.090069	2.257036	0.000019	0
9.7841375	8.1389555	3.206094	0.000058	0
11.448166	7.440782	16.082103	0.000018	0
5.991652	6.396536	-1.323982	0.004843	0.000483
7.20097	6.48713	1.6427475	0.0021895	0.000701
8.9301732	8.451218	1.3989436	0.0170892	0.0210276
6.299138	7.595827	-2.456645	0.000243	0
5.989148	4.004499	10.32821567	0.007216667	0.004794
5.284681667	4.740061333	1.464455667	0.010664	0.010535667
5.860637	6.389589667	-1.458143667	0.014632333	0.007661667
6.237289	6.7416295	-1.418939	0.004527	0.000483
7.94529	6.781738	2.240083	0.000202	0
8.5720105	7.784503	1.726492	0.003473	0.0009425
10.782799	10.295842	1.401486	0.007567	0.003006
6.289773	5.833556667	1.373987	0.009963	0.007191
10.17345633	9.523551	1.574023667	0.011544	0.009981333
5.416908222	3.862915667	3.543403222	0.002821111	0.003567889
6.2529938	6.8388302	-1.5069464	0.002856	0.0003268
7.870107	7.372261	1.412103	0.004219	0.001402
7.4224225	5.593108667	3.640643667	0.000120333	0
7.022462	5.830442	2.326462	0.000309	0
6.352298	7.343418667	-2.010136	0.016317333	0.010374667
5.121716333	5.769449667	-1.576310667	0.006030333	0.001079333
9.227126	8.727	1.4224232	0.0149348	0.0145936
8.180039667	6.358462	3.698161	0.000713	7.73333E-05
5.537605	3.82649	3.274139	0.000875	0
6.7582795	5.3754425	3.077332	0.001711	0.0002415
7.587521	8.090025	-1.41667	0.01078	0.003006
6.535471	5.241776667	2.483091167	0.000536333	3.86667E-05
7.745063333	8.613257	-1.829851333	0.000335333	0
7.984137	7.238697	1.676485	0.000048	0
5.782263667	4.242046667	3.222111	0.005218333	0.005187333
10.927371	9.508977667	2.68582	0.005793333	0.005187333
6.1219545	6.6740955	-1.467091	0.014341	0.0050985
4.15792875	4.8210645	-1.595637	0.010668	0.004299

6.808638	5.961382	1.799077	0.000956	0
10.406776	9.046234	2.567815	0.00002	0
7.130236	5.475224	4.678203	0.008259	0.007781
5.639081	6.151074	-1.426019	0.044463	0.031647
9.15556	8.644517	1.4277565	0.0044205	0.0017445
4.0104965	5.009082	-2.0797635	0.002765	0.0002415
4.9178305	5.3642465	-1.372394	0.0023895	0.000116
7.295311	8.44779	-2.2806715	0.0008595	0
6.554052	7.895522	-2.568731	0.010669	0.002204
7.37334475	8.1102925	-1.66902475	0.011587	0.00802775
7.371281	8.298834	-1.902047	0.000556	0
6.1853675	5.6451815	1.460338	0.004595	0.0017445
10.453107	11.025647	-1.48714	0.005154	0.000483
6.7566445	7.362271	-1.530586	0.003216	0.0002415
6.883506333	7.534105667	-1.591891667	0.002596333	0.000467333
10.782194	9.860506	1.894331	0.000184	0
5.332263	5.9034775	-1.48764	0.0227135	0.0159395
6.674282	5.205192	2.768472	0.002485	0.000483
7.393289667	7.806935	-1.333859	0.022573	0.015343
9.669542	7.926534	3.347323	0.000029	0
6.703016	5.056353	3.131085	0.000959	0
5.8788625	4.59296	2.6041865	0.0002725	0
8.9568915	6.711671	4.9288155	0.0000405	0
10.617309	9.522276	2.136179	0.003314	0.000483
6.67314625	8.0360425	-2.58281375	0.00184575	0.000058
9.1466715	8.3213815	1.7722265	0.0022825	0.000701
9.498317	8.119017	2.601421	0.000022	0
6.211390333	5.348137333	1.823944333	0.003760667	0.001240333
9.719875	8.011569	3.26777	0.000025	0
4.549316	5.999423	-3.23068	0.000925	0
8.9366965	8.4249135	1.426226	0.0128025	0.009284
4.518655	5.424233	-1.873295	0.001704	0
5.088621	4.487741	1.518613	0.003023	0.000701
7.075183	8.354345	-2.42698	0.000021	0
7.2425915	6.227984	2.116487	0.011564	0.0159395

6.906507	7.523915	-1.534116	0.000069	0
5.3394535	6.4906815	-2.2589875	0.0004515	0
6.589623	7.776539	-2.276655	0.000247	0
10.979723	10.56231	1.33553	0.02759	0.031647
6.8750155	7.568246	-1.619131	0.00087	0
4.77323	7.494865	-6.5962	0.000152	0
7.607354333	5.509530667	4.294978	0.000153333	0
11.224298	5.49747	52.959871	0.000018	0
7.143936	8.735003	-3.01272	0.000037	0
8.428824	9.119957	-1.614551	0.00591	0.000483
11.21645567	9.515696667	3.2555	0.000688	7.73333E-05
12.219826	10.713407	2.841038	0.000024	0
7.943771	7.316808	1.5446175	0.009835	0.007191
4.296984	5.0740365	-1.727142	0.004137	0.0003575
2.810608	2.467464	1.268518	0.008582	0.007191
9.5354415	8.4969625	2.085363	0.005626	0.00796975
9.859105	10.543761	-1.607318	0.003783	0.000232
5.836696	6.410166	-1.4954145	0.0007995	0
8.130778	6.1353905	4.0058695	0.0000315	0
8.36973	7.721514	1.567229	0.000654	0
6.32277	7.113032	-1.729388	0.001404	0
6.479161	7.581847	-2.147541	0.000026	0
8.119753667	6.586340333	2.920908333	0.000647333	0
5.959941	6.269988	-1.239753	0.0222535	0.009284
5.488682	6.681553	-2.286072	0.000042	0
10.341846	8.135157	4.632359	0.000022	0
9.5798545	5.72242	14.6422575	0.000018	0
12.0975	11.334999	1.696429	0.000111	0
4.739046	5.8070085	-2.1381345	0.000278	0
7.955962	8.7987835	-1.8058515	0.001541	0
7.482531667	8.176242	-1.617629667	0.004578333	0.001002
3.407712	3.828961	-1.339087	0.046336	0.031647
7.3152845	6.701594	1.533214	0.0036355	0.001503
6.456773	6.926709	-1.385048	0.001145	0
5.9686375	4.9202445	2.108087	0.0018005	0.0002415

7.923015167	6.278919833	3.3782145	0.0021985	0.002593667
8.370281	8.836041	-1.381045	0.005288	0.000483
7.4157785	7.93217	-1.4423465	0.021362	0.0173265
6.3450082	7.0515506	-1.6811878	0.0064004	0.0018616
7.335769	7.937701	-1.517748	0.022608	0.015562
7.593799	6.4010625	2.365516	0.0031205	0.000483
8.603074	6.890151667	3.635283333	0.00091	7.73333E-05
8.2515345	8.90447	-1.5729385	0.0016375	0
12.666703	10.432467	4.705133	0.000018	0
4.95943	5.6601	-1.625259	0.006684	0.001402
9.927915667	9.459177	1.387064333	0.018702667	0.018133333
4.995659	5.416809	-1.338994	0.011641	0.003006
7.818007	6.744916	2.103936	0.000038	0
5.8801605	6.5208855	-1.5654335	0.012869	0.0080225
7.18857	7.5741935	-1.306453	0.006449	0.001503
5.6251975	6.31495725	-1.68104475	0.00352575	0.00047125
7.618128	8.220231667	-1.51867	0.004801333	0.000628333
8.53390575	5.958925	7.452266	0.00441325	0.0038905
7.360156	8.067069	-1.632308	0.017038	0.007191
12.261113	9.477844	6.884105	0.000018	0
4.2172935	3.283572	1.9112305	0.0009265	0
6.79735	5.179417	3.069349	0.001799	0.000232
6.916424667	4.130993	7.063528	2.83333E-05	0
4.184812	4.561818	-1.2986935	0.018946	0.009284
4.613468	5.8633155	-2.37825	0.0012555	0
6.856653	7.292425	-1.352634	0.004004	0.000232
6.4940565	6.201252	1.2251255	0.016633	0.0236045
8.552242	6.853977	3.252642	0.000049	0
6.770843	5.397612	2.590501	0.000067	0
9.739922	10.165253	-1.34289	0.014701	0.0050985
6.31482825	7.175777	-1.84553825	0.00394775	0.0007515
6.801143	7.967374	-2.284806	0.0000955	0
7.37495	7.835479333	-1.378409667	0.002273333	0.000161
6.119604	6.795651	-1.597756	0.00554	0.000483
5.284161	5.801656	-1.431468	0.011535	0.003006

8.469216	7.815639	1.573064	0.001478	0
6.842809	5.7161805	2.200477	0.0011715	0.000116
5.991438333	6.597958333	-1.535495	0.002516667	0.000467333
6.646043	5.495667	2.235053	0.000186	0
6.468594	7.241726333	-1.726995	0.001265667	7.73333E-05
4.647848	3.828528333	1.772408	0.004709333	0.002558
3.904515	4.400598	-1.410379	0.033485	0.031647
5.8814055	5.111674	1.734379	0.0019775	0.000232
7.623596	7.21863	1.324058	0.012981	0.015562
7.668771667	7.083359	1.505370667	0.00452	0.002864333
8.634065	7.925508	1.634169	0.001134	0
7.313331	8.026528	-1.639434	0.000115	0
6.030553	6.407073	-1.298831	0.008566	0.0017445
9.3493945	8.6710605	1.602724	0.004392	0.001619
8.372017	6.304532	4.191556	0.000155	0
6.847347	5.575445	2.420037333	0.003694667	0.001163
8.486431	6.982116	2.8369	0.000128	0
5.86354225	7.3266135	-2.890617	0.00029875	0
10.748395	9.311407	2.707549	0.000034	0
7.661792	6.453132	2.311229	0.00067	0
7.734666	8.073051	-1.26434	0.033974	0.031647
7.156084	7.6593505	-1.4174895	0.0030555	0.000232
6.876758	6.352531	1.447554	0.0031555	0.000817
6.7163195	7.2894875	-1.4878265	0.0064385	0.001503
9.904761	10.767172	-1.818074	0.007099	0.001402
7.777581	6.541857	2.393779667	0.001567333	0.000238333
5.02472025	5.66384675	-1.560527	0.00491525	0.00052925
6.428623	7.082580667	-1.5763	0.003721	0.000544667
7.6176135	6.3542155	2.4884265	0.0000945	0
10.079724	9.632737	1.363191	0.013062	0.015562
5.341626667	6.052656	-1.664756	0.002397	0.000161
6.785831	5.324583	2.753465	0.001047	0
6.28449	7.168819333	-1.893193667	0.001855	7.73333E-05
6.5483505	5.876163	1.603011	0.012155	0.0113765
5.080241	4.700581	1.301035	0.022055	0.031647

6.731326	7.955819	-2.336733	0.000038	0
6.244134333	5.352679333	1.917272	0.008658	0.010626333
7.363811	5.9302525	2.7247185	0.0009015	0.000116
8.012332667	6.512603667	2.884696	0.000206667	0
5.395577333	4.700651667	1.636957	0.008965	0.010374667
7.970974333	6.233506667	3.404431667	8.96667E-05	0
5.333724	5.069368	1.2011	0.01624	0.015562
6.885688333	5.725136667	2.310202333	0.000483667	0
4.9484955	3.9680595	1.999436	0.000953	0
4.7691835	5.941669	-2.286393	0.001447	0
5.367526	6.062963	-1.619375	0.010904	0.003006
4.325288	4.028569	1.228347	0.007758	0.003006
7.937853	7.17945	1.691618	0.000773	0
7.205171	6.4525755	1.6871955	0.007176	0.0042965
7.9540635	6.886927	2.157284	0.00076	0
5.625767	6.818669	-2.286121	0.000113	0
5.99241	5.295469	1.621065	0.003165	0.000483
3.08092	3.434633	-1.277845	0.017922	0.007191
7.871718	6.136474	3.329358	0.000075	0
4.610653	5.310111	-1.623895	0.000257	0
4.257892	5.016996	-1.692438	0.000376	0
5.512017	5.851728	-1.265503	0.004985	0.000483
8.3501325	7.802389	1.468231	0.0156615	0.019419
7.566258125	6.620271125	1.950573875	0.000837875	0.000058
5.9160865	5.0983925	1.7989595	0.0044035	0.00267
6.9714938	5.384205	3.1529596	0.0007152	0.0000464
5.009378	8.433791	-10.736211	0.000223	0
12.480463	10.940728	2.9111145	0.0000395	0
5.244872	5.604409	-1.283014	0.024855	0.015562
7.3776545	9.94118	-5.9126685	0.000019	0
7.304255	5.836263	2.766367	0.000407	0
9.261753	8.590536	1.592416	0.002018	0.000232
6.531374	8.772833	-4.72875	0.001543	0
7.0854748	6.1889898	1.9151804	0.0118268	0.0109766
9.569088	10.581489	-2.017266	0.004229	0.000483

10.795688	9.882886	1.882698	0.000944	0
4.33148	4.8225545	-1.414174	0.0009885	0
8.900562667	7.147656667	3.472165333	0.001894333	0.000467333
6.165989	5.20245	1.950087	0.000965	0
8.59180875	7.66965775	1.9413135	0.00581425	0.00796975
10.683898	9.960274	1.651325	0.003758	0.001402
5.523891	4.965007	1.47313	0.001954	0.000232
5.8546495	7.4999175	-3.3732415	0.000022	0
4.745138	5.9267505	-2.313615	0.006977	0.0035955
4.51224	5.274623	-1.70326	0.000548667	0
6.087742	6.902559	-1.759076	0.000036	0
9.307082	7.043102	4.803147	0.000368	0
7.2232945	6.1817435	2.1202745	0.001514	0.000116
7.616545333	8.300974667	-1.614515	0.005871667	0.001163
6.918886857	7.797737429	-1.869132429	0.002045714	0.000429429
4.445565	5.73933	-2.45167	0.000115	0
5.681698333	6.314381333	-1.554816	0.007043333	0.002474333
9.351024	6.277619	8.417576	0.000019	0
8.319645	6.612083	3.266084	0.000026	0
12.978202	12.0921922	1.8483634	0.000342	0.0000464
8.3653492	6.9893442	2.6273762	0.0002646	0
6.378722	6.722931	-1.269455	0.010756	0.003006
7.639588	6.591695	2.067508	0.000194	0
7.256956	7.995496	-1.668487	0.004035	0.000232
6.0808675	5.187901	1.8849275	0.0008625	0
5.90993	4.211694	3.24504	0.000115	0
8.759326	10.147702	-2.617837	0.000916	0
4.284572	3.80615	1.39322	0.003774	0.001402
8.752049	8.0832655	1.5976725	0.003496	0.001503
11.756956	11.098093	1.578838	0.0005	0
5.881068	6.892748	-2.016258	0.00009	0
8.21334	11.032723	-7.0605485	0.000093	0
6.729287	7.150376	-1.338938	0.004119	0.000483
4.936184	5.90826	-1.961661	0.000077	0
5.283541	5.839707	-1.470357	0.017608	0.007191

7.0042175	7.4950985	-1.4059615	0.0042375	0.0003575
6.707635	7.541716	-1.784216	0.007938	0.0017445
9.068229	7.080144	3.9671	0.000737	0
6.739821	8.684755	-3.8502	0.00003	0
8.976883	4.99586	15.790918	0.000018	0
7.566549	6.9469645	1.547295	0.01046	0.0159395
3.325572	4.200317	-1.833683	0.003938	0.000232
6.9382095	4.87553875	4.838976	0.000044	0
9.000273	8.057824	1.921788	0.001133	0
6.325738	7.296876	-1.960386	0.000405	0
5.683059	6.8250245	-2.2700565	0.0009755	0
7.321587	8.110759	-1.728088	0.000061	0
**11.5210715**	**8.5480045**	**7.8559415**	**0.0000195**	**0**
7.651207	9.463026	-3.510846	0.004821	0.000483
7.0440695	7.5583635	-1.428918	0.020825	0.0113765
10.191051	9.84157	1.274102	0.018524	0.015562
6.675863	6.224848	1.367002	0.020117	0.031647
5.537071	6.287741	-1.682574	0.00032	0
9.002934	7.985149	2.024808	0.00027	0
5.417197	6.318993	-1.86839	0.009063	0.001402
3.886539	3.505985	1.301842	0.014888	0.015562
5.5092318	3.8442766	4.1338638	0.0039416	0.0031588
8.45995	7.48024	1.972069	0.001998	0.000232
8.126272	7.275961	1.802889	0.013514	0.015562
8.754981	7.3921625	2.590597	0.0020535	0.0002415
6.1558295	5.133924	2.109856	0.0115995	0.009284
5.988709	6.779372	-1.7326	0.0022985	0.0002415
8.389868	7.06505	2.505013	0.000202	0
10.931347	8.492121	5.423507	0.000018	0
5.025167	6.168204	-2.208454	0.000022	0
7.642385	9.441639	-3.480402	0.000055	0
7.0357	8.875064	-3.578522	0.000019	0
5.797545	7.197538	-2.639004	0.000029	0
6.507615	8.0415182	-2.9342598	0.0008032	0
9.3806955	11.330646	-3.8702655	0.0001595	0

10.987502	8.680583	4.948253	0.000126	0
8.793369333	9.378087667	-1.503492	0.010804	0.003476333
6.390416	7.058843333	-1.611722	0.013488333	0.011016333
7.015889	7.7010575	-1.6079115	0.015467	0.007897
5.946239	6.370473	-1.341859	0.032774	0.031647
6.606229	7.533538	-1.91127375	0.001234	0
6.871721333	7.507064667	-1.554600333	0.006125333	0.001012
7.937377	8.718954	-1.7190495	0.0026845	0.0002415
7.403413667	7.785883667	-1.304634333	0.025278667	0.013948
7.276647	8.060505	-1.721729	0.000603	0
10.631847	5.750765	29.8013735	0.000018	0
9.351791	10.001292	-1.57097	0.007564	0.001503
6.78690625	4.33478625	5.93529525	0.00032475	0
9.112773	8.064021	2.06874	0.000647	0
4.550169	4.233911	1.245097	0.001765	0.000232
6.81369	6.018644	1.735133	0.001035	0
5.451811	6.846463	-2.629252	0.000097	0
9.398476333	8.442345333	1.952872667	0.002042333	0.000322
8.95731	9.58109	-1.540907	0.014815	0.007191
7.909665	9.242362	-2.51873	0.000025	0
7.434335	6.265675	2.248028	0.001446	0
8.848511	7.768964	2.113373	0.001072	0
6.174502	8.447924	-4.834685	0.002785	0.000232
10.632453	10.107893	1.438495	0.014687	0.015562
8.138439	7.2680495	1.851668	0.003118	0.000817
6.789081	7.961147	-2.253341	0.000113	0
8.238477	5.302651	7.65194	0.000375	0
11.285713	9.297548	3.967323	0.000043	0
7.905515	5.882168	4.065257	0.000091	0
12.093648	8.692635	10.686919	0.0000405	0
9.323355	8.433938	1.852427	0.002388	0.000483
8.7333145	10.9706295	-4.7187145	0.0000995	0
4.292554	3.478743	1.7710585	0.0043475	0.001503
5.535647	5.9135	-1.299407	0.01735	0.007191
5.664107	4.403534333	2.767342333	0.002466667	0.001002

8.596190333	5.776000333	7.308165667	2.03333E-05	0
8.782917	7.904897	1.8634965	0.0089045	0.0080225
8.847671	10.091467	-2.369048	0.000362	0
5.47627275	6.74207475	-2.42323575	0.0107465	0.0045915
5.7713825	4.250508	2.9176625	0.0013645	0.0002415
10.648746	8.976589	3.186908	0.000026	0
6.11092375	7.0882785	-2.098198	0.00198475	0.0003505
6.410713	5.677393	1.66246	0.00169	0.000232
6.633235	6.052207	1.495915	0.01164	0.007191
5.768732	5.394641	1.296023	0.002121	0.000232
5.663559	4.3633535	2.4710385	0.0010455	0.000116
7.6529745	8.4170055	-1.7030415	0.003349	0.0007515
6.841298667	5.560491333	2.439760333	0.001989	0.000467333
6.586423	7.325957	-1.669636	0.002255	0
5.33844	3.7601405	3.643761	0.0017775	0.0002415
6.798025	6.414074	1.304911	0.007467	0.003006
6.096279	7.469757	-2.590945	0.000078	0
8.8012855	9.353248	-1.4720445	0.0126655	0.007781
5.284741	6.397857	-2.163123	0.000037	0
8.318981	7.465188	1.815353333	0.000970667	7.73333E-05
7.917249	8.537703	-1.537358	0.002137	0
6.723637	7.01463	-1.223482	0.040241	0.031647
5.755987667	4.673232333	2.13637	0.001895	0.000238333
5.911526	5.403165	1.428744333	0.006975667	0.004794
8.073529667	6.100802667	3.937543333	2.43333E-05	0
6.5170675	5.944745	1.4869715	0.001542	0.0002415
4.66107	5.539984	-1.838991	0.002005	0
5.3465965	5.88765825	-1.46406475	0.0044995	0.000701
4.486139	4.05116	1.351966	0.0081435	0.007781
6.7177435	6.0185945	1.63606125	0.00881325	0.00815325
5.0828895	5.86309875	-1.73236325	0.00310825	0.0007515
7.242841	9.277452	-4.097123	0.000235	0
6.365979333	7.302573	-1.931844333	0.008026	0.002635333
6.2599845	5.7853445	1.38960875	0.00503175	0.0038905
5.164657	5.806492	-1.560312	0.003499	0.000232

5.331681	5.904174	-1.487091	0.000816	0
7.9634325	7.1891375	1.724318	0.006267	0.0037115
10.253587	9.6633	1.505546	0.000675	0
10.318041	9.170461	2.2161015	0.0000895	0
7.025603	6.161135	1.820668	0.025309	0.031647
4.71722	5.428645	-1.637421	0.003364	0.000232
8.1710295	7.3413625	1.796512	0.002712	0.000701
12.094117	10.314711	3.432848	0.000029	0
6.2428715	5.214178	2.2050105	0.013182	0.0158235
7.7658545	8.620587	-1.808485	0.003511	0.000701
12.267577	10.921767	2.541729	0.000047	0
7.1201575	6.3573585	1.737707667	0.007312167	0.0070545
4.931448	5.582292667	-1.580481333	0.009480667	0.005187333
7.834215	6.265890667	3.211908	0.001721667	0.000161
6.854747	5.0223045	3.58408325	0.00014375	0
5.591562	6.412991	-1.767155	0.003274	0.000232
7.954866	6.630378	2.504441	0.000277	0
8.371394	7.308309	2.089394	0.000347	0
7.457134	8.85371	-2.632759	0.000377	0
9.801027	8.228169	2.974934	0.000045	0
8.700401	9.222578	-1.436122	0.032143	0.031647
6.759203	6.267769333	1.418334667	0.012106	0.011551
5.917479667	5.222739333	1.641651	0.004758667	0.005187333
4.780167	2.845488	3.822929	0.000437	0
11.550974	10.545945	2.006984	0.000716	0
7.854621	6.756340333	2.174542	0.000958	7.73333E-05
6.884123333	7.504592333	-1.547680667	0.010377333	0.005348333
5.6732225	6.257227	-1.522202	0.0058815	0.001503
5.396762	6.1602645	-1.725166	0.002582	0.00017875
8.421005	9.875759	-2.741097	0.000884	0
3.816779	4.236442	-1.3401115	0.0286395	0.019419
9.0919515	8.46539	1.556534	0.0153405	0.0165245
7.033715	6.153814	1.8677105	0.000098	0
5.361466	6.425222	-2.324354	0.001502	0.000116
7.20574	7.8008	-1.510535	0.003837	0.000232

8.102076	7.646783	1.371061	0.001431	0
4.5884485	4.003633	1.531953	0.009722	0.0158235
10.125978	9.138238	1.983075	0.000386	0
6.537991	4.7960605	3.8183655	0.0018975	0.000701
3.923975	4.497035	-1.5191525	0.01235	0.007897
9.386748333	7.663183667	3.303014667	0.000192667	0
5.847394	6.313437	-1.3894655	0.017944	0.0158235
8.52195	8.226312	1.227428	0.021662	0.031647
8.872204	7.810246	2.087762	0.000375	0
7.825626	8.117268	-1.224033	0.038811	0.031647
12.336122	7.860888	22.242291	0.000018	0
7.684250333	6.12874	3.132118	0.004956	0.005348333
8.1010048	6.6570668	2.7688392	0.0002518	0
8.781025	9.31338	-1.4481055	0.016202	0.008482
6.91048925	5.57545675	2.737857	0.00665725	0.00791175
5.61314125	6.38802625	-1.730313	0.0034445	0.0004085
4.517827	5.197233	-1.60148	0.000932	0
7.626687	7.258725	1.290529	0.022983	0.031647
5.36867	5.799428	-1.347942	0.018681	0.007191
7.375017	6.700524	1.596036	0.000073	0
5.757478	6.139004333	-1.305035	0.013208667	0.006350333
8.938123	7.206152667	3.364419	3.96667E-05	0
7.971987	7.531048	1.357488	0.011245	0.007191
7.2087945	8.301326	-2.135525	0.0026385	0.0002415
6.34729	6.981784	-1.552393	0.001384	0
6.24583	5.318516	1.901732	0.000702	0
8.946562	9.39221	-1.361925	0.016679	0.007191
7.80762225	6.44379375	2.614329	0.00016975	0
8.498378	7.726816	1.707117	0.001811	0.000232
8.738754	7.435453667	2.470325333	0.000325	0
6.141684	5.838103	1.234205	0.023787	0.031647
10.02655	3.920051	76.484339	0.0000595	0
12.16522	9.44734	6.579056	0.000052	0
8.864162	10.14516	-2.430799	0.0027875	0.000232
6.955362667	7.592121	-1.564297333	0.00793	0.002864333

6.374276	5.5350795	1.8337495	0.002341	0.000483
6.28278075	7.1002155	-1.80106075	0.00610825	0.0038905
7.152372	7.812833	-1.580587	0.036027	0.031647
5.33008225	6.02157225	-1.6618155	0.009249	0.004642
9.091419286	7.970125143	2.225442	0.001981429	0.002223143
6.920511667	6.238269333	1.620605333	0.004142	0.002558
6.267534	6.794485	-1.440881	0.000669	0
3.907462	4.271942	-1.287418	0.005149	0.000483
5.769461667	6.583042	-1.803087667	0.001372667	7.73333E-05
8.133170333	7.128889333	2.027593667	0.000274	0
6.9651855	5.914911	2.287238	0.001743	0.0002415
9.226886	8.514388	1.638639	0.000288	0
8.242353333	9.020037	-1.72323	0.004870333	0.001079333
6.12619	6.856159	-1.658603	0.001079	0
11.744491	11.444125	1.231457	0.021421	0.031647
12.0661075	11.3698295	1.621161	0.0003675	0
4.19847475	3.36208575	1.917318	0.005564	0.0047
6.12324025	6.5033045	-1.303932	0.00635525	0.0038905
5.117605	5.463825667	-1.271708667	0.014938	0.006266667
4.533944	5.571295	-2.052456	0.001427	0
8.344123333	6.440571	4.221965667	0.000702333	7.73333E-05
7.946862	7.169926	1.713488	0.000038	0
8.232798	7.500703	1.661049	0.000547	0
9.308563	8.47137	1.786571	0.004122	0.001402
5.928239	6.784647	-1.810524	0.003443	0.000232
7.971484	9.269547	-2.458985	0.000024	0
7.138112	6.163114	1.965638	0.000995	0
6.095138	5.7218195	1.2954505	0.004184	0.001402
6.415586	7.4460496	-2.0847898	0.0014316	0.0000966
5.9436665	6.758009	-1.809367167	0.0009335	0
10.585663	9.747896	1.787282	0.000288	0
9.258016	8.430507	1.774619	0.007047	0.003006
11.391783	10.790105	1.517481	0.011039	0.007191
10.2294945	9.300265	1.9375685	0.0027065	0.000701
5.2284065	5.80703875	-1.5006455	0.00305025	0.0004085

6.47514825	7.197621	-1.660945	0.00466125	0.00087225
8.822343	6.304629	5.72674	0.000023	0
5.860919	7.202606333	-2.577015667	0.000420333	0
6.629501	5.155065	2.77875	0.000101	0
7.79665	6.91518	1.842251	0.005863	0.003006
8.139006	8.936853667	-1.744451333	0.008933333	0.003399
11.170168	10.488627	1.603852	0.00355	0.001402
4.4885225	5.7374555	-2.813987	0.0006405	0
9.569261	8.482778	2.123558	0.01577	0.015562
4.8614635	3.550118	3.066383	0.0014105	0.0002415
5.677627	6.4026275	-1.6532075	0.003111	0.000232
6.44114	7.052520333	-1.529266667	0.002876667	0.000161
4.499911	5.578326	-2.111714	0.004393	0.000483
6.166876	6.97536	-1.75137	0.000169	0
7.49123	8.048843333	-1.475835667	0.032286	0.023495
7.159143667	4.19892	9.52864	0.000202	0
6.532997	4.490481	4.119634	0.000031	0
7.242674333	5.627734	3.203406667	0.000551333	0
7.470938	8.04376	-1.48743	0.008431	0.001402
8.151778	6.695051	2.74485	0.000252	0
7.987605	7.552226	1.352266	0.012601	0.015562
4.875860667	6.860937	-4.053019667	6.63333E-05	0
9.372019667	8.109861333	2.418498	0.002648333	0.000628333
7.480377333	6.414877	2.108144667	0.002348333	0.000399333
5.695335	4.890296	1.747193	0.001715	0.000232
5.4709105	6.3908775	-1.9040815	0.000107	0
6.659710333	7.666663667	-2.069153	0.010949	0.010549
6.760461	7.391744	-1.548942	0.01056	0.003006
9.226597	7.810178	2.672946	0.000310667	0
5.604392	2.931815	6.375669	0.000053	0
6.191605	7.3750935	-2.332186	0.0000495	0
6.1278958	7.3367884	-2.3356584	0.0018746	0.0000966
4.891322333	5.975049	-2.164158667	0.004211	0.001002
4.74117225	4.03760175	1.64827925	0.0167555	0.0174905
5.602662	4.013451	3.769352	0.001312	0.000116

7.273052	6.6261	1.565856	0.001099	0
7.1023795	6.0962725	2.054062	0.0033705	0.001503
5.938865	5.275324	1.583966	0.011492	0.007191
5.69760475	3.9141505	4.99816025	0.00088975	0.00012075
10.352447	7.253953	8.565243	0.000018	0
5.8845315	6.4407035	-1.4753855	0.0012575	0
10.790746	9.193118	3.026453	0.000269	0
9.4389378	8.5533434	1.8581046	0.007343	0.0066098
9.969886	7.948434	4.05992	0.000134	0
7.973925	7.530947	1.359408	0.013988	0.015562
10.212683	8.33271	3.680683	0.000046	0
6.6921158	5.8478226	1.809317	0.0084518	0.0086492
6.728782	7.188314	-1.375096	0.001158	0
7.5945615	8.2840265	-1.6359625	0.002135	0.0002415
6.546019333	7.178955333	-1.551034667	0.001655	7.73333E-05
7.784499	7.143633	1.559935	0.010246	0.008586333
6.197263	5.753458667	1.361690333	0.004979333	0.002558
5.823673333	4.667536333	2.250799	0.000403333	0
6.7252955	7.73513	-2.016898	0.0008145	0
5.221346	5.715143	-1.408146	0.018451	0.007191
7.680195	6.6188955	2.1323495	0.0010825	0.000116
6.979507	7.663578	-1.606667	0.003004	0.000232
9.265466	8.745883	1.433541	0.009592	0.007191
8.606572	8.980831	-1.296173	0.002361	0
4.627459	3.975129	1.571704	0.001462	0
10.084316	9.319131	1.699588	0.001669	0.000232
5.655745	4.083297	2.974089	0.000066	0
5.5256325	5.0637205	1.380052	0.0051655	0.003837
9.05173	8.299739	1.684115	0.003182	0.000483
7.474361	7.0348	1.356192	0.01549	0.015562
8.018569	7.401344	1.533922	0.003621	0.001402
11.404418	10.510036	1.858813	0.01464	0.015562
7.991431	8.432738	-1.358348	0.020031	0.0159395
8.816505	8.329669	1.401368	0.013665	0.015562
4.131011	4.444313	-1.242548	0.006411	0.001402

5.036239	5.574816333	-1.455184	0.006695333	0.001546667
7.842143	6.766837	2.10717	0.000376	0
6.065615	6.7433745	-1.660715	0.021197	0.0158235
6.6536125	7.246221	-1.514401	0.0213025	0.0158235
6.934869	7.756695	-1.791008	0.014161	0.007781
8.3505915	7.1606375	2.305314	0.000647	0
4.945205	5.906542	-1.947114	0.000032	0
7.436379	7.900394	-1.379375	0.014134	0.007191
7.801104333	8.398951333	-1.525741667	0.012185333	0.010626333
5.191421	5.71464	-1.437159	0.003003	0.000232
8.5785655	9.354142	-1.713249	0.0120825	0.007781
8.628209	8.011464	1.533412	0.005913	0.003006
7.215303	8.295645	-2.114537	0.007148	0.001402
9.786971	9.121156	1.586464	0.001444	0
7.667267	9.4709425	-3.6065515	0.0007885	0
6.199053	5.339840333	1.824687	0.005914333	0.003476333
7.176382	8.461218	-2.4365495	0.001014	0
5.89237	6.277487	-1.305966	0.019488	0.007191
7.645679	8.6112515	-1.9532685	0.0004105	0
5.5634855	6.712643	-2.223473	0.0005775	0
6.59111	7.110783	-1.433629	0.008503	0.001402
5.4986885	6.6213735	-2.209731	0.0011835	0
7.087007	7.624721667	-1.454649333	0.024334	0.016203667
5.61328	6.622928	-2.0136045	0.007606	0.001402
5.382265	3.890905	2.8616495	0.005084	0.0035955
4.924367	5.5700445	-1.5672385	0.003074	0.0002415
5.961206	6.847254	-1.848107	0.003024	0.000232
3.899038	3.415605	1.401535667	0.003257667	0.002397
8.321399	8.026909	1.226451	0.014338	0.015562
9.573176	10.511384	-1.917239	0.0001985	0
10.42917	10.056543	1.294708	0.009592	0.007191
4.900918	3.359235	2.91134	0.001426	0
8.936704	9.332043	-1.315251	0.015085	0.007191
7.580147	6.52853	2.082404	0.001706	0.000238333
10.529571	9.059244	2.770847	0.000045	0

8.062593	7.065606	1.995827	0.000091	0
8.94256	7.562795	2.60226	0.000213	0
6.623388	7.128777	-1.419507	0.009152	0.001402
9.8320966	8.4652516	2.6118846	0.0013066	0.0003268
11.35639467	10.04541633	2.488439	0.000362	0
7.3975865	6.3171355	2.1405925	0.000107	0
9.6144025	9.0766905	1.451996	0.0129105	0.0113765
6.5570595	7.7249785	-2.256053	0.0004435	0
9.000428	7.533174	2.76495	0.000308	0
8.839813	7.934911	1.872416	0.003796	0.001402
8.108274333	7.179359	1.904587667	0.001840333	0.000154667
7.3052025	6.5816725	1.680589	0.0169185	0.0173265
7.667934	9.29582	-3.090598	0.000499	0
5.993599	5.436773	1.474171	0.015052	0.0158235
7.857423	8.7643045	-1.8824075	0.0138835	0.007781
7.1141755	7.7393265	-1.558989	0.01834	0.0158235
6.89262	7.364383	-1.386803	0.023621	0.015562
6.667754	6.95088	-1.216828	0.026487	0.015562
6.967542333	7.598091333	-1.557797667	0.006992333	0.002397
5.105588	5.447144	-1.267123	0.008573	0.001402
7.538182	7.918178	-1.301338	0.000736	0
6.105857	6.937509	-1.785968	0.0003255	0
5.70118	6.520814	-1.764957	0.000113	0
6.891962	7.5807575	-1.611942	0.0020125	0.000116
9.874878	8.977683	1.884180667	0.001407833	0.000233667
3.448463	4.174937	-1.65459	0.025485	0.015562
6.771722	7.738612	-1.954622	0.000019	0
10.582946	5.736772	28.763622	0.000018	0
5.989242	5.489624	1.413839	0.001958	0.000232
9.538822	6.961062	5.970121	0.000026	0
5.8620025	6.549773	-1.6179005	0.0190815	0.0165245
4.270997	4.7498785	-1.393983	0.0223705	0.015562
6.228558	5.1846875	2.3694885	0.0083545	0.007781
9.240799	9.970006	-1.657728	0.004386	0.000483
4.959536	4.535967	1.341242	0.022478	0.031647

9.249030667	7.798604333	2.744762667	0.001767667	0.000544667
5.385301333	5.899369	-1.472698	0.019174667	0.012946
5.285614333	6.075029333	-1.735310333	0.000542333	0
9.9503055	8.9760285	1.967025	0.0007035	0
8.45376	9.235702	-1.719443	0.000537	0
8.114002	7.257683	1.810413	0.000337	0
7.328844	6.368043	1.968414	0.0017155	0.0002415
6.7583085	7.948245	-2.282972	0.0007835	0
4.529574	4.956391	-1.344264	0.00737	0.001402
8.965159	9.5280835	-1.479418	0.005445	0.001503
6.445376	8.270027	-3.637712	0.0000505	0
7.7256375	6.7918225	1.938981	0.005173	0.0035955
9.0729775	8.38699975	1.615719	0.00677375	0.005343
11.15023467	10.34988267	1.755415333	0.002665667	0.000399333
6.942631	8.394195	-2.735045	0.001093	0
14.067989	10.691953	10.382175	0.000018	0
8.9478415	8.2777145	1.600557	0.0002615	0
14.762329	13.399404	2.572062	0.000018	0
8.525182667	5.639989667	7.421221667	0.000095	0
5.91718	5.14152	1.854518	0.003189333	0.001079333
5.259494	4.719515	1.472279	0.004365	0.0017445
10.504562	11.213897	-1.635051	0.008086	0.001402
6.201806333	6.988279	-1.725766333	0.001471	0
8.8114005	9.2169605	-1.324613	0.0315515	0.0236045
7.018435	6.0522315	2.03252	0.0014605	0.0002415
6.7841685	6.16655875	1.53742675	0.0077415	0.00603875
4.327647	4.840043	-1.426417	0.036923	0.031647
6.6604385	7.8506295	-2.29832125	0.0000875	0
5.137316	5.664742	-1.441355	0.004063	0.000232
10.389216	8.4703395	3.94510325	0.00005525	0
4.306401	3.705377	1.516793	0.001779	0.000232
6.819717	5.803474	2.022645	0.000025	0
7.218373	6.64303	1.491388	0.0237155	0.0236045
8.185122667	9.070208833	-1.855103667	0.0045965	0.002632333
4.7872255	5.66189	-1.8806365	0.0001575	0

5.607991667	6.158755	-1.506388	0.016912	0.012946
7.347987	9.11446	-3.402212	0.000318	0
5.798242333	4.383287333	3.254344333	0.001173	0.000161
6.667227	5.5179515	2.2756565	0.000791	0
9.774985667	8.620443	2.265039333	0.001006333	7.73333E-05
9.60477	8.579016	2.036023	0.001542	0.000232
7.760637	6.676735	2.119761	0.000093	0
6.676540667	5.405907	2.622182	0.002064667	0.001002
6.165255	6.779632	-1.530896	0.016522	0.007191
7.26786	6.5307568	1.7101706	0.010093	0.0094418
8.725243	8.0816195	1.579794	0.006372	0.0042965
7.2995375	6.3981985	1.8904445	0.005052	0.0037115
6.390081	5.5363	1.807231	0.00216	0.000232
9.17043	7.699092	2.772791	0.00011	0
5.628582	6.143325	-1.42874	0.009557	0.003006
6.987057	7.4930635	-1.426351	0.014117	0.0080225
7.0490575	5.625793	2.8803625	0.000374	0
6.854501	4.969829	3.692689	0.000021	0
10.26691067	8.382983	3.69776	0.000431	0
5.369448333	6.083207	-1.7448	0.009297	0.005264667
6.858323	6.1521175	1.650591	0.012596	0.009284
8.279022	7.19207	2.124248	0.000253	0
8.327615	6.714262	3.059622	0.000038	0
5.625964667	4.448729333	2.434466	0.002313667	0.001002
6.374134	5.44835125	1.966923	0.0030265	0.001102
7.643938	8.803275	-2.233547	0.003218	0.000232
5.5817925	6.6296455	-2.115336	0.003333	0.000701
4.309202	5.258762	-1.931284	0.00205	0
8.397719	7.989923	1.326657	0.023412	0.031647
3.705858	3.205721	1.414348	0.029434	0.031647
5.287453	6.112888	-1.772069	0.000296	0
5.74682825	6.34088025	-1.51869225	0.00848175	0.00267
8.111836	7.260187	1.804562	0.000793	0
8.848549	5.416868	10.790432	0.000033	0
5.679967	6.366631	-1.609557	0.005941	0.000483

3.647442	4.321721	-1.595798	0.003774	0.000232
6.980241	7.535859	-1.469798	0.00488	0.000483
7.1354505	7.893199	-1.691134	0.000348	0
6.435829	5.699891	1.6655925	0.0087805	0.008482
11.5261455	9.8171825	3.29069475	0.00002875	0
7.3008345	5.6441	3.3864855	0.000222	0
7.0899815	7.532879	-1.362778	0.0159805	0.008482
5.68324	6.834314	-2.220792	0.000754	0
6.467712	7.471652	-2.005469	0.000159	0
8.315677	8.774543	-1.374461	0.020802	0.007191
6.9463068	5.96458	2.022901	0.0075916	0.0077676
10.469343	9.477818	1.988285	0.000355	0
8.6340915	7.46319	2.3323955	0.0000295	0
7.210575	7.9406875	-1.6591805	0.001727	0
7.824313	8.6781415	-1.8073525	0.0002205	0
6.619403	7.163009	-1.457611	0.015186	0.007191
8.4325605	9.2902595	-1.81229	0.0014515	0
4.0265912	4.649008	-1.5873524	0.0078746	0.0026406
5.911616667	6.697025333	-1.761130333	0.017190333	0.011551
6.417816	5.85498	1.484703333	0.007318333	0.004401
4.402564	4.754108	-1.275925	0.007893	0.001402
6.3630755	6.9679995	-1.5281785	0.0068885	0.001619
6.523118	6.029325	1.408142	0.003172	0.000483
7.57443	8.896285	-2.499872	0.00003	0
6.269081	4.07259	4.583631	0.000108	0
8.281496333	6.324298667	4.780506	3.53333E-05	0
5.592129	6.207924	-1.532403	0.002011	0
4.178179	3.60689	1.485851	0.028319	0.031647
7.820207	5.648242	4.506367	0.000021	0
7.242796	7.832808	-1.505259	0.000563	0
5.2094205	4.4455505	1.7783315	0.005373	0.0035955
5.011878	4.0658535	2.06978525	0.00075025	0.000058
5.389741	6.3767805	-2.189058	0.002484	0.0002415
6.282704	6.8575324	-1.4961178	0.0055654	0.0010246
6.794892	7.402083	-1.52329	0.00303	0.000232

4.6661695	5.14752	-1.404263	0.0085355	0.0017445
6.100361	6.692842	-1.507838	0.031673	0.031647
7.41723	8.721568	-2.47143725	0.000769	0
7.384629	8.010583	-1.543232	0.006203	0.001402
8.221959	6.026311	4.580952	0.000032	0
5.717237	5.131994	1.500324	0.0051705	0.0035955
6.939961	7.577065	-1.555205	0.000111	0
9.540422	6.340645	9.188168	0.00032	0
6.168947	5.809208	1.283194	0.020913	0.031647
6.198537	6.575161	-1.2983	0.001284	0
8.650386	4.522221	17.486447	0.000019	0
7.390218	7.769219	-1.300441	0.012114	0.003006
5.752109	6.493783	-1.672115	0.00861	0.001402
6.976596333	6.142998333	1.827975	0.005408667	0.005187333
5.6769195	7.379241	-3.3639005	0.001195	0
5.311535	6.2520995	-1.9378065	0.0000205	0
5.6254015	6.1736405	-1.468184	0.005731	0.001503
5.719953	6.54171	-1.767775	0.0004615	0
6.070542	7.332814	-2.398731	0.000025	0
9.5039255	8.8863575	1.5344575	0.017453	0.019419
6.4981975	6.0277385	1.385961	0.0090975	0.009284
10.93691	10.344804	1.507446	0.021502	0.031647
9.613571	7.206123	5.30535	0.000018	0
3.530479	3.924308	-1.313876	0.008705	0.001402
5.989536667	4.922848667	2.164287	0.004532	0.002004
9.031189	7.807075	2.364822	0.0013305	0.0002415
7.955348	7.544878	1.329119	0.001601	0.000232
6.525973	6.003636	1.436279	0.001756	0.000232
5.195269	4.259309	1.913164	0.000589	0
5.9833465	5.3631075	1.545751	0.0038895	0.001503
7.7401905	6.5610055	2.2804235	0.001063	0
7.9248952	6.861812	2.1176698	0.002909	0.0012488
6.7783415	6.089696	1.645182	0.0018385	0.000232
5.614160667	4.989550667	1.548464333	0.003549	0.001079333
5.701072	6.307996	-1.523009	0.002899	0.000232

6.944061	6.370928	1.48775	0.003269	0.000483
6.60614	6.133355	1.387787	0.003758	0.001402
7.719385	8.572687	-1.807919	0.0017135	0
8.017406	8.583659	-1.480673	0.006582	0.001402
7.3489345	6.5583375	1.7308765	0.000471	0
4.851215	5.7727915	-1.978266	0.005658	0.001503
4.710177	5.129701	-1.337486	0.001129	0
6.469299	5.838302	1.5502015	0.002621	0.000483
7.866496	6.091327	3.422782	0.000035	0
7.184475	6.3086115	1.837607	0.0012745	0.000116
6.668205667	7.160364	-1.406563667	0.018309667	0.008586333
6.353114	5.290362	2.088913	0.003242	0.000483
8.032245333	7.194474667	1.810218667	0.002932667	0.001002
8.780431333	9.304208333	-1.439569	0.019667	0.011712
7.260514	6.817163	1.359758	0.002046	0.000232
6.070336	6.794145	-1.651537	0.000108	0
5.676091	6.212766	-1.451631	0.019824	0.016065
6.887312333	5.407119333	2.916080333	0.000226	0
6.797445	6.273466	1.437916	0.001979	0.000232
6.6284945	5.57519625	2.08706225	0.00052	0
5.759171	6.424032	-1.585416	0.022083	0.015562
9.555717	9.14304	1.331153	0.01388	0.015562
12.179974	10.494059	3.217445	0.000018	0
8.114509	8.784241	-1.590777	0.005418	0.000483
6.143495	6.490869	-1.2740345	0.040845	0.031647
4.945647	6.358985	-2.663527	0.000084	0
8.653068	9.297718333	-1.564512333	0.004165667	0.000628333
5.51755575	4.582883	1.96656625	0.00465475	0.002728
8.036429	6.3845425	3.165063	0.0001945	0
3.947617	4.442551	-1.409257	0.027997	0.015562
6.05903	6.468647	-1.328333	0.013463	0.003006
8.3069255	7.737944	1.487541	0.0022615	0.0002415
8.397749	7.0934645	2.50993	0.00009325	0
7.107160167	6.210872167	1.9191235	0.0074165	0.007900167
8.5722714	7.2498848	2.5061648	0.000537	0.0000464

10.08008033	7.519249333	5.967252333	0.000057	0
7.069607	7.905177	-1.784562	0.000206	0
9.75153	7.682435	4.196232	0.000018	0
8.564337	7.582305	1.9806255	0.0000205	0
5.456701	6.148249	-1.615016	0.003784	0.000232
7.022942	7.367628	-1.269875	0.003777	0.000232
7.138242	7.751262	-1.529458	0.003581	0.000232
6.920134333	7.633669	-1.640846	0.005936333	0.001469333
11.22192	10.461944	1.693462	0.000456	0
7.635528	7.095402	1.454099	0.000653	0
6.109536	4.465097	3.126263	0.000524	0
9.2376175	5.47972	14.220039	0.000039	0
6.921435	6.310538	1.527208	0.024371	0.031647
7.38848	5.638234	3.36416	0.000595	0
6.536341	5.552502	1.997579	0.002572	0.000701
5.446810333	6.34073	-1.909279333	0.004327667	0.001002
4.355828	4.880811	-1.438916	0.002179	0
5.3389895	5.868959	-1.4462165	0.0024135	0.0002415
8.839852	9.665349	-1.772146	0.000276	0
8.240108	8.7412895	-1.4211685	0.0065825	0.001503
6.013667	6.456854	-1.359605	0.012079	0.003006
7.907144	6.3461225	2.9655815	0.0002075	0
8.5154902	6.966925	2.954436	0.0003122	0
5.115538	5.403123	-1.220595	0.04328	0.031647
7.520917	6.996923	1.43793	0.003211	0.000483
7.309836	4.8686815	6.14592	0.000035	0
4.7584735	5.824355	-2.145314	0.000636	0
8.791002	9.355532	-1.478907	0.011719	0.003006
5.647331	5.200559	1.362987	0.017431	0.015562
8.510291333	9.236143667	-1.661502333	0.000255667	0
6.309606286	5.196520571	2.241599	0.001039429	0.000233429
6.315383	6.722308	-1.325856	0.012313	0.003006
7.009591	8.126361	-2.2495585	0.000084	0
8.50007	8.040714	1.374928	0.004644	0.001402
4.9492435	5.4055405	-1.381241	0.025189	0.019419

7.7485315	5.251162	6.3758415	0.0001505	0
6.542672	5.810486	1.7060265	0.00279625	0.00179775
6.57291	7.114942	-1.456022	0.015469	0.007191
7.488878	6.40308075	2.17531075	0.0030955	0.00179775
9.193845	8.645712	1.462193	0.010426	0.007191
7.474428333	9.047322667	-2.988285667	0.001802	0.000161
5.409649	5.7873285	-1.30111	0.006413	0.001619
5.414178	6.197129	-1.720647	0.001854	0
5.824066	6.574653	-1.682476	0.000053	0
5.192071	5.748234	-1.470354	0.004474	0.000483
4.540614	4.825442	-1.218265	0.019176	0.007191
5.256755	5.7729	-1.430128	0.014881	0.007191
4.2390605	4.90946	-1.605063	0.008655	0.0037115
6.551361	7.166584	-1.531794	0.000169	0
5.585813	6.109558	-1.437683	0.009371	0.003006
6.432476	7.221083	-1.746598	0.0005735	0
5.740981	6.539885	-1.739779	0.000494	0
7.000488	6.625562	1.296773	0.025078	0.031647
4.002098	5.191309	-2.280279	0.000106	0
5.589059	6.001961	-1.331361	0.027433	0.015562
5.639393	4.301047	2.528614	0.001157	0
3.33445	3.777337	-1.359322	0.020975	0.015562
5.8437535	6.392535	-1.4748165	0.0012555	0
6.758118	5.660593	2.1745475	0.001286	0.0002415
5.722616	6.360932	-1.556511	0.000257	0
5.298758	6.437042	-2.201191	0.000618	0
6.097574	6.569326	-1.386792	0.009266	0.003006
5.710759	6.101111	-1.310714	0.014558	0.007191
5.406577	5.836684	-1.347333	0.001477	0
4.609331	5.42101	-1.755254	0.00594	0.000483
5.0552055	5.4752355	-1.342313	0.0059455	0.001503
6.241127	7.080202	-1.788903	0.000032	0
7.054065	6.686818	1.289889	0.01376	0.015562
7.823969667	8.300782	-1.392268	0.007787667	0.001402
4.980037	5.476684	-1.4111295	0.008911	0.002204

6.639269	7.01385	-1.296463	0.028864	0.015562
5.179021	5.465501	-1.219661	0.03835	0.031647
6.403466	7.924791	-2.870546	0.000263	0
5.772187	7.045082	-2.466404	0.0000195	0
4.62935	4.992393	-1.286135	0.004844	0.000483

Mean Signal of Group Sepsis(GSE64457)	Mean Signal of Group Control(GSE64457)	Fold Change(GSE64457)	p-value(GSE64457)	FDR(GSE64457)
5.797497	6.52609	-1.657022	0.005354	0.015015
4.586383	4.229916	1.280287	0.012568	0.048139
8.0891735	7.206919	1.8434745	0.000025	0
4.549935	2.9547645	3.337991833	0.005253667	0.019876
5.51349825	4.2860795	2.430142	0.0044205	0.0128895
3.374834	4.543468	-2.247988	0.000464	0.002333
7.084918	6.413052667	1.593349333	0.006654333	0.024091667
3.069875	2.747228	1.250623	0.019591	0.048139
2.745936	5.508647	-6.786705	0.000024	0
5.723908667	4.658711333	2.100518333	0.000731667	0.004691
8.501079	7.806636	1.618328	0.004665	0.0174755
8.910644	8.318569	1.5134015	0.0075725	0.025236
4.633812	5.74074	-2.153866	0.011174	0.033306
11.289345	10.914374	1.296813	0.000645	0.003863
5.078247	2.571253	5.684347	0.002993	0.015015
4.570813	6.55274	-3.950202	0.000042	0
8.08473075	6.4189775	3.19433525	0.00003825	0
5.195899	4.895305	1.231651	0.005466	0.022439
5.709956333	4.878950333	1.782326333	0.003139667	0.012476667
9.346898	7.916306	2.695573	0.000433	0.002333
10.552119	9.935318	1.53347	0.000124	0.001086
5.983989	5.4821465	1.4271935	0.002742	0.013151
10.510893	10.1629795	1.2727185	0.015023	0.048139
2.311218	2.79505	-1.398453	0.002392	0.009121
7.474784	6.4793305	2.0104965	0.001185	0.00587
9.4344405	10.249438	-1.759509	0.0002135	0.001645
6.52195	7.823633	-2.465163	0.00014	0.001086
3.331438	2.005699	2.506612	0.014228	0.048139
6.185421	7.182204	-1.995544	0.000182	0.001645
7.799065	8.50893	-1.635651	0.010319	0.033306
7.911923	6.797657	2.164848	0.001173	0.00587
6.59506	7.157059	-1.476314	0.002433	0.009121
7.277059	8.034608	-1.69542175	0.00277475	0.01011475

6.440276	6.0205	1.33772	0.012359	0.033306
10.808107	8.704899	4.296639	0.000322	0.002333
10.373929	9.048798	2.505557	0.000081	0
5.747893	6.678019	-1.905442	0.000068	0
6.927921	6.28812	1.558114	0.014452	0.048139
8.508608	8.1058145	1.3222575	0.017962	0.048139
3.347524	5.380797	-4.093325	0.00525	0.015015
7.2007965	5.0827035	4.51627925	0.00223575	0.00956025
5.24862825	4.0021985	2.40919525	0.00137725	0.00638775
5.314249	5.867871	-1.467766	0.016402	0.048139
5.57766	6.085508	-1.421927	0.008001	0.022439
6.050821	5.113194	1.915374	0.004066	0.015015
8.6912635	8.1083	1.5019865	0.001215	0.00587
10.163902	9.790412	1.295483	0.002867	0.015015
6.442562	5.58831	1.81642175	0.009665	0.0324415
8.872115	8.247157	1.542166	0.012835	0.048139
5.894065375	4.496122125	2.694163125	0.00040275	0.002497375
4.8311345	5.5264715	-1.629795	0.011041	0.031577
6.022833	5.515248	1.421668	0.005765	0.022439
7.885169167	7.101979667	1.7443335	0.005880667	0.019527167
7.34009	6.137778	2.3233985	0.0062455	0.0212135
5.135415	6.732069	-3.037291667	0.000642	0.003863
2.904644	3.297581	-1.313064	0.010968	0.033306
10.42969033	10.11849667	1.241272	0.013348	0.043194667
8.072207	6.9298435	2.207588	0.001316	0.0074955
4.854242	3.977035	1.836816	0.011587	0.033306
6.5266625	5.245405	3.016345	0.0059595	0.016653
7.118891667	7.906772333	-1.737355667	0.002084	0.008037333
5.290423667	4.480028	1.755743333	0.014518333	0.043194667
8.7925885	9.2440095	-1.3676435	0.0121305	0.033306
6.496171	4.84727	3.135946	0.000068	0
5.245094	2.8917605	5.34923775	0.00118	0.0071
8.46097	7.725984	1.664381	0.001254	0.00587
6.572883333	7.366823167	-1.750078667	0.002317167	0.007800167
3.777913	4.6441395	-1.8480995	0.0088915	0.02594825

4.912702	4.23092	1.60412	0.000265	0.001645
8.697788	7.827602	1.827899	0.0038	0.015015
6.178335	4.710471	2.766121	0.018072	0.048139
5.7377625	6.613632	-1.8351945	0.000102	0.000543
11.205784	10.827507	1.2999145	0.0045615	0.0212135
2.329673	4.351042	-4.681274	0.0000855	0.000543
4.956913	6.64326	-3.218407	0.001312	0.00587
6.7001475	8.0566275	-2.5831845	0.002827	0.012068
6.3347595	7.6966275	-2.5753925	0.003709	0.012068
5.334433667	5.848409333	-1.430679	0.012	0.033306
3.863147	4.573626	-1.636347	0.02103	0.048139
6.675008	6.17562	1.413614	0.013613	0.048139
9.646517	10.178746	-1.446161	0.004157	0.015015
6.1070635	7.032448	-1.917523	0.0009	0.0041015
4.782686667	5.866137	-2.140327	0.002992333	0.011807667
9.674587	6.912336	6.784544	0.000024	0
3.888244	4.40109	-1.426862	0.020968	0.048139
2.686343	2.416871	1.205367	0.016903	0.048139
4.301316	4.75355	-1.3681575	0.0125925	0.035289
9.395791	6.086917	9.909925	0.000027	0
8.169722	5.763553	5.30065	0.000666	0.003863
4.617177	2	6.135484	0.000327	0.002333
5.695617	4.6603965	2.052366	0.000785	0.0041015
12.0673	11.763048	1.234779	0.000547	0.003863
5.630088	6.4368705	-1.7493845	0.0030045	0.012068
7.151712667	6.477153333	1.599504333	0.005259333	0.016824
7.261417	5.908428	2.554409	0.00169	0.009121
4.674092	4.151457	1.436577	0.01684	0.048139
9.523973	8.37486	2.217774	0.000128	0.001086
3.0959715	4.2210085	-2.196415	0.004383	0.012386
7.255627	6.280556333	1.981697	0.000334333	0.001836
3.361135	3.783012	-1.339669	0.015239	0.048139
5.2747175	4.1286095	2.2429245	0.0016255	0.008674
6.280117	7.258616	-1.970414	0.000059	0
7.15406	6.3143255	1.846994	0.0083775	0.0270045

3.284572	3.873253	-1.503872	0.00826	0.022439
4.160951	4.780246	-1.536124	0.002973	0.009121
2.839762	3.296407	-1.372346	0.010187	0.033306
10.746922	10.322598	1.341943	0.008392	0.033306
5.4425585	5.8812595	-1.355548	0.0037595	0.01578
5.160768	8.011346	-7.212896	0.000046	0
7.18129	3.7074525	11.487385	0.0048335	0.018727
10.216275	5.766063	21.859856	0.000024	0
2.676697	3.784891	-2.155756	0.002178	0.009121
4.855109	5.732707	-1.837313	0.001599	0.00587
9.9708315	9.534019	1.353615	0.0076115	0.0270045
12.625621	11.926618	1.623383	0.000053	0
5.717814	5.350912	1.28958	0.019528	0.048139
2.044235	2.308571	-1.201083	0.00438	0.015015
3.196237	2.535497	1.580893	0.017894	0.048139
9.576341	8.80090725	1.7176725	0.0000305	0
11.035939	11.53308533	-1.414103	0.005847667	0.01543
4.663401	5.346142	-1.605187	0.002794	0.009121
9.007654	5.403034	12.1648575	0.000025	0
6.374272	5.35806	2.022602	0.000399	0.002333
6.792492	7.383989	-1.50681	0.009781	0.033306
6.610048	7.087095	-1.391891	0.019237	0.048139
8.728233	7.457757	2.475543	0.000842	0.0045605
4.238543	4.576373	-1.263855	0.015551	0.048139
3.029425	5.109787	-4.229133	0.000183	0.001645
9.715866	8.944928333	1.710545667	0.000161333	0.000777667
9.226285	8.282946	1.922973	0.013444	0.048139
10.308751	10.04562	1.200081	0.003213	0.015015
3.199173	3.93528	-1.665676	0.000269	0.001645
3.878592333	4.586443333	-1.663261333	0.013629667	0.035133
8.276762	8.704237	-1.344878	0.00321	0.015015
5.600116	6.372369	-1.707936	0.014186	0.033306
5.510389	4.794259	1.642769	0.008771	0.033306
6.855086	7.634198	-1.716074	0.003275	0.015015
3.768444	3.064921	1.628477	0.00233	0.009121

6.78288875	5.92596425	1.8242325	0.00888975	0.02781725
6.963213714	7.893724143	-1.932943	0.000101143	0.00047
5.758383	6.631274	-1.831329	0.006008	0.022439
5.756094	6.650856	-1.859303	0.005214	0.015015
3.691919	4.49422	-1.74388	0.016744	0.048139
8.647555	7.7924	1.808952	0.005492	0.022439
8.398535667	7.408479333	1.988267333	0.000387667	0.002384333
8.122971	8.579002	-1.371763	0.001309	0.00587
11.037547	9.033339	4.011685	0.000192	0.001645
4.58846625	5.66673575	-2.12157625	0.00239925	0.00847325
8.09761025	7.127686	1.99284625	0.0049205	0.01953625
2.291271	2.686928	-1.315541	0.011353	0.033306
7.453815	6.538623	1.885819	0.000115	0.001086
4.211183	5.1308015	-1.932688	0.0014635	0.0045605
3.158302	3.896698	-1.66832	0.004028	0.015015
3.29777625	4.90990075	-3.4471275	0.0008355	0.00401925
5.3619544	6.4134414	-2.0982724	0.0002598	0.0017624
7.853555	4.254119333	12.227062	0.000711	0.004366333
5.979782	7.487634	-2.843881	0.001891	0.009439
9.678453	8.886255	1.731711	0.001181	0.00587
3.844581	2.3164235	2.892891	0.0002165	0.001645
8.411607	6.180959	4.693446	0.004112	0.015015
4.377317333	2.886346333	2.828239333	0.000506	0.001956667
7.967041	8.339399	-1.294467	0.009184	0.033306
2.9857345	3.8279715	-1.793429	0.003237	0.012068
5.1716635	5.9146635	-1.6829345	0.0032945	0.0112195
6.130668	5.430688	1.624482	0.003064	0.015015
6.048769333	4.630044333	2.692054333	0.000048	0
4.172065	3.061714	2.158982	0.001787	0.009121
5.324126	6.199576	-1.83458	0.000461	0.002333
2.545103	3.067572	-1.436411	0.013121	0.033306
5.82162	6.394688	-1.489519	0.001216	0.00587
6.2116855	6.6464005	-1.35174	0.020095	0.048139
8.7655	9.206905	-1.357926	0.016259	0.048139
6.455577	6.922159	-1.381831	0.00629	0.022439

10.090661	9.607859	1.397456	0.01018	0.033306
4.733791	3.69504	2.0545505	0.0061415	0.0212135
6.684783	7.512008	-1.774269	0.000213	0.001645
8.38281	7.698436	1.607004	0.001124	0.00587
5.306188	5.861393	-1.469378	0.00088	0.003863
3.6419225	2.233564	2.7088005	0.0063775	0.022439
3.188753	4.162505	-1.963942	0.000486	0.002333
7.199323	6.5215225	1.6003195	0.0013035	0.0074955
5.285377667	4.569443667	1.663008	0.001464667	0.008249333
6.896373	6.524982	1.294218	0.015115	0.0407225
9.316695	8.0903	2.339816	0.000069	0
5.633461	5.976479	-1.268408	0.013963	0.033306
3.996089	4.579015	-1.497884	0.017407	0.048139
5.904986	5.044742	1.815345	0.000389	0.002333
8.307423	7.427107	1.840779	0.000622	0.003863
3.234973	2.062545	2.253908	0.006658	0.022439
8.808302	7.949573	1.813439	0.009514	0.033306
2.059725	2.6875445	-1.54578	0.0003845	0.002754
9.854564	9.364907	1.40411	0.001199	0.00587
8.60048	6.527736	4.206861	0.000281	0.002333
5.73993	6.249271	-1.4234	0.019417	0.048139
7.864756	8.265211	-1.319924	0.007132	0.022439
8.652367	8.226774	1.343124	0.010548	0.033306
7.341642	7.892561	-1.465018	0.015638	0.048139
8.876433	9.243825	-1.290018	0.015242	0.048139
10.611822	10.272197	1.265428	0.018374	0.048139
2.998355333	3.909802	-1.9181	0.005900333	0.016099
3.294959	4.253307	-1.943084	0.007245	0.022439
6.735381	5.156213	3.060789667	0.000162	0.000777667
6.2162275	4.690329	2.927601	0.0000925	0.000543
3.0804735	3.6988565	-1.535907	0.001824	0.006492
4.300627	3.222109	2.192635	0.0056275	0.0241605
3.920183	5.163739	-2.367815	0.000274	0.002333
4.399663	3.383674	2.022288	0.006023	0.022439
5.512232	4.682169	1.777762	0.003642	0.015015

3.9098185	4.6339935	-1.6533835	0.011401	0.035289
8.472128333	7.041637667	2.702327667	7.36667E-05	0.000362
7.5076535	6.9341785	1.4883535	0.005645	0.0241605
6.35601625	5.0956955	2.50553275	0.0020415	0.00938625
8.751056	7.085238	3.172937	0.000044	0
9.0002995	7.9670425	2.0484625	0.000589	0.0041015
3.961082	3.511377	1.365761	0.011273	0.033306
7.09181	6.355508	1.6659	0.020826	0.048139
6.692178	5.213374	2.8330405	0.003211	0.012068
2.3594045	3.518604	-2.2510925	0.0048175	0.0185845
2.626582	3.1510315	-1.4385925	0.0028405	0.0104425
7.833992	6.171598	3.165412	0.003438	0.015015
5.145404	4.577316	1.482557	0.010732	0.033306
6.58133	5.91158	1.590797	0.012965	0.048139
8.990089	8.511083	1.393783	0.000907	0.00587
3.8237755	4.566849	-1.67387	0.0020285	0.0074955
3.371572	2.860572	1.425038	0.00039	0.002333
2.064613	2.346829	-1.216062	0.020819	0.048139
6.927388	2.042654	29.542786	0.000024	0
5.713821	6.215358	-1.415721	0.009013	0.022439
2.000791	2.268458	-1.203859	0.003976	0.015015
3.190823	3.972995	-1.719718	0.000094	0.001086
6.985164	6.240406	1.675694	0.006323	0.022439
8.074313	7.0888815	1.980329	0.010471	0.0407225
6.05481	5.368778	1.608852	0.01673	0.048139
8.596889	7.803608	1.73538	0.000555	0.003098
2.087124	2.552007	-1.380206	0.021482	0.048139
12.1573125	10.632781	2.883898	0.000024	0
2.793574	3.246502	-1.368815	0.017871	0.048139
3.992352	5.2491105	-2.389812	0.005115	0.018727
8.661607	8.193033	1.383741	0.019692	0.048139
7.949486	7.391798	1.471909	0.000158	0.001086
2.077119	2.394469	-1.246039	0.012753	0.033306
6.003462	5.155216	1.800311	0.012685	0.048139
8.3366475	10.1349725	-3.513914	0.0005015	0.002754

9.628048	9.16474	1.378699	0.010688	0.033306
6.786249	7.286419	-1.4147095	0.0133105	0.035289
10.73524933	9.520518	2.348925333	0.004127333	0.015737
7.565909	6.581442	1.978582	0.003179	0.015015
11.708311	11.2520505	1.371988	0.014442	0.0407225
8.041343	7.646549	1.314755	0.012768	0.048139
4.906546	2.701081	4.612232	0.008824	0.033306
2.579507	2.890251	-1.240347	0.001085	0.00587
2.1995525	3.8046905	-3.3682205	0.000887	0.0045605
2.939208	4.271352	-2.517765	0.000856	0.003863
2.392503	2.975258	-1.497707	0.002679	0.009121
7.916553	6.531137	2.612472	0.000299	0.002333
8.2999685	7.6192965	1.6028885	0.00112	0.00587
5.4677082	6.2544248	-1.7435014	0.0012984	0.0061888
5.32911	6.124983	-1.736128	0.002032	0.009121
4.321506	5.732483	-2.659171	0.000032	0
5.7889725	6.423375	-1.5644725	0.0046325	0.0141545
9.195449	6.993988	4.599449	0.000024	0
5.861637	4.056835	3.49381	0.000139	0.001086
10.28593117	9.015037833	2.416109	3.93333E-05	0
8.3157342	7.1865678	2.1942818	0.001576	0.0075464
2.223896	2.502877	-1.213338	0.020424	0.048139
6.164154	5.623816	1.454313	0.010555	0.033306
4.342261	4.811871	-1.384735	0.00591	0.022439
6.293642	5.557139	1.666132	0.003094	0.015015
3.769411	2.803189	1.953718	0.002888	0.015015
9.09012	9.83658	-1.677672	0.012414	0.033306
2.50507	2.202345	1.233472	0.005697	0.022439
9.0994	8.3973195	1.6270765	0.0016935	0.00833
8.028341	7.520115	1.422301	0.00013	0.001086
6.068311	7.444121	-2.595136	0.001089	0.00587
3.082395	3.8721055	-1.743472	0.006278	0.0241605
5.76848	6.303911	-1.449375	0.004034	0.015015
3.081069	3.978922	-1.863291	0.002565	0.009121
3.022345	3.704644	-1.604695	0.004543	0.015015

4.58511	5.080153	-1.409362	0.010408	0.033306
6.267404	6.859062	-1.506978	0.003667	0.015015
7.232179	6.477704	1.687017	0.010227	0.033306
2.189654	2.629311	-1.356281	0.017975	0.048139
5.596289	2.679192	7.553247	0.000032	0
6.34429	5.616121	1.656535	0.000834	0.00587
3.097424	3.8985	-1.7424	0.001915	0.009121
6.54274825	4.19178975	6.0444115	0.00199125	0.0083265
2.691683	2.090622	1.516832	0.018954	0.048139
3.5693875	4.174188	-1.521562	0.0046785	0.01578
2.321376	3.5516835	-2.402426	0.0000365	0
5.2877665	6.095162	-1.752762	0.004221	0.015015
**10.914821**	**8.5344335**	**5.2608825**	**0.000024**	**0**
2.22433	2.848985	-1.541842	0.005062	0.015015
4.877613	5.654500667	-1.722199	0.004468333	0.015525
9.439225	8.812336	1.544231	0.005045	0.022439
6.062206	4.775363	2.439935	0.001767	0.009121
4.478233	5.166371	-1.611203	0.000116	0.001086
8.95477	8.307261	1.566461	0.015121	0.048139
3.938279	4.78328	-1.796265	0.001548	0.00587
5.4795765	3.788816	3.2957725	0.0005845	0.0041015
5.7119545	4.182611	3.021547	0.007119	0.02863
6.275064	5.553928	1.64848	0.00205	0.009121
2.493832	2.091569	1.32158	0.016318	0.048139
10.717716	9.873127	1.795753	0.00198	0.009121
7.9066885	5.9670345	3.8890385	0.0005845	0.002935
5.7785485	6.7095425	-1.9393345	0.005326	0.019588
10.453532	9.874531	1.493815	0.000183	0.001645
6.487317	4.97112	2.86036	0.000076	0
2.357252	3.362151	-2.006804	0.00004	0
2.62001	4.020441	-2.639805	0.000388	0.002333
3.831264	4.379028	-1.461817	0.002548	0.009121
2.276145	2.846225	-1.484606	0.011182	0.033306
3.463498	5.221585333	-3.472007333	0.008246	0.025482667
3.2851255	5.86174	-6.0593515	0.000059	0

8.275657	7.812062	1.379543	0.0006065	0.0041015
8.1025345	8.893295	-1.7302655	0.011355	0.031577
6.651162	7.312672	-1.581738	0.004174	0.015015
4.921604	5.646813	-1.65314	0.0027	0.009121
4.477371	5.271559	-1.734101	0.002547	0.009121
4.719928	5.491612	-1.707262	0.008575	0.022439
9.27885375	10.1194935	-1.82538825	0.00026075	0.00167725
7.533228	8.5917055	-2.0833595	0.0001345	0.0013655
8.085083667	8.458754	-1.297529	0.004347667	0.013560333
5.9809975	6.516245	-1.456033	0.003809	0.012042
8.946883	6.416147	5.950183	0.0000635	0
7.002185	7.764044667	-1.701832667	0.002481667	0.010002
5.90606525	2.733389	10.096352	0.0003965	0.00228025
8.014703	7.351124	1.584007	0.001298	0.00587
3.264293	2.841918	1.340132	0.011554	0.033306
3.642819	2.757712	1.8469	0.001895	0.009121
5.047393	6.610614	-2.955128	0.000213	0.001645
11.618433	11.325581	1.225059	0.00784	0.033306
3.887194	4.436914667	-1.475269333	0.007701667	0.026566333
6.337595	8.529573	-4.569314	0.000119	0.001086
6.74849	3.467251	9.72191	0.000042	0
12.037475	10.931988	2.151716	0.002044	0.009121
7.506093	10.362978	-7.244496	0.007681	0.022439
8.808613	8.529652	1.213321	0.001382	0.00587
5.022052	3.5524145	2.770894	0.000463	0.002935
6.943373	7.795935	-1.805704	0.011622	0.033306
9.309095	7.032564	4.845116	0.000212	0.001645
10.339069	8.676714	3.165326	0.00014	0.001086
6.454673	5.825553	1.546622	0.003681	0.015015
11.00573	9.4376345	2.9730455	0.0007545	0.0041015
9.939983	9.07996	1.815067	0.000066	0
2.213574	2.6236615	-1.3289965	0.009607	0.02863
4.300256	3.173388	2.183841	0.020835	0.048139
4.649002	5.31099	-1.5875445	0.006333	0.022439
6.072451	3.8133225	4.813271	0.00005	0

7.820670333	6.004675333	3.620341667	0.000131333	0.000777667
9.719841	9.086802	1.550828	0.016432	0.048139
8.6938845	9.7631605	-2.0983855	0.015033	0.048139
3.115930333	4.048019	-1.909279	0.012776333	0.039572333
5.595932	3.670879	3.797508	0.010719	0.033306
12.013897	11.718151	1.22752	0.01033	0.033306
3.4171435	3.98037	-1.489224	0.011115	0.0270045
5.660167	4.731886	1.903008	0.004091	0.015015
5.326967	3.920354	2.651142	0.010709	0.033306
2.493802	2.197039	1.228385	0.01727	0.048139
5.09926325	4.4466065	1.595923	0.005589	0.01829675
3.9201665	4.9909165	-2.131635	0.0029055	0.008674
6.272788	5.4679205	1.7473265	0.002991	0.015015
4.508344	5.097741	-1.504618	0.016247	0.048139
4.0606	2.946348	2.1688435	0.010366	0.02863
6.095182	5.527642	1.481994	0.019824	0.048139
4.596165	5.367673	-1.707053	0.007996	0.022439
8.959603	9.554175	-1.519179	0.0010205	0.0051035
3.820197	5.080085	-2.394772	0.000329	0.002333
8.347516	7.27446	2.103885	0.002642	0.015015
4.8810425	5.7145995	-1.786822	0.0013995	0.0074955
2.383587	3.49276	-2.157219	0.000041	0
3.885835	2.868594	2.024044	0.005622	0.022439
3.744555	3.172134	1.487017	0.000553	0.003863
7.3595475	6.6897045	1.5943235	0.00099	0.0051035
4.734906	4.340203	1.314672	0.001002	0.00587
4.127217	4.811092	-1.606449	0.012679	0.033306
6.284603	7.21896	-1.911039	0.003028	0.009121
2.573009	2.128232	1.361103	0.00655	0.022439
8.515222	8.075758	1.3575465	0.005853	0.022439
3.07811	3.69311	-1.531558	0.005364	0.015015
2.722105	3.681792	-1.944887	0.000142	0.001086
2.201715	2.483642	-1.215818	0.015021	0.048139
6.354029	5.718402	1.555120667	0.006858333	0.018003
3.891441	5.136707	-2.3959625	0.002841	0.0117625

5.4424135	5.945142	-1.4169005	0.0124675	0.035289
5.4134765	3.8069335	3.492159	0.0123835	0.035289
9.207418	8.761392	1.362282	0.008551	0.033306
10.0763385	9.350225	1.6560575	0.0003085	0.0019315
3.650005	2.434175	2.326332	0.0097665	0.0270045
2.396009	3.020337	-1.541493	0.008179	0.022439
8.610937	8.086122	1.43875	0.005081	0.022439
10.089238	8.407722	3.20765	0.000029	0
4.309740667	2.894890667	2.857557	0.004206333	0.012198667
8.6713865	9.211602	-1.454318	0.015624	0.0407225
5.901383	3.931176	3.918244	0.002218	0.009121
7.54456	6.6909755	1.814714	0.007017	0.0270045
4.268975	5.278145	-2.015038	0.0007295	0.003863
8.456861667	6.865446333	3.023314	0.000741	0.004275333
10.534891	10.189065	1.270879	0.006094	0.022439
2.259948	2.902732	-1.561339	0.006313	0.022439
7.578044	7.040529	1.451471	0.01524	0.048139
5.9966385	5.012741	2.1073585	0.0000445	0
5.229784	6.279761	-2.070496	0.001902	0.009121
8.614116	7.9978	1.532956	0.000519	0.003863
8.274142	8.7673755	-1.408726	0.00295	0.012068
9.610386	8.759319	1.803835	0.000052	0
8.807693	7.980708	1.773975	0.001515	0.009121
5.950607	4.986221	1.951233	0.008965	0.033306
9.941192	8.852631	2.126618	0.008692	0.033306
8.286508	7.5019915	1.7254695	0.000485	0.002935
4.684081	5.21301	-1.444765	0.011382	0.035289
2.123801	2.78538	-1.581813	0.004357	0.015015
3.7235015	4.271745	-1.4635605	0.008541	0.0278725
7.248925	8.416576	-2.246455	0.003697	0.015015
2.688221	3.552237	-1.820512	0.00141	0.0051035
8.1257565	6.3162155	3.517079	0.0029325	0.012068
4.4312435	3.7101435	1.6565575	0.000651	0.002935
3.077942	4.292793	-2.321168	0.001097	0.00587
2.525973	3.306325	-1.71755	0.02024	0.048139

8.1540035	7.4425195	1.6378135	0.0051025	0.019588
5.083226667	2.082934	8.245811667	0.006343	0.023586667
9.165197	8.7093055	1.374063	0.00204	0.009121
7.602568	5.807049	3.471403	0.000067	0
3.682399333	5.567696667	-3.725389	0.000218667	0.001688
9.701141333	8.667793	2.050908667	0.000117333	0.000910333
3.8954178	4.6231368	-1.6727936	0.002849	0.0081176
4.903032	4.530984	1.294188	0.003842	0.015015
9.165043	8.249269	1.88658	0.003746	0.015015
5.16684	5.564159	-1.3186095	0.0054655	0.018727
12.188057	9.323764	7.281793	0.000025	0
6.557804	5.919172	1.556852	0.018749	0.048139
8.577889	8.124457	1.369294	0.007336	0.022439
4.29265875	4.90042575	-1.541558	0.0038985	0.0100655
6.628845333	5.428638	2.326829667	0.001490333	0.007323667
3.9646145	5.2190005	-2.500246	0.00452525	0.01494375
3.903859	5.19518	-2.44752	0.005368	0.015015
6.753411	6.054272	1.623535	0.003698	0.015015
2.081016	2.934662	-1.807062	0.000112	0.001086
5.346314	4.817981	1.442262	0.015093	0.048139
3.783305	4.525968	-1.673261	0.007336	0.022439
8.866114667	7.683712	2.271458	0.000274667	0.002198
7.0017045	5.8837685	2.1708615	0.000216	0.001645
7.010178	7.638695	-1.545975	0.005071	0.015015
3.66281	4.303744	-1.559338	0.013128	0.033306
4.427795	3.982096	1.361974	0.014819	0.048139
6.83757725	7.59762725	-1.70311575	0.0079015	0.02492175
4.4172865	2.7423415	3.301003	0.0057675	0.019588
7.971269	7.544202	1.344498	0.018185	0.048139
7.8339135	6.905741	1.904229	0.0017135	0.008674
8.408644	8.054161	1.2793695	0.00729	0.0278725
5.6232005	3.208079	5.3733815	0.0038385	0.018727
10.633616	9.057126	2.982434	0.000024	0
6.124871	9.1359355	-8.0981045	0.0000415	0
5.917076	6.532007	-1.538238	0.001688333	0.006961667

5.21126975	4.37531325	1.81940975	0.0052875	0.019546
4.0952035	5.076383	-1.990436	0.002494	0.0080505
8.417852	9.136285	-1.645394	0.000146	0.001086
2.648166	3.589928	-1.920872	0.000025	0
8.763002167	6.7486055	4.254538167	2.68333E-05	0
6.4875075	5.5841085	1.9262635	0.004586	0.016653
5.061884	5.554293	-1.406792	0.006741	0.022439
3.021201	3.777144	-1.688735	0.012931	0.033306
5.116168	5.7321745	-1.534543	0.0023165	0.0104425
7.525423333	6.900761	1.543583333	0.001094333	0.006953667
5.9191015	4.2612875	3.1857035	0.002647	0.0104425
7.459456	7.093243	1.288965	0.001697	0.009121
5.738999667	6.339142333	-1.518641333	0.002429333	0.008593667
2.931547	3.770612	-1.78889	0.000061	0
9.842313	9.457396	1.305784	0.005192	0.022439
7.67529275	6.90800575	1.746839	0.00094475	0.00457625
4.5937715	2.347974	5.7206945	0.000202	0.0011665
4.542823	5.249871	-1.632461	0.004455	0.015015
2.714645	4.178422	-2.758297	0.000099	0.001086
2.167266	2.828214	-1.581121	0.000872	0.003863
7.914429333	6.338461333	3.032185667	0.000803667	0.004366333
6.80161	6.244699	1.471116	0.020826	0.048139
5.956899	5.537634	1.337245	0.005199	0.022439
9.595242	9.170724	1.342124	0.004057	0.015015
3.64297	4.228871	-1.500975	0.02113	0.048139
6.22018	6.741185	-1.434955	0.005326	0.015015
7.224572	6.486421	1.668036	0.008515	0.033306
2.686863	2.290826	1.315889	0.019056	0.048139
3.81028	4.181822	-1.293735	0.018331	0.048139
6.041045	6.651598	-1.5307915	0.0063645	0.018727
10.270607	9.523886	1.677974	0.000137	0.001086
10.989847	10.4923	1.411811	0.000275	0.002333
10.9722035	9.1785565	3.57911	0.000025	0
9.48159	8.099966	2.605755	0.000156	0.0008225
3.359986	4.390828	-2.043217	0.01746	0.048139

4.625095	5.334441	-1.640476667	0.004435667	0.016884667
6.9960205	5.28227	3.280184	0.000201	0.0011665
4.590836	5.78719475	-2.3865185	0.0021695	0.0095105
3.719293	2.906206	1.756967	0.004583	0.022439
4.399711	3.296739	2.147967	0.001234	0.00587
6.753548	7.129113	-1.297347	0.011781	0.033306
10.952032	10.542824	1.327957	0.001676	0.009121
3.5047515	4.7499595	-2.4733095	0.0002395	0.0011665
9.367838	8.210072	2.231117	0.001261	0.00587
6.65373	5.432212	2.331921	0.000317	0.002333
5.431205	6.31358	-1.843408	0.015654	0.048139
6.57527625	8.15721975	-3.124953	0.0012255	0.005886
2.325084	3.318803	-1.991311	0.000276	0.002333
5.102352	6.054737	-1.935069	0.00112	0.00587
8.280517333	9.602032333	-2.518110667	4.46667E-05	0
6.116062	3.07411	12.68956367	0.002600667	0.009129333
4.006597	3.253038	1.685947	0.001674	0.009121
9.2867225	8.0867	2.2976585	0.0052855	0.022439
3.810227	4.235819	-1.343123	0.011874	0.033306
8.371629	7.403669	1.956073	0.00025	0.001645
9.884707	9.301685	1.497985	0.002779	0.015015
2.290952	5.938630333	-14.12422333	0.000024	0
8.778749667	7.44173	2.565734667	0.00019	0.001139667
4.761883	3.595211	2.298853667	0.005001667	0.015015333
4.427036	3.989698	1.354103	0.017263	0.048139
2.98198	3.5110015	-1.451255	0.0046235	0.018727
6.804395	7.510537333	-1.633176667	0.004543667	0.015015
4.396348667	6.572957667	-4.599637667	0.001179333	0.005105667
9.311950333	8.409955333	1.872723667	7.06667E-05	0.000362
5.190723	2.76219	5.383457	0.000586	0.003863
2.895229	3.463167	-1.482403	0.008691	0.022439
6.0553568	7.1252092	-2.1470252	0.00102	0.0038656
5.928302333	7.076271333	-2.234434333	0.012203	0.032153333
4.52688775	3.06329225	2.96369425	0.0026555	0.009629
5.013559	2.345838	6.354247	0.002021	0.009121

6.359782	6.018075	1.267255	0.020747	0.048139
7.1232165	5.619764	2.9083035	0.0001465	0.0008225
4.739242	3.8665	1.8376075	0.0052265	0.0241605
4.67794075	3.41501575	2.84940575	0.00081	0.004159
10.802615	9.790235	2.017236	0.000035	0
3.76771	4.312506	-1.458814	0.001143	0.00587
8.45676	7.648214	1.751446	0.003611	0.015015
10.193388	9.819926	1.295743	0.004679	0.018727
7.691205	6.689728	2.002049	0.000059	0
9.371103	8.453529	1.888936	0.000049	0
10.979518	9.578032	2.641735	0.000026	0
5.076228	3.976501	2.143142	0.000248	0.001645
4.0874	4.780736	-1.617018	0.003216	0.015015
6.975807	7.7687525	-1.739535	0.0041245	0.012386
6.510482	7.053907667	-1.459665333	0.013497333	0.037097667
5.8582725	5.3394195	1.433687	0.0043085	0.0174755
6.284715	5.849309	1.352292	0.017955	0.048139
7.063203	6.271249	1.731418	0.006675	0.022439
5.1640365	5.7540855	-1.50747	0.008102	0.0212135
3.142826	3.7091715	-1.481365	0.0066465	0.019588
9.930398	9.340535	1.505103	0.002586	0.015015
6.64657	7.392266	-1.676783	0.010581	0.033306
7.529495	7.20797	1.249651	0.018712	0.048139
7.366216	7.73345	-1.289878	0.020144	0.048139
3.974104	3.220583	1.685902	0.001304	0.00587
5.652877	4.1688135	3.0603445	0.000967	0.0045605
4.179985	3.245136	1.91169	0.007325	0.022439
5.487402	4.932424	1.469147	0.009076	0.033306
9.424678	8.46707	1.942087	0.000029	0
5.982102	5.264423	1.644534	0.006405	0.022439
4.988818	4.081256	1.875873	0.000269	0.001645
10.719192	9.454364	2.402986	0.000167	0.001645
4.7702618	5.4253958	-1.6114444	0.002802	0.0093486
5.042426	4.541112	1.415502	0.007385	0.022439
3.790908	4.49601	-1.63026	0.000481	0.002333

4.71819	5.310172	-1.507316	0.008596	0.022439
5.618202	4.540928	2.110045	0.00031	0.002333
3.478912	4.232802	-1.686334	0.001243	0.00587
5.540747667	6.399010333	-1.833016	0.002845333	0.01001
5.460019	6.079442	-1.53626	0.006014	0.022439
6.8162195	6.280581	1.4501425	0.006948	0.0278725
2.297692	2.921032	-1.540438	0.000769	0.003863
6.013465	6.530507	-1.431018	0.020984	0.048139
5.525215	6.212294	-1.615568	0.013832	0.0407225
3.834356333	4.283229667	-1.365655333	0.011415	0.035133
6.742691667	7.463988	-1.659683667	0.001109	0.004366333
7.292234	6.606293	1.608752	0.000295	0.002333
2.917645	3.828274	-1.879865	0.007441	0.022439
8.251427	7.7903245	1.3815665	0.0056225	0.0185845
2.52801	3.844115	-2.48993	0.000308	0.002333
6.087803	5.53466	1.467279	0.000799	0.00587
2.216151	2.882604	-1.587166	0.0105	0.033306
5.228543	6.639821	-2.689682	0.001031667	0.005201
5.798356667	6.519685	-1.650295333	0.002879	0.011393
2.016937	2.43564	-1.336725	0.017863	0.048139
6.0623885	6.511844	-1.365804	0.0189145	0.048139
3.069357	4.049862	-1.9739915	0.004902	0.018727
8.3223204	8.7859568	-1.3847798	0.0052782	0.0177766
2.854891	3.7032225	-1.820799	0.002354	0.008674
5.834165	3.924458	3.757327	0.004035	0.015015
2.20819	2.656944	-1.364861	0.003868	0.015015
4.289275	4.883237	-1.509387	0.008682	0.022439
3.230387	2.111322	2.172061	0.014224	0.048139
6.9727465	6.4524835	1.4419545	0.004679	0.019588
9.396703	9.957014	-1.474588	0.018854	0.048139
10.779119	10.390533	1.30911	0.018813	0.048139
5.457503	2.829727	6.180726	0.000044	0
6.576558667	7.255687333	-1.610687333	0.003686333	0.014346333
8.190907667	7.264462333	1.905355	0.000345	0.002427333
11.462987	10.856445	1.522606	0.000041	0

7.794544	6.675725	2.171692	0.000033	0
7.966479	6.394563	2.972993	0.00122	0.00587
6.362808	7.406973	-2.062173	0.000031	0
10.6854675	10.0829015	1.5189445	0.0005465	0.003863
10.574256	10.236116	1.264126	0.004405	0.022439
8.31928	7.025855	2.45419	0.000057	0
10.325955	9.958447	1.290123	0.000863	0.00587
6.1828045	8.3069455	-4.359877	0.0000385	0
7.657534	6.403803	2.384572	0.000299	0.002333
8.550123667	7.859475333	1.614697333	0.000704667	0.004876333
6.2485065	5.5289125	1.6525025	0.0048245	0.019588
8.268269667	7.513462333	1.700203333	0.000757333	0.003040333
5.828711	7.491674	-3.166661	0.005509	0.015015
4.482153	3.7755685	1.6417705	0.009451	0.025236
7.627111	8.163594	-1.450433	0.007362	0.022439
5.207609333	5.788778667	-1.502279333	0.012287	0.032153333
4.782498	5.272472	-1.404419	0.011821	0.033306
6.097577	6.855924	-1.697907	0.0017805	0.006492
5.943468429	6.716165	-1.719227429	0.001316	0.005326857
4.065996667	4.690664333	-1.548052667	0.001885333	0.007739333
5.839802	6.4454025	-1.5473765	0.007996	0.025236
3.471895	4.253569	-1.719123	0.003127	0.009121
4.046892	5.148382	-2.145762	0.00043	0.002333
5.49867325	6.1138985	-1.53660125	0.0019845	0.00727125
9.01059	8.528796	1.397544667	0.001290667	0.006284667
4.500682	4.968836	-1.383338	0.005292	0.015015
5.56427	6.204367	-1.558434	0.008517	0.022439
4.342214	3.435029	1.875384	0.017411	0.048139
3.9746665	3.4967415	1.4032535	0.011903	0.035289
9.806379	8.606893	2.296578	0.000028	0
3.7004635	4.368819	-1.5892895	0.00526	0.018727
2.020202	2.378107	-1.281564	0.018064	0.048139
5.0972395	3.045231	4.148508	0.0001035	0.000543
6.79478325	7.72294325	-1.90987725	0.00218525	0.00928
6.387940333	5.774521333	1.532996667	0.001812667	0.007739333

6.1107805	5.5245625	1.501411	0.004636	0.018727
2.492857	3.170808	-1.599866	0.001629	0.00587
2.215773	3.0358525	-1.776656	0.00121	0.006492
9.420944	8.4217805	1.999185	0.0000835	0.000543
5.827066	6.34784	-1.434725	0.021344	0.048139
4.738366	3.379567	2.564717	0.000378	0.002333
7.226331	6.615682	1.526947	0.003255	0.015015
4.113111	4.994596	-1.8442185	0.001672	0.0074955
4.939335	6.2166365	-2.4253705	0.0003705	0.0024745
8.258152	8.58594	-1.255088	0.018062	0.048139
3.288507	4.821172	-3.048794667	0.000069	0.000362
9.940813	9.462129	1.393472	0.001894	0.009121
7.0388135	6.142767	1.860989	0.001942	0.0075075
10.788959	10.449167	1.265573	0.011559	0.033306
4.743537	5.783826	-2.05664	0.00221	0.009121
12.437019	11.753029	1.606577	0.000089	0
10.709516	10.233323	1.391068	0.000984	0.00587
12.421572	12.052309	1.291693	0.002181	0.009121
7.095838667	5.198543667	3.728049667	9.23333E-05	0.000362
6.325563	4.660639	3.179183	0.002458	0.012068
6.36281	6.007391	1.279357	0.012742	0.048139
12.109497	12.384655	-1.210126	0.007275	0.022439
4.70137	5.242388333	-1.460678333	0.005091	0.015525
7.2150966	7.7295924	-1.4315086	0.0017054	0.0062184
7.737549	6.482853	2.386168	0.001979	0.009121
8.01737775	7.1421475	1.83979075	0.00656075	0.0188755
2.9815105	3.6431055	-1.5996925	0.004014	0.012042
4.514152	4.94967	-1.352396	0.015915	0.048139
2.219669	2.575832	-1.280017	0.017231	0.048139
10.704069	10.02207633	1.628423	0.005384667	0.019359333
3.060742	2.228795	1.780087	0.021044	0.048139
7.058781	5.81991	2.360137	0.000437	0.002333
5.505647	4.9891695	1.4304705	0.009367	0.033306
7.092584333	8.2885055	-2.295896333	0.000105333	0.000817167
4.333627	5.097543	-1.698094	0.009343	0.033306

2.029486	2.342343	-1.242165	0.003081	0.009121
11.049928	11.76827	-1.64529	0.015884	0.048139
4.886955	3.121	3.400991	0.000098	0
4.707608	4.23946	1.387514	0.0148875	0.048139
10.252076	9.630593	1.538499	0.002001	0.009439
10.080541	9.406554	1.595476	0.020685	0.048139
7.203744	6.664134	1.45358	0.00951	0.033306
8.5418085	7.8609645	1.6041215	0.016863	0.048139
4.333227	5.084902	-1.683746	0.005362	0.015015
4.540487	3.939443	1.516813	0.000596	0.003863
7.1948135	6.717035	1.392819	0.000067	0
7.599716	6.9294475	1.5990035	0.0084675	0.031577
4.790831	3.456063	2.52235	0.011928	0.033306
7.797681	6.902072	1.860395	0.00129	0.00587
3.625768	4.345018	-1.646326	0.004054	0.015015
5.524162	5.8858305	-1.2868405	0.005149	0.015015
5.196389	3.726212	2.7721825	0.0001485	0.0008225
4.858916	3.713621	2.211913	0.006686	0.022439
10.66512567	9.852031667	1.766827667	0.004210333	0.011102
5.129571	6.328246	-2.295286	0.001197	0.00587
6.412487	5.183873	2.349119	0.0007535	0.0048665
7.164369	6.415565	1.680399	0.000304	0.002333
5.531777	4.775817	1.688755	0.006137	0.022439
4.628042	3.656225333	2.002914667	0.007157333	0.019086667
5.1773585	4.5048895	1.597329	0.005294	0.0241605
3.996514	5.334609	-2.528172	0.000074	0
2.320187	2.9442965	-1.5414915	0.0092565	0.0278725
2.654535	3.474277	-1.7869495	0.000889	0.003863
6.587114	5.798427	1.727502	0.000476	0.003863
5.501375	4.705445	1.736196	0.013507	0.048139
2.614215	3.28578	-1.5928	0.001957	0.009121
5.385163333	5.903584667	-1.433011	0.00394	0.013050333
7.488236	4.899908	6.014013	0.000028	0
7.043347	4.659195	5.22037	0.000581	0.003863
7.989178	8.638363	-1.568282	0.017801	0.048139

4.555698	5.5074	-1.934154	0.000344	0.002333
5.6020265	6.260974	-1.5832825	0.0008345	0.0041015
7.134929	7.707629	-1.487304	0.000097	0.001086
4.613383667	4.161082667	1.376256	0.011790667	0.032153333
9.39602225	8.18165725	2.327165	0.000089	0.000543
6.8749355	6.0494775	1.7746155	0.0146705	0.048139
7.50917675	8.15900925	-1.57148	0.00058225	0.0027335
4.593102	6.445743	-3.611606	0.003257	0.015015
4.904381	5.993775	-2.127847	0.002691	0.009121
7.775795	8.379036	-1.519125	0.00045	0.002333
8.148414	7.313038	1.793998333	0.005283333	0.022981667
11.897073	11.600949	1.227841	0.018566	0.048139
8.5983445	7.873236	1.656988	0.0016975	0.009439
7.181318	7.706523	-1.4404055	0.004658	0.012042
4.2677975	4.9013005	-1.551542	0.009882	0.0278725
4.324620667	4.976919333	-1.590607	0.007656333	0.022127
9.736943	10.112625	-1.297453	0.007471	0.022439
4.1247805	4.7600925	-1.553591	0.013951	0.0407225
3.0462945	3.8068655	-1.694974	0.0056015	0.0212135
5.620284	5.0317125	1.507646	0.0046935	0.018727
2.726090333	4.109889	-2.668744667	0.000938333	0.00418
6.8995612	7.5927098	-1.6655846	0.0015084	0.0052938
5.265647	4.597451	1.589085	0.000668	0.003863
4.014168	4.7335485	-1.667702	0.0002915	0.0011665
5.78845	3.640462	4.432094	0.000056	0
10.0408865	7.8333595	4.6547285	0.0000435	0
6.502213	7.140576	-1.556562	0.01208	0.033306
6.174436	5.518932	1.575166	0.009902	0.033306
6.180526	5.507252	1.594688	0.001733	0.009121
5.450386	6.03551	-1.500384	0.001176	0.00587
3.953702667	2.793919667	2.349840667	0.000708333	0.003588667
4.906943333	3.635550667	2.546086667	0.001270667	0.006961667
5.24482525	6.61447375	-2.59292375	0.00152325	0.00643925
6.598694	6.880707	-1.21589	0.019338	0.048139
5.7028675	6.5488415	-1.7998025	0.005125	0.01578

3.8194	4.596255	-1.7134055	0.003242	0.0117625
5.2532065	6.4702085	-2.3961005	0.0009945	0.0048665
7.450609	8.90854	-2.747792	0.0001925	0.0013655
8.22358	8.735686	-1.426131	0.006663	0.022439
6.1888055	3.711378	5.576243	0.0000875	0
5.330672	4.611613	1.646109	0.016564	0.048139
4.9807135	5.7853505	-1.7504155	0.000278	0.001989
6.822576	5.629944	2.285693	0.007623	0.033306
3.158854	2.831685	1.254549	0.002668	0.015015
2.62714	3.115017	-1.40238	0.003492	0.015015
5.668615	2.824268	7.181808	0.000118	0.001086
2.756689	3.323303	-1.481043	0.014167	0.033306
2.602783	3.013553	-1.329395	0.012087	0.033306
10.377833	9.914026	1.379177	0.010667	0.033306
2.154238	2.890823	-1.666227	0.003193	0.015015
4.0637825	4.929198	-1.8557905	0.0022635	0.008674
5.146274	5.75118	-1.52088	0.001786	0.009121
3.207507	3.746733	-1.455163	0.008461	0.0241605
7.422454	9.320486	-3.727042	0.000102	0.001086
11.264362	10.976823	1.220557	0.012135	0.033306
5.253499	4.868064	1.306253	0.014499	0.048139
11.079072	10.759639	1.24784	0.020172	0.048139
9.686959	7.785289	3.736455	0.000031	0
8.073372	8.54165	-1.383457	0.013464	0.033306
6.059191	5.373928667	1.618335333	0.003182	0.012476667
6.591332	5.556018667	2.123068	0.001589667	0.006961667
7.395676	6.78418	1.527842	0.00169	0.009121
4.766806	3.990567	1.7857065	0.0022775	0.0104425
4.406304	2.795999	3.053164	0.000907	0.00587
7.981565	7.422198	1.473622	0.011644	0.033306
6.578112	5.976142	1.517787	0.001017	0.00587
10.614525	10.310459	1.23462	0.020631	0.048139
8.576681	7.90478	1.59317	0.011195	0.033306
4.238537	3.511842	1.654844	0.0141	0.048139
3.1834355	3.8298465	-1.566783	0.0097645	0.0270045

7.755189	7.219246333	1.450292333	0.005012333	0.019359333
6.8691695	6.368291	1.416294	0.0001335	0.0008225
6.283529	6.637105	-1.277724	0.016403	0.048139
6.837646	7.372669	-1.448965	0.019641	0.048139
7.304943	6.8532925	1.3715665	0.0008845	0.005383
5.250911	5.710809	-1.375445	0.003989	0.015015
3.853249	4.595769	-1.680005333	0.002075333	0.010002
6.148844	5.883217	1.202159	0.005218	0.022439
6.044617	4.524621	2.867902	0.000847	0.00587
10.922675	9.998103	1.89812	0.005628	0.022439
7.2348698	7.8082206	-1.4950592	0.003145	0.0116058
7.63058	6.159975	2.771382	0.000525	0.003863
8.3785935	7.3438495	2.050395	0.0016695	0.0075075
8.158274	8.985165	-1.773859	0.000036	0
5.996015	5.5527705	1.3599585	0.003066	0.0141545
3.5361815	4.374742	-1.816624	0.0045855	0.0141545
3.5266725	4.2933195	-1.71777475	0.01003675	0.027008
8.033029	7.343521	1.612733	0.000201	0.001645
3.253032	2.639384	1.530123	0.003355	0.015015
5.1391555	3.76904875	2.62177025	0.0019915	0.00757
3.428474	7.751925	-20.021129	0.000024	0
8.013626	7.394339	1.536116	0.010983	0.033306
8.453416	7.065246	2.617464	0.000227	0.001645
6.724539	7.219936	-1.409709	0.005464	0.015015
4.8453335	5.517533	-1.6033835	0.001021	0.0045605
3.746984	5.01831	-2.413833	0.003186	0.015015
5.156238	6.147773	-1.9883	0.00869	0.022439
5.60122	5.211165	1.310443	0.017598	0.048139
10.681711	10.315392	1.28906	0.005563	0.022439
2.802473	3.863104	-2.085843	0.000454	0.002333
2.409105	3.169124	-1.693513	0.00809	0.022439
10.85992	10.4280095	1.3490725	0.0000275	0
7.713943667	6.580064667	2.195015	0.001634333	0.008918333
5.140053	4.863634	1.211185	0.005382	0.022439
9.121762833	8.187036333	1.917084167	4.71667E-05	0.000181

8.344461	6.798798333	2.977720333	0.001699	0.007479667
2.937849	3.937546	-1.999579	0.000554	0.003863
7.635568	4.976766	6.315083	0.000026	0
7.305896	6.5039575	1.7451515	0.0003825	0.0019315
3.213207	4.06377	-1.803204	0.004417	0.015015
3.8639885	4.3962715	-1.4489695	0.000304	0.001989
4.477761	5.2761925	-1.7491795	0.002346	0.0075075
7.1477665	7.851458	-1.6297745	0.0006485	0.002935
10.776129	10.45404	1.25014	0.02067	0.048139
6.186293	5.23634	1.93181	0.013506	0.048139
12.404373	11.451002	1.936392	0.000027	0
9.343582	6.4246205	7.5652545	0.000058	0
8.357829	7.951347667	1.325869333	0.007079333	0.026566333
6.534036	5.103825	2.694862	0.000705	0.003863
4.852501	2.3129525	5.8776185	0.0019455	0.009121
3.600975	4.456404333	-1.835703	0.001013667	0.003402333
2.294809	2.688128	-1.313412	0.019204	0.048139
4.361126	4.805171	-1.360414	0.000765	0.003863
9.030713	9.513761	-1.397694	0.006014	0.022439
5.9913776	6.7208972	-1.6705164	0.0061096	0.0183578
9.452344333	10.08561433	-1.559201333	0.000182333	0.000777667
8.0982895	7.305102	1.734371	0.0014415	0.006492
7.828392333	7.065204667	1.699221	0.006481333	0.018780667
5.2811925	5.661351	-1.301771	0.007231	0.0185845
7.153747	6.697289	1.372169	0.008797	0.033306
6.426117	5.645502	1.717864	0.008163	0.033306
3.265568	3.979262	-1.639999	0.002113	0.009121
8.8677015	9.4814395	-1.5314395	0.002384	0.009439
6.776556	5.569448	2.308743	0.000074	0
7.653077333	8.077909333	-1.342769	0.012590333	0.032153333
5.209184	3.969683333	2.421580167	0.001109667	0.005766
4.301243	4.794924	-1.408034	0.016385	0.048139
6.431935	7.124517	-1.616173	0.010736	0.033306
5.257404	4.933101	1.252059	0.004718	0.022439
4.7112635	5.6448245	-1.9417625	0.001425	0.0045605

4.955067	2.5976	5.1247	0.003067	0.015015
7.995275	7.451398	1.457885	0.000094	0
3.6731695	4.702926	-2.068151	0.0023795	0.009439
4.747537	3.641276	2.15287	0.001941	0.009121
7.8350515	7.4355195	1.319102	0.003037	0.0112195
6.370308	6.801892	-1.348714	0.011123	0.033306
2.909232	3.407512	-1.412529	0.010953	0.033306
4.584351	5.228728	-1.563065	0.01755	0.048139
2.302693	3.047153	-1.675347	0.00178	0.009121
2.995521	3.7433885	-1.7036055	0.002235	0.0074955
2.019642	2.431927	-1.330792	0.000424	0.002333
4.175933	5.342595	-2.244917	0.001116	0.00587
3.885531	4.551281	-1.586394	0.009012	0.0278725
2.476713	2.964179	-1.40198	0.015402	0.048139
3.788312	4.583574	-1.7408485	0.0082695	0.0241605
5.4201705	6.416366	-1.994776	0.0002665	0.0011665
4.119905	4.813881	-1.617736	0.003025	0.009121
4.776378	4.275657	1.41492	0.001954	0.009121
2.569772	3.590932	-2.02955	0.001487	0.00587
5.421013667	6.481393667	-2.134163667	0.001072333	0.005201
2.649193	2	1.568291	0.011692	0.033306
3.26922	4.023962	-1.68733	0.008183	0.022439
3.0273035	3.9104425	-1.8446705	0.0005655	0.003098
6.33797	5.387747	1.932171	0.002413	0.009121
4.602778	5.031083	-1.345652	0.012535	0.033306
2.787349	3.45605	-1.589642	0.018496	0.048139
2.262811	2.957238	-1.618241	0.00069	0.003863
2.061435	2.415827	-1.278446	0.015834	0.048139
5.4831675	6.249728	-1.7077445	0.004666	0.013151
5.538122	6.133551	-1.510922	0.015296	0.048139
2.584345	3.069548	-1.399783	0.012879	0.033306
5.23705	5.824829	-1.502932	0.009049	0.022439
6.471864	6.097503	1.296265	0.006283	0.022439
3.96635	4.587601	-1.538208	0.000461	0.002333
2.895406	3.576054	-1.60286	0.000714	0.003863

7.371868	7.937666	-1.4802695	0.0011315	0.005383
3.201984	3.590411	-1.308966	0.006277	0.022439
2.141177	2.792461	-1.570565	0.009083	0.033306
2.782083	5.145718	-5.146654	0.000041	0
4.1805915	4.6832775	-1.4220765	0.010219	0.0270045
