# Supplementary material for: Long noncoding RNA GSEC promotes neutrophil inflammatory activation by supporting PFKFB3-involved glycolytic metabolism in sepsis
Source: Cell Death Dis. 2021 Dec 14;12(12):1157. doi: 10.1038/s41419-021-04428-7 (PMC8671582; doi:10.1038/s41419-021-04428-7)
Supplement: Supplementary file 5 — Supplementary Table 3 [file 41419_2021_4428_MOESM5_ESM.pdf]

Supplementary Table 3. 11 co-expressed lncRNAs.

Gene Symbol	Biotype	style	Degree
NONHSAT160878.1	lncRNA	up	47
XR_926068.1	lncRNA	up	31
NONHSAT010176.2	lncRNA	up	30
XR_941546.1	lncRNA	up	24
ENST00000629441.1	lncRNA	up	10
NONHSAT081776.2	lncRNA	up	6
NONHSAT181667.1	lncRNA	up	5
NONHSAT131038.2	lncRNA	down	7
ENST00000608064.1	lncRNA	down	4
NONHSAT009146.2	lncRNA	down	1
NR_003945.1	lncRNA	down	1
