# Supplementary material for: Long noncoding RNA GSEC promotes neutrophil inflammatory activation by supporting PFKFB3-involved glycolytic metabolism in sepsis
Source: Cell Death Dis. 2021 Dec 14;12(12):1157. doi: 10.1038/s41419-021-04428-7 (PMC8671582; doi:10.1038/s41419-021-04428-7)
Supplement: Supplementary file 6 — Supplementary Table 4 [file 41419_2021_4428_MOESM6_ESM.pdf]

Supplementary Table 4. 105 co-expressed mRNAs.

Gene Symbol	Biotype	Style	Degree
ACER3	mRNA	up	10
ACVR1B	mRNA	up	6
ADAM9	mRNA	up	5
ADGRG3	mRNA	up	2
AGFG1	mRNA	up	15
AKIRIN2	mRNA	up	1
ALPL	mRNA	up	3
ANXA3	mRNA	up	8
ASPH	mRNA	up	5
ATP11B	mRNA	up	11
ATP6V1C1	mRNA	up	6
ATP9A	mRNA	up	2
BCLAF1	mRNA	down	2
BUB3	mRNA	down	1
CAB39	mRNA	up	1
CARD6	mRNA	up	22
CCDC71L	mRNA	up	1
CD177	mRNA	up	13
CDC42EP3	mRNA	up	11
CDK5RAP2	mRNA	up	3
CR1	mRNA	up	6
CST7	mRNA	up	1
CYLD	mRNA	down	2
CYP1B1	mRNA	up	2
CYSTM1	mRNA	up	6
DACH1	mRNA	up	6
DDAH2	mRNA	up	7
DOK3	mRNA	up	1
DYSF	mRNA	up	5
ECHDC3	mRNA	up	2
EMILIN2	mRNA	up	9
ETS2	mRNA	up	9
EXOC6	mRNA	up	9
EXOSC4	mRNA	up	4
F5	mRNA	up	1
FCAR	mRNA	up	1
FCER1G	mRNA	up	19
FKBP5	mRNA	up	5
FLOT2	mRNA	up	2
GALNT14	mRNA	up	3
GAS7	mRNA	up	9
GPR84	mRNA	up	10
GRB10	mRNA	up	7
GYG1	mRNA	up	30
IER3	mRNA	up	1
IL18R1	mRNA	up	4
IRAK3	mRNA	up	19
KIAA0930	mRNA	up	4
KIF1B	mRNA	up	10
LILRA5	mRNA	up	1
LOC100507507	mRNA	up	2
LRG1	mRNA	up	1
LRPAP1	mRNA	up	1
MAP2K6	mRNA	up	6
MAPK14	mRNA	up	25
MCEMP1	mRNA	up	24

METTL9	mRNA	up	3
NAIP	mRNA	up	8
NLRC4	mRNA	up	13
OPLAH	mRNA	up	1
P2RY10	mRNA	down	2
PDCD4	mRNA	down	3
PDGFC	mRNA	up	1
PECR	mRNA	up	1
PFKFB3	mRNA	up	3
PGD	mRNA	up	2
PGK1	mRNA	up	2
PGS1	mRNA	up	11
POR	mRNA	up	5
PPP2R5C	mRNA	down	1
PPTC7	mRNA	down	1
PRKAA1	mRNA	up	1
PUM2	mRNA	down	1
PYGL	mRNA	up	13
RAB20	mRNA	up	2
RAB27A	mRNA	up	2
RAB32	mRNA	up	7
RALGAPA2	mRNA	up	1
RANBP9	mRNA	up	1
RBMS1	mRNA	up	4
RNASEH2B	mRNA	down	1
S100A9	mRNA	up	1
SDHC	mRNA	up	1
SERPINB1	mRNA	up	5
SET	mRNA	down	1
SH3GLB1	mRNA	up	6
SIPA1L2	mRNA	up	1
SLC22A4	mRNA	up	2
SLC25A40	mRNA	up	1
SLC26A8	mRNA	up	2
SRPK1	mRNA	up	1
STOM	mRNA	up	5
TBC1D8	mRNA	up	10
TDRD9	mRNA	up	12
TLR5	mRNA	up	12
TMCO3	mRNA	up	1
TSPO	mRNA	up	7
UPP1	mRNA	up	21
USB1	mRNA	up	1
VNN1	mRNA	up	2
ZCCHC7	mRNA	down	1
ZDHHC19	mRNA	up	1
ZDHHC3	mRNA	up	4
ZEB1	mRNA	down	1
ZNF438	mRNA	up	7
