# Supplementary material for: Long noncoding RNA GSEC promotes neutrophil inflammatory activation by supporting PFKFB3-involved glycolytic metabolism in sepsis
Source: Cell Death Dis. 2021 Dec 14;12(12):1157. doi: 10.1038/s41419-021-04428-7 (PMC8671582; doi:10.1038/s41419-021-04428-7)
Supplement: Supplementary file 7 — Supplementary Table 5 [file 41419_2021_4428_MOESM7_ESM.pdf]

Supplementary Table 5. 35 upregulated biological processes of the 105 co-expressed mRNAs.

go_id	go_name	Style	go_diffgene_count	pvalue	FDR	-lg(P)
GO:0045087	innate immune response	Up	11	4.7656E-09	2.4829E-06	8.321884792
GO:0006006	glucose metabolic process	Up	6	6.7964E-08	1.7705E-05	7.167718812
GO:0042742	defense response to bacterium	Up	5	2.403E-06	0.00034473	5.6192427
GO:0055114	oxidation-reduction process	Up	5	2.6467E-06	0.00034473	5.577296208
GO:0019395	fatty acid oxidation	Up	3	5.2229E-06	0.00054423	5.282086655
GO:0002755	MyD88-dependent toll-like receptor signaling pathway	Up	4	3.3169E-05	0.0027073	4.479267034
GO:0044281	small molecule metabolic process	Up	11	3.6374E-05	0.0027073	4.43920398
GO:0043066	negative regulation of apoptotic process	Up	7	4.508E-05	0.00270972	4.34601368
GO:0005975	carbohydrate metabolic process	Up	6	5.5643E-05	0.00270972	4.254586707
GO:0009437	carnitine metabolic process	Up	2	5.7211E-05	0.00270972	4.242519902
GO:0046545	development of primary female sexual characteristics	Up	2	5.7211E-05	0.00270972	4.242519902
GO:0045542	positive regulation of cholesterol biosynthetic process	Up	2	9.5255E-05	0.00413566	4.02111151
GO:0032496	response to lipopolysaccharide	Up	4	0.00012793	0.00512687	3.893042455
GO:0010042	response to manganese ion	Up	2	0.00014274	0.00531189	3.845460559
GO:0007050	cell cycle arrest	Up	4	0.00020115	0.00698662	3.696479546
GO:0006915	apoptotic process	Up	7	0.00030304	0.00986789	3.51849337
GO:0002544	chronic inflammatory response	Up	2	0.00034153	0.01022827	3.466569936
GO:0008286	insulin receptor signaling pathway	Up	4	0.00035338	0.01022827	3.45176303
GO:0006954	inflammatory response	Up	5	0.00041016	0.01124688	3.38705193
GO:0034166	toll-like receptor 10 signaling pathway	Up	3	0.00059177	0.01468161	3.227844676
GO:0034146	toll-like receptor 5 signaling pathway	Up	3	0.00059177	0.01468161	3.227844676
GO:0007165	signal transduction	Up	8	0.00084029	0.01864642	3.075569325
GO:0042149	cellular response to glucose starvation	Up	2	0.00085895	0.01864642	3.066031068
GO:0045332	phospholipid translocation	Up	2	0.00085895	0.01864642	3.066031068
GO:0043065	positive regulation of apoptotic process	Up	4	0.0010576	0.02204035	2.975679321
GO:0005980	glycogen catabolic process	Up	2	0.00143833	0.02882201	2.842140138
GO:0045648	positive regulation of erythrocyte differentiation	Up	2	0.00160592	0.03098838	2.794275109
GO:0000187	activation of MAPK activity	Up	3	0.00181311	0.03373673	2.741576657
GO:0008284	positive regulation of cell proliferation	Up	5	0.0018878	0.03391527	2.724044469
GO:0051092	positive regulation of NF-kappaB transcription factor activity	Up	3	0.0027071	0.04549673	2.56749588
GO:0002224	toll-like receptor signaling pathway	Up	3	0.0027071	0.04549673	2.56749588
GO:0048010	vascular endothelial growth factor receptor signaling pathway	Up	2	0.00280036	0.04559329	2.552786806
GO:0051149	positive regulation of muscle cell differentiation	Up	2	0.00326979	0.0489271	2.485479486
GO:2000379	positive regulation of reactive oxygen species metabolic process	Up	2	0.00326979	0.0489271	2.485479486
GO:0007596	blood coagulation	Up	5	0.00328685	0.0489271	2.483220186
