# Supplementary material for: Long noncoding RNA GSEC promotes neutrophil inflammatory activation by supporting PFKFB3-involved glycolytic metabolism in sepsis
Source: Cell Death Dis. 2021 Dec 14;12(12):1157. doi: 10.1038/s41419-021-04428-7 (PMC8671582; doi:10.1038/s41419-021-04428-7)
Supplement: Supplementary file 8 — Supplementary Table 6 [file 41419_2021_4428_MOESM8_ESM.pdf]

Supplementary Table 6. 19 downregulated biological processes of the 105 co-expressed mRNAs.

go_id	go_name	Style	go_diffgene_count	pvalue	FDR	-lg(P)
GO:0045892	negative regulation of transcription, DNA-dependent	Down	4	5.7459E-06	0.00045393	5.24063849
GO:2000144	positive regulation of DNA-dependent transcription, initiation	Down	1	0.00080201	0.02111947	3.09582292
GO:0010464	regulation of mesenchymal cell proliferation	Down	1	0.00080201	0.02111947	3.09582292
GO:0006917	induction of apoptosis	Down	2	0.00237972	0.03166174	2.62347447
GO:0071173	spindle assembly checkpoint	Down	1	0.00240557	0.03166174	2.61878149
GO:0051983	regulation of chromosome segregation	Down	1	0.00400855	0.03166174	2.39701256
GO:0043620	regulation of DNA-dependent transcription in response to stress	Down	1	0.00400855	0.03166174	2.39701256
GO:0033081	regulation of T cell differentiation in thymus	Down	1	0.00400855	0.03166174	2.39701256
GO:0051150	regulation of smooth muscle cell differentiation	Down	1	0.00480982	0.03166174	2.31787122
GO:0016070	RNA metabolic process	Down	2	0.00485924	0.03166174	2.31343137
GO:0008608	attachment of spindle microtubules to kinetochore	Down	1	0.00561094	0.03166174	2.25096434
GO:0030857	negative regulation of epithelial cell differentiation	Down	1	0.00561094	0.03166174	2.25096434
GO:0042347	negative regulation of NF-kappaB import into nucleus	Down	1	0.00561094	0.03166174	2.25096434
GO:2001022	positive regulation of response to DNA damage stimulus	Down	1	0.00561094	0.03166174	2.25096434
GO:0070507	regulation of microtubule cytoskeleton organization	Down	1	0.00641191	0.03351803	2.1930123
GO:0035067	negative regulation of histone acetylation	Down	1	0.00721274	0.03351803	2.14189969
GO:0034063	stress granule assembly	Down	1	0.00721274	0.03351803	2.14189969
GO:0008285	negative regulation of cell proliferation	Down	2	0.00905348	0.0397347	2.04318467
GO:0043508	negative regulation of JUN kinase activity	Down	1	0.00961434	0.0399754	2.01708067
