# Supplementary material for: Long noncoding RNA GSEC promotes neutrophil inflammatory activation by supporting PFKFB3-involved glycolytic metabolism in sepsis
Source: Cell Death Dis. 2021 Dec 14;12(12):1157. doi: 10.1038/s41419-021-04428-7 (PMC8671582; doi:10.1038/s41419-021-04428-7)
Supplement: Supplementary file 9 — Supplementary Table 7 [file 41419_2021_4428_MOESM9_ESM.pdf]

Supplementary Table 7. 11 immune response related upregulated biological processes.

go_id	go_number	go_name	enrichment	pvalue	FDR	gene_id	gene_name	gene_number
GO:0000187	1	activation of MAPK activity	20.32258065	0.001813107	0.033736734	5608	MAP2K6	8
GO:0000187	1	activation of MAPK activity	20.32258065	0.001813107	0.033736734	1432	MAPK14	9
GO:0000187	1	activation of MAPK activity	20.32258065	0.001813107	0.033736734	5562	PRKAA1	12
GO:0002224	2	toll-like receptor signaling pathway	17.71234093	0.002707099	0.045496726	5608	MAP2K6	8
GO:0002224	2	toll-like receptor signaling pathway	17.71234093	0.002707099	0.045496726	1432	MAPK14	9
GO:0002224	2	toll-like receptor signaling pathway	17.71234093	0.002707099	0.045496726	7100	TLR5	15
GO:0002544	3	chronic inflammatory response	143.0107527	0.000341531	0.01022827	6280	S100A9	13
GO:0002544	3	chronic inflammatory response	143.0107527	0.000341531	0.01022827	8876	VNN1	16
GO:0002755	4	MyD88-dependent toll-like receptor signaling pathway	31.78016726	3.3169E-05	0.002707296	11213	IRAK3	6
GO:0002755	4	MyD88-dependent toll-like receptor signaling pathway	31.78016726	3.3169E-05	0.002707296	5608	MAP2K6	8
GO:0002755	4	MyD88-dependent toll-like receptor signaling pathway	31.78016726	3.3169E-05	0.002707296	1432	MAPK14	9
GO:0002755	4	MyD88-dependent toll-like receptor signaling pathway	31.78016726	3.3169E-05	0.002707296	7100	TLR5	15
GO:0006954	5	inflammatory response	10.90759978	0.000410155	0.011246883	4671	NAIP	10
GO:0006954	5	inflammatory response	10.90759978	0.000410155	0.011246883	58484	NLRC4	11
GO:0006954	5	inflammatory response	10.90759978	0.000410155	0.011246883	6280	S100A9	13
GO:0006954	5	inflammatory response	10.90759978	0.000410155	0.011246883	7100	TLR5	15
GO:0006954	5	inflammatory response	10.90759978	0.000410155	0.011246883	8876	VNN1	16
GO:0032496	6	response to lipopolysaccharide	22.58064516	0.000127926	0.005126865	55122	AKIRIN2	1
GO:0032496	6	response to lipopolysaccharide	22.58064516	0.000127926	0.005126865	249	ALPL	2
GO:0032496	6	response to lipopolysaccharide	22.58064516	0.000127926	0.005126865	11213	IRAK3	6
GO:0032496	6	response to lipopolysaccharide	22.58064516	0.000127926	0.005126865	6280	S100A9	13
GO:0034146	7	toll-like receptor 5 signaling pathway	29.70223325	0.000591773	0.014681612	5608	MAP2K6	8
GO:0034146	7	toll-like receptor 5 signaling pathway	29.70223325	0.000591773	0.014681612	1432	MAPK14	9
GO:0034146	7	toll-like receptor 5 signaling pathway	29.70223325	0.000591773	0.014681612	7100	TLR5	15
GO:0034166	8	toll-like receptor 10 signaling pathway	29.70223325	0.000591773	0.014681612	5608	MAP2K6	8
GO:0034166	8	toll-like receptor 10 signaling pathway	29.70223325	0.000591773	0.014681612	1432	MAPK14	9
GO:0034166	8	toll-like receptor 10 signaling pathway	29.70223325	0.000591773	0.014681612	7100	TLR5	15
GO:0042742	9	defense response to bacterium	31.54648956	2.40302E-06	0.000344732	306	ANXA3	3
GO:0042742	9	defense response to bacterium	31.54648956	2.40302E-06	0.000344732	2207	FCER1G	5
GO:0042742	9	defense response to bacterium	31.54648956	2.40302E-06	0.000344732	58484	NLRC4	11
GO:0042742	9	defense response to bacterium	31.54648956	2.40302E-06	0.000344732	6280	S100A9	13
GO:0042742	9	defense response to bacterium	31.54648956	2.40302E-06	0.000344732	7100	TLR5	15
GO:0045087	10	innate immune response	12.77803657	4.76557E-09	2.48286E-06	55122	AKIRIN2	1
GO:0045087	10	innate immune response	12.77803657	4.76557E-09	2.48286E-06	1378	CR1	4
GO:0045087	10	innate immune response	12.77803657	4.76557E-09	2.48286E-06	353514	LILRA5	7
GO:0045087	10	innate immune response	12.77803657	4.76557E-09	2.48286E-06	5608	MAP2K6	8

GO:0045087	10	innate immune response	12.77803657	4.76557E-09	2.48286E-06	1432	MAPK14	9
GO:0045087	10	innate immune response	12.77803657	4.76557E-09	2.48286E-06	4671	NAIP	10
GO:0045087	10	innate immune response	12.77803657	4.76557E-09	2.48286E-06	58484	NLRC4	11
GO:0045087	10	innate immune response	12.77803657	4.76557E-09	2.48286E-06	6280	S100A9	13
GO:0045087	10	innate immune response	12.77803657	4.76557E-09	2.48286E-06	6732	SRPK1	14
GO:0045087	10	innate immune response	12.77803657	4.76557E-09	2.48286E-06	7100	TLR5	15
GO:0045087	10	innate immune response	12.77803657	4.76557E-09	2.48286E-06	8876	VNN1	16
GO:0051092	11	positive regulation of NF-kappaB transcription factor ac	17.71234093	0.002707099	0.045496726	11213	IRAK3	6
GO:0051092	11	positive regulation of NF-kappaB transcription factor ac	17.71234093	0.002707099	0.045496726	58484	NLRC4	11
GO:0051092	11	positive regulation of NF-kappaB transcription factor ac	17.71234093	0.002707099	0.045496726	6280	S100A9	13
