# Supplementary material for: Long noncoding RNA GSEC promotes neutrophil inflammatory activation by supporting PFKFB3-involved glycolytic metabolism in sepsis
Source: Cell Death Dis. 2021 Dec 14;12(12):1157. doi: 10.1038/s41419-021-04428-7 (PMC8671582; doi:10.1038/s41419-021-04428-7)
Supplement: Supplementary file 10 — Supplementary Table 8 [file 41419_2021_4428_MOESM10_ESM.pdf]

Supplementary Table 8. 11 metabolic process related upregulated biological processes.

go_id	go_number	go_name	enrichment	pvalue	FDR	gene_id	gene_name	gene_number
GO:0005975	1	carbohydrate metabolic process	11.66552968	5.56434E-05	0.002709724	79623	GALNT14	7
GO:0005975	1	carbohydrate metabolic process	11.66552968	5.56434E-05	0.002709724	2992	GYG1	9
GO:0005975	1	carbohydrate metabolic process	11.66552968	5.56434E-05	0.002709724	5209	PFKFB3	12
GO:0005975	1	carbohydrate metabolic process	11.66552968	5.56434E-05	0.002709724	5226	PGD	13
GO:0005975	1	carbohydrate metabolic process	11.66552968	5.56434E-05	0.002709724	5230	PGK1	14
GO:0005975	1	carbohydrate metabolic process	11.66552968	5.56434E-05	0.002709724	5836	PYGL	18
GO:0009437	2	carnitine metabolic process	321.7741935	5.72111E-05	0.002709724	5447	POR	16
GO:0009437	2	carnitine metabolic process	321.7741935	5.72111E-05	0.002709724	6583	SLC22A4	20
GO:0042149	3	cellular response to glucose starvation	91.93548387	0.000858952	0.018646418	5562	PRKAA1	17
GO:0042149	3	cellular response to glucose starvation	91.93548387	0.000858952	0.018646418	7378	UPP1	22
GO:0019395	4	fatty acid oxidation	137.9032258	5.22292E-06	0.000544228	1432	MAPK14	10
GO:0019395	4	fatty acid oxidation	137.9032258	5.22292E-06	0.000544228	5447	POR	16
GO:0019395	4	fatty acid oxidation	137.9032258	5.22292E-06	0.000544228	5562	PRKAA1	17
GO:0006006	5	glucose metabolic process	36.77419355	6.79644E-08	1.77047E-05	2992	GYG1	9
GO:0006006	5	glucose metabolic process	36.77419355	6.79644E-08	1.77047E-05	1432	MAPK14	10
GO:0006006	5	**glucose metabolic process**	**36.77419355**	**6.79644E-08**	**1.77047E-05**	**5209**	**PFKFB3**	**12**
GO:0006006	5	**glucose metabolic process**	**36.77419355**	**6.79644E-08**	**1.77047E-05**	**5230**	**PGK1**	**14**
GO:0006006	5	glucose metabolic process	36.77419355	6.79644E-08	1.77047E-05	5562	PRKAA1	17
GO:0006006	5	glucose metabolic process	36.77419355	6.79644E-08	1.77047E-05	5836	PYGL	18
GO:0005980	6	glycogen catabolic process	71.50537634	0.001438334	0.028822008	2992	GYG1	9
GO:0005980	6	glycogen catabolic process	71.50537634	0.001438334	0.028822008	5836	PYGL	18
GO:0008286	7	insulin receptor signaling pathway	17.39319965	0.000353376	0.01022827	528	ATP6V1C1	3
GO:0008286	7	insulin receptor signaling pathway	17.39319965	0.000353376	0.01022827	51719	CAB39	5
GO:0008286	7	insulin receptor signaling pathway	17.39319965	0.000353376	0.01022827	2887	GRB10	8
GO:0008286	7	insulin receptor signaling pathway	17.39319965	0.000353376	0.01022827	5562	PRKAA1	17
GO:0045332	8	phospholipid translocation	91.93548387	0.000858952	0.018646418	23200	ATP11B	2
GO:0045332	8	phospholipid translocation	91.93548387	0.000858952	0.018646418	10079	ATP9A	4
GO:0045542	9	positive regulation of cholesterol biosynthetic process	257.4193548	9.52552E-05	0.004135661	5447	POR	16
GO:0045542	9	positive regulation of cholesterol biosynthetic process	257.4193548	9.52552E-05	0.004135661	5562	PRKAA1	17
GO:2000379	10	positive regulation of reactive oxygen species metabolic process	47.6702509	0.003269795	0.048927102	1432	MAPK14	10
GO:2000379	10	positive regulation of reactive oxygen species metabolic process	47.6702509	0.003269795	0.048927102	706	TSPO	22
GO:0044281	11	small molecule metabolic process	5.193714056	3.63744E-05	0.002707296	55331	ACER3	1
GO:0044281	11	small molecule metabolic process	5.193714056	3.63744E-05	0.002707296	1545	CYP1B1	6
GO:0044281	11	small molecule metabolic process	5.193714056	3.63744E-05	0.002707296	2992	GYG1	9
GO:0044281	11	small molecule metabolic process	5.193714056	3.63744E-05	0.002707296	26873	OPLAH	11
GO:0044281	11	small molecule metabolic process	5.193714056	3.63744E-05	0.002707296	5209	PFKFB3	12

GO:0044281	11	small molecule metabolic process	5.193714056	3.63744E-05	0.002707296	5226	PGD	13
GO:0044281	11	small molecule metabolic process	5.193714056	3.63744E-05	0.002707296	5230	PGK1	14
GO:0044281	11	small molecule metabolic process	5.193714056	3.63744E-05	0.002707296	9489	PGS1	15
GO:0044281	11	small molecule metabolic process	5.193714056	3.63744E-05	0.002707296	5836	PYGL	18
GO:0044281	11	small molecule metabolic process	5.193714056	3.63744E-05	0.002707296	6391	SDHC	19
GO:0044281	11	small molecule metabolic process	5.193714056	3.63744E-05	0.002707296	7378	UPP1	22
