# Supplementary material for: Long noncoding RNA GSEC promotes neutrophil inflammatory activation by supporting PFKFB3-involved glycolytic metabolism in sepsis
Source: Cell Death Dis. 2021 Dec 14;12(12):1157. doi: 10.1038/s41419-021-04428-7 (PMC8671582; doi:10.1038/s41419-021-04428-7)
Supplement: Supplementary file 11 — Supplementary Table 9 [file 41419_2021_4428_MOESM11_ESM.pdf]

Supplementary Table 9. 16 significantly changed pathways of the 105 co-expressed mRNAs.

path_id	path_name	Style	path_diffgene_count	path_gene_count	enrichment	pvalue	FDR	-lg(P)
05134	Legionellosis	Up	4	55	46.8035191	7.0127E-06	0.00087658	5.15411709
01100	Metabolic pathways	Up	10	1272	5.05934267	0.00011897	0.0074355	3.924569846
04150	mTOR signaling pathway	Up	4	151	17.0476394	0.00038187	0.01591107	3.418089442
04621	NOD-like receptor signaling pathway	Up	4	168	15.3225806	0.0005758	0.01691705	3.239726431
04664	Fc epsilon RI signaling pathway	Up	3	68	28.3918406	0.00067668	0.01691705	3.16961542
04015	Rap1 signaling pathway	Up	4	210	12.2580645	0.00134738	0.02418156	2.870509735
05132	Salmonella infection	Up	3	86	22.4493623	0.00135417	0.02418156	2.868327604
04620	Toll-like receptor signaling pathway	Up	3	104	18.5638958	0.00236143	0.03660478	2.626824582
04668	TNF signaling pathway	Up	3	108	17.8763441	0.00263554	0.03660478	2.579129657
01200	Carbon metabolism	Up	3	116	16.6434928	0.00324283	0.04053533	2.489076334
04152	AMPK signaling pathway	Up	3	121	15.9557451	0.0036639	0.04163525	2.436056133
04380	Osteoclast differentiation	Up	3	128	15.0831653	0.00430933	0.04488889	2.365589917
04910	Insulin signaling pathway	Up	3	138	13.9901823	0.00534938	0.04846428	2.271696376
05418	Fluid shear stress and atherosclerosis	Up	3	139	13.8895335	0.00546127	0.04846428	2.262706387
00500	Starch and sucrose metabolism	Up	2	36	35.7526882	0.00581571	0.04846428	2.235396978
05206	MicroRNAs in cancer	Down	2	299	33.361204	0.00635338	0.12071414	2.196995455
