# Supplementary material for: Long noncoding RNA GSEC promotes neutrophil inflammatory activation by supporting PFKFB3-involved glycolytic metabolism in sepsis
Source: Cell Death Dis. 2021 Dec 14;12(12):1157. doi: 10.1038/s41419-021-04428-7 (PMC8671582; doi:10.1038/s41419-021-04428-7)
Supplement: Supplementary file 12 — Supplementary Table 10 [file 41419_2021_4428_MOESM12_ESM.pdf]

Supplementary Table 10. 11 upregulated immune response pathways.

path_id	path_number	path_name	enrichment	pvalue	FDR	gene_id	gene_name	gene_number
05134	1	Legionellosis	46.8035191	7.0127E-06	0.00087658	1378	CR1	5
05134	1	Legionellosis	46.8035191	7.0127E-06	0.00087658	4671	NAIP	13
05134	1	Legionellosis	46.8035191	7.0127E-06	0.00087658	58484	NLRC4	14
05134	1	Legionellosis	46.8035191	7.0127E-06	0.00087658	7100	TLR5	20
04621	2	NOD-like receptor signaling pathway	15.3225806	0.0005758	0.01691705	84674	CARD6	4
04621	2	NOD-like receptor signaling pathway	15.3225806	0.0005758	0.01691705	1432	MAPK14	12
04621	2	NOD-like receptor signaling pathway	15.3225806	0.0005758	0.01691705	4671	NAIP	13
04621	2	NOD-like receptor signaling pathway	15.3225806	0.0005758	0.01691705	58484	NLRC4	14
05132	3	Salmonella infection	22.4493623	0.00135417	0.02418156	1432	MAPK14	12
05132	3	Salmonella infection	22.4493623	0.00135417	0.02418156	58484	NLRC4	14
05132	3	Salmonella infection	22.4493623	0.00135417	0.02418156	7100	TLR5	20
04620	4	Toll-like receptor signaling pathway	18.5638958	0.00236143	0.03660478	5608	MAP2K6	11
04620	4	Toll-like receptor signaling pathway	18.5638958	0.00236143	0.03660478	1432	MAPK14	12
04620	4	Toll-like receptor signaling pathway	18.5638958	0.00236143	0.03660478	7100	TLR5	20
04668	5	TNF signaling pathway	17.8763441	0.00263554	0.03660478	8809	IL18R1	10
04668	5	TNF signaling pathway	17.8763441	0.00263554	0.03660478	5608	MAP2K6	11
04668	5	TNF signaling pathway	17.8763441	0.00263554	0.03660478	1432	MAPK14	12
