# Supplementary material for: Long noncoding RNA GSEC promotes neutrophil inflammatory activation by supporting PFKFB3-involved glycolytic metabolism in sepsis
Source: Cell Death Dis. 2021 Dec 14;12(12):1157. doi: 10.1038/s41419-021-04428-7 (PMC8671582; doi:10.1038/s41419-021-04428-7)
Supplement: Supplementary file 13 — Supplementary Table 11 [file 41419_2021_4428_MOESM13_ESM.pdf]

Supplementary Table 11. 6 upregulated metabolic process pathways.

path_id	path_number	path_name	enrichment	pvalue	FDR	gene_id	gene_name	gene_number
04152	1	AMPK signaling pathway	15.9557451	0.0036639	0.04163525	51719	CAB39	3
04152	1	AMPK signaling pathway	15.9557451	0.0036639	0.04163525	5209	PFKFB3	18
04152	1	AMPK signaling pathway	15.9557451	0.0036639	0.04163525	5562	PRKAA1	22
01200	2	Carbon metabolism	16.6434928	0.00324283	0.04053533	5226	PGD	19
01200	2	Carbon metabolism	16.6434928	0.00324283	0.04053533	5230	PGK1	20
01200	2	Carbon metabolism	16.6434928	0.00324283	0.04053533	6391	SDHC	24
04910	3	Insulin signaling pathway	13.9901823	0.00534938	0.04846428	2319	FLOT2	7
04910	3	Insulin signaling pathway	13.9901823	0.00534938	0.04846428	5562	PRKAA1	22
04910	3	Insulin signaling pathway	13.9901823	0.00534938	0.04846428	5836	PYGL	23
01100	4	Metabolic pathways	5.05934267	0.00011897	0.0074355	249	ALPL	1
01100	4	Metabolic pathways	5.05934267	0.00011897	0.0074355	528	ATP6V1C1	2
01100	4	Metabolic pathways	5.05934267	0.00011897	0.0074355	79623	GALNT14	8
01100	4	Metabolic pathways	5.05934267	0.00011897	0.0074355	2992	GYG1	10
01100	4	Metabolic pathways	5.05934267	0.00011897	0.0074355	5226	PGD	19
01100	4	Metabolic pathways	5.05934267	0.00011897	0.0074355	5230	PGK1	20
01100	4	Metabolic pathways	5.05934267	0.00011897	0.0074355	9489	PGS1	21
01100	4	Metabolic pathways	5.05934267	0.00011897	0.0074355	5836	PYGL	23
01100	4	Metabolic pathways	5.05934267	0.00011897	0.0074355	6391	SDHC	24
01100	4	Metabolic pathways	5.05934267	0.00011897	0.0074355	7378	UPP1	27
04150	5	mTOR signaling pathway	17.0476394	0.00038187	0.01591107	528	ATP6V1C1	2
04150	5	mTOR signaling pathway	17.0476394	0.00038187	0.01591107	51719	CAB39	3
04150	5	mTOR signaling pathway	17.0476394	0.00038187	0.01591107	2887	GRB10	9
04150	5	mTOR signaling pathway	17.0476394	0.00038187	0.01591107	5562	PRKAA1	22
00500	6	Starch and sucrose metabolism	35.7526882	0.00581571	0.04846428	2992	GYG1	10
00500	6	Starch and sucrose metabolism	35.7526882	0.00581571	0.04846428	5836	PYGL	23
