# Supplementary material for: Long noncoding RNA GSEC promotes neutrophil inflammatory activation by supporting PFKFB3-involved glycolytic metabolism in sepsis
Source: Cell Death Dis. 2021 Dec 14;12(12):1157. doi: 10.1038/s41419-021-04428-7 (PMC8671582; doi:10.1038/s41419-021-04428-7)
Supplement: Supplementary file 14 — Supplementary Table 12 [file 41419_2021_4428_MOESM14_ESM.pdf]

Supplementary Table 12. Basic clinical data of the included participates.

	Septic patients	Healthy volunteers	P value
Size (n)	20	10	
Age (years)	62.85±7.62	66.40±6.26	0.214
Male (%)	13(65.00)	7(70.00)	0.784
Absolute lymphocyte count (×10^9/L)	0.98±0.32	1.18±0.24	0.085
Absolute neutrophil count (×10^9/L)	15.45±4.39	5.42±0.84	0.000
Absolute eosinophil count (×10^9/L)	0.17±0.01	0.10±0.004	0.084
Eosinophil/ neutrophil (%)	0.12±0.09	0.18±0.07	0.094
SOFA score	12.60±3.79	---	---
APACHE II score	26.70±3.66	---	---
Primary site of infection		---	---
Respiratory system (%)	10 (50.00)	---	---
Digestive system (%)	6 (30.00)	---	---
Urinary system (%)	4 (20.00)	---	---
Pathogenic bacteria of infection		---	---
Gram-negative bacillus (%)	15 (75.00)	---	---
Gram-postive cocci (%)	5 (25.00)		

SOFA: sequential organ failure assessment; APACHE II: Acute Physiology and Chronic Health Evaluation II.
