# Supplementary material for: Long noncoding RNA GSEC promotes neutrophil inflammatory activation by supporting PFKFB3-involved glycolytic metabolism in sepsis
Source: Cell Death Dis. 2021 Dec 14;12(12):1157. doi: 10.1038/s41419-021-04428-7 (PMC8671582; doi:10.1038/s41419-021-04428-7)
Supplement: Supplementary file 15 — Supplementary Table 13 [file 41419_2021_4428_MOESM15_ESM.pdf]

Supplementary Table 13. The targeting sequences (blue) of GSEC shRNAs.

Name	Sequence
Lenti-GSEC-sh1	AAGATTACAAAACAAATTACAAAAATTCAAAATTTTCGGGTTTATTACAGGGACAGCAGAGATCCAGTTTGGTTAATTA<br>ATCGAGCGGCCGCCCCCTTCACCGAGGGCCTATTTCCCATGATTCCTTCATATTTGCATATACGATACAAGGCTGTTAGA<br>GAGATAATTGGAATTAATTTGACTGTAAACACAAAGATATTAGTACAAAATACGTGACGTAGAAAGTAATAATTTCTTG<br>GGTAGTTTGCAGTTTTAAAATTATGTTTTAAAATGGACTATCATATGCTTACCGTAACTTGAAAGTATTTCGATTTCTTGG<br>CTTTATATATCTTGTGGAAAGGACGAAACACCGGGGTCACAACAGTACAAAGACTCGAGTCTTTGTACTGTTGTGACCTT<br>TTTGAATTCTCGACCTCGAGACAAATGGCAGTATTCATCCACGGATCCTAACCCGTGTCGGCTCCAACATAACTTACGGT<br>AAATGGCCCGCCTGGCTGACCGCCCAACGACCCCCGCCCATTGACGTCAATAGTAACGCCAATAGGGACTTTCCATTGA<br>CGTCAATGGGTGGAGTATTTACGGTAAACTGCCCACTTGGCAGTACATCAAGTGTATCATATGCCAAGTACGCCCCCTAT<br>TGACGTCAATGACGGTAAATGGCCCGCCTGGCATTGTGCCCAGTACATGACCTTATGGGACTTTCCTACTTGGCAGTACA<br>TCTACGTATTAGTCATCGCTATTACCATGGTCGAGGTGAGCCCCACGTTCTGCTTCACTCTCCCCATCTCCCCCCCCTCCCC<br>ACCCCCAATTTTGTATTTATTTATTTTTTAATTATTTTGTGCAGCGATGGGGGCGGGGGGGGGGGGGGCGCGCGCAAG
Lenti-GSEC-sh2	ATTCAAAACAAATTACAAAAATTCAAAATTTTCGGGTTTATTACAGGGACAGCAGAGATCCAGTTTGGTTAATTAATCG<br>AGCGGCCGCCCCCTTCACCGAGGGCCTATTTCCCATGATTCCTTCATATTTGCATATACGATACAAGGCTGTTAGAGAGA<br>TAATTGGAATTAATTTGACTGTAAACACAAAGATATTAGTACAAAATACGTGACGTAGAAAGTAATAATTTCTTGGGTA<br>GTTTGCAGTTTTAAAATTATGTTTTAAAATGGACTATCATATGCTTACCGTAACTTGAAAGTATTTCGATTTCTTGGCTTT<br>ATATATCTTGTGGAAAGGACGAAACACCGGCCAACTATGCCATGGTCTTCTCGAGAAGACCATGGCATAGTTGGTTTTT<br>GAATTCTCGACCTCGAGACAAATGGCAGTATTCATCCACGGATCCTAACCCGTGTCGGCTCCAACATAACTTACGGTAA<br>ATGGCCCGCCTGGCTGACCGCCCAACGACCCCCGCCCATTGACGTCAATAGTAACGCCAATAGGGACTTTCCATTGACG<br>TCAATGGGTGGAGTATTTACGGTAAACTGCCCACTTGGCAGTACATCAAGTGTATCATATGCCAAGTACGCCCCCTATTG<br>ACGTCAATGACGGTAAATGGCCCGCCTGGCATTGTGCCCAGTACATGACCTTATGGGACTTTCCTACTTGGCAGTACATC<br>TACGTATTAGTCATCGCTATTACCATGGTCGAGGTGAGCCCCACGTTCTGCTTCACTCTCCCCATCTCCCCCCCCTCCCCAC<br>CCCCAATTTTGTATTTATTTATTTTTTAATTATTTTGTGCAGCGATGGGGGCGGGGGGGGGGGGGGCGCGCGCCA
Lenti-GSEC-OE	CCGTTTTTGGCTTTTTTGTTAGACGAAGCTTGGGCTGCAGGTCGACTCTAGAGGATCCCCGGGTACCGGT<br>TAGAAAGGGCGGGGTGGAGGAGGGGGAAGGGCGGGGGTGATGCCGCGCGGTCGCAGGCTTGGGATGG

	TGTTCGCGCCTCCGAGACCCGGACAGAGGCAAGCAGGGGCGCCGTGGGTGCCAGAGAGGCGGAAGAGG<br>AGGCCTGATGGGGTCAGCACAGTGTTTCCGGAGCCCCACGTATCAACAGCCCAGTGCCGGGAGCGCCAG<br>GGGCTCCAAGGAAACTATCAGACCCATTCCCTGTCCCCAGGAAGCTTACCATCCAGCGGCACAGAGAAG<br>GCTCACCATGGACTCCATTCAAGAACGAGGCAGGATAGGATGTGAATAAGTGCAAGGTGTGAGTGACT<br>GACAGTAAGTGCAAGGGGGTTCAGAGAAGTGGCAGCTCCAAGCCTGCTTCTGCTGAAGCTGGAGGGCTT<br>ATCTGTCAAGAACCCCCAACTCAGGAAGCTCTGCTCCAAGTGAGCTGAGTAAGGAGAGTCTGCCTCCAA<br>GTTGTTCCTCTGCAGGTGACTGTGCTCCATGCTAAATCATTCCTTGCACGGGCAGAAGCTGTTTGGCATT<br>GCACCAGTGTCTGATTTTGTTTGAATATGGTGCCTTTGGCTTAATCAGTCAGACATGCATTCAACAACTA<br>TTTATTTATTTATTTATTTATTTATTTATTTATTATTTTGAGACAGAGTCTCGCTCTGTCGCCAGGCGGAG<br>GTTGCAGTGAGCCGAGATTGAGCCACTGCACTCCAGCCTGGCAACAGAGTGAGACTGTCTCAAAAAGTA<br>AATAAATAGAAACTCTTGGTTCCATGCGTAGTCTAGCATTTTGTAGCACTTCTAATCACGAAGTTATTTT<br>TAATATTTGTTTGGTCTGTTATACAAAATGTAGACTGGATGGCTTATAAACTACAGAAATCTCTCACAGT<br>TCTGTAGGCTAGAAAGTCTAAGATGAAGATGCCAGCAGCCTGCTTCCTGGTTCATACAAGGTCATCTTCT<br>CACTGTAACCTCACAGTGAGAGGAAGGGCCTAGGGGTGACTCTGGGATCTCTTTTATGAGGCACTAATC<br>CCATTAATGGGGGCCCCCACCCTCATGGCCTAATCACCTCCCAAAGGCCCCACGATTGCCTTGGGTGTT<br>AGGATTTCCACATATGAATTTTAGGGGAACACAGACGTTCAGGCCATAGCAAATATCTTGCCCAAATAT<br>CTCTAGTTCCATCCTTTTGGTCACTCTCAGACCAGAACATTTACTGAGTGATTACTATGTCCCACAAAAT<br>TCTTATTTAACCCTCAGAAAAACCGTCTGGGGGAAGCATTGTTATCACTCCTATTTCATGAGCAGTTATG<br>TAACTTGCCCCAGATAAGACTTGGTTATGGCACAGATTCTGTGTTGGAGCCTTCAGACCACTCTACTTCC<br>CAAGCAGGGGAGGAACAGAACATTAAAGATTTTCTTGGCCTTGGCTTTCATTTCTTCATCGTGGTTCTGA<br>AAGGAAAATTGGCTACAGTCTTAAATGTATGTGTGGGCCCACGCTAGAAGCTCAGCTCTCAGTGCCAGC<br>CACACTGAGCCCTGGTCTCCCTAACTTCCGGGCCTTTACTCTGCTTGTCATTGGGCTGATAACCAAGATC<br>CAGCCTAGAGCACAAGAAGACAAACGCTGCGTGATCTCGTGTGTAGAACCTAACAAAGTGGAACTCAC<br>AGAAGCAGAGAGTAGAATGGTGGTTGCCTGTTGGAATGGGGAGATGGTCAGAGAATGCAAACTTTCAG<br>TTGGATGGGAGGAAGAAGTTCCAGAGATCTATTGTGCCACATGGTGACAATAGTTAATAACAATGTACT<br>GGGCGGGGGGTGGTGGCCTACGCCTGTAATCCCAGCACTTTGGGAAGCTGAGGCAGACAGATCACTTGA<br>GGTCAGGAGTTCTAGACCAGCTTGTCCAACATGGTGAAACCCCGTCTCTACTATAAAACTACAAAATTA<br>GCCAGGTGTGGTGGTGTGCGCcTGTAATCTCAGCTACTTGGGAGGCTGAATCGCTTGAACCCAGGAGGT

	GGAGGTTGCAGTGAGTCGAGACTGCGCCACTGCCCTCCAGCCTGGGTGACACAGTGAGACTCTGTTTCA<br>AAAAAACAAAAAAAGCAATGTACTGTATACTTGAAAATTGCTCACAGAGATTTTAAATGTTCTCACCAT<br>TACAAAATGATAAATATGTGACATAATGCATATGTTAATTAGCCCGATTTAGTGATTCCACAGTGTATAC<br>ATATAACAAAACATGTTGTACACCATAAATGTATACTATTTTTATTTGCCAATTAAATATATTTTTTTAAA<br>GTGGATACCTTCCTGCTGTCCTTCCTGAGCACAACCTGGCTGAAAACCTGGAGGTCACAACAGTACAAA<br>GAATCAAAGTCAAGATCTTGTGCCAGAGAGCAAATGAACTCTTCCTCTTGCTGAGAAAACCCACCCTGC<br>TCACCTAAACCCTGGCCTTGCCTGGTAATTCCATCCATGCGCCTGGAAGGCCCCAGACATCAAGGCTCTG<br>AGGGGCCAGGCACGGGGAGAACCCAGCAGTGCCCTGCCCTGCAGTCTGAGCTACCAGATTCCTTGTGAA<br>GATAATTTGAGGACCATGACTCACCCAACCACATTTCCTGGGGCCTCAAATTGAAAATTCAGGATGGGC<br>TTTTCTATATGACTGGCTGATATCCAACTATGCCATGGTCTTTACATGCCATGAACATTCTTTCCTGCCAG<br>AGTTCTAAGAATCTGTGTTCTCTGCCTTAGACCTTCTGCAGATGAGCCCACAGGAAGCTCCACGTGTAGC<br>TGAGCTACATGCACCAGGCCTCAGTTTGCCCCAAGTCCCCTGTGTACTCTCTCATGGCCTGTGGCCAAGA<br>AATGTATTCTCTCACTTTGGACTTAGGAGTCCAAAGAGAAGCCCAGAAACAAAATTGCTTGAACTTGAA<br>TTTGTGTGCGTGCGCACGTGTGCACGTGGTGGTGAAGGTGTATGTTTTCGGCTGTTCTATGCGTCACTGT<br>CACCAAACTCCCAAATAATAGTAACATTTGTTTAGATGATGTCTGCTGACAAATCACAAACACGACGCT<br>AACTCGCAACTCTCTGCTCCACTGGCACAGAATAGGGCATGGAGCCTGGTGCTGGGTGTCAGCCCATGG<br>TGTTGGGTGTCAGTTCACAGGCTGGGTAAGGGAGGGAAAATAATCCATTCTTTGATATTAGACATGACC<br>CAAAATTTCCTGCTGGCAGCCAAAGGCCTCCTCGCTCAGAGAAGTCATCTGAAAAAAGCTAGCCCAGGG<br>GCAGGAAAGGGCCTCAGGCTGGCGCCCCAGAAGGTGGCCCATCAGTCACTCTGGGAAGACAGATAGAC<br>ATCGTCAGTCTCTTTTTACAAGTCAAGACAGTAAAATCAAAGTAATAGTTTCCTGGCAGGAAGAAAGAG<br>AATTGCTGGAGCGTTGGAGAGCCATCTCTTAACCTCTGGGGTGACTCAAAGGAAGAGTTGCCTGGTCTG<br>AGAACTTTAGACCAAAGAATTTAAATCATGTGAACTGAAAATGCATGTGTATCCATATATACATGTACA<br>TATATGTTTATATACCCAATATAGAAAGGTTACCAGAGACAAAGAAATAGCAACAATAGTTGTTTTGGG<br>GAAGGGAAAGGAAGGGCTGTGGGGTGAGGAAGCATACTTTCACTGAATATCGCTAGCCTGTGGAATGT<br>GTGTCAGTTAGGGTGTGGAAAGTCCCCAGGCTCCCCAGCAGGCAGAAGTATGCAAAGCATGCATCTCAA<br>TTAGTCAGCAACCAGGTGTGG
